# Magnesium Cyanide or Isocyanide?

**DOI:** 10.1002/anie.201909511

**Published:** 2019-09-18

**Authors:** Gerd Ballmann, Holger Elsen, Sjoerd Harder

**Affiliations:** ^1^ Inorganic and Organometallic Chemistry Universität Erlangen-Nürnberg Egerlandstrasse 1 91058 Erlangen Germany

**Keywords:** alkaline-earth metal, cyanide, DFT calculations, isocyanide, magnesium

## Abstract

Preference for the binding mode of the CN^−^ ligand to Mg (Mg−CN vs. Mg−NC) is investigated. A monomeric Mg complex with a terminal CN ligand was prepared using the dipyrromethene ligand ^Mes^DPM which successfully blocks dimerization. While reaction of (^Mes^DPM)MgN(SiMe_3_)_2_ with Me_3_SiCN gave the coordination complex (^Mes^DPM)MgN(SiMe_3_)_2_⋅NCSiMe_3_, reaction with (^Mes^DPM)Mg(*n*Bu) led to (^Mes^DPM)MgNC⋅(THF)_2_. A Mg−NC/Mg−CN ratio of ≈95:5 was established by crystal‐structure determination and DFT calculations. IR studies show absorbances for CN stretching at 2085 cm^−1^ (Mg−NC) and 2162 cm^−1^ (Mg−CN) as confirmed by ^13^C labeling. In solution and in the solid state, the CN ligand rotates within the pocket. The calculated isomerization barrier is only 12.0 kcal mol^−1^ and the ^13^C NMR signal for CN decoalesces at −85 °C (Mg−NC: 175.9 ppm, Mg−CN: 144.3 ppm). Experiment and theory both indicate that Mg complexes with the CN^−^ ligand should not be named cyanides but are more properly defined as isocyanides.

## Graphical abstract

**The two faces of CN**: Cyanide ligands are generally C‐bound in transition‐metal chemistry, but for Mg, an isocyanide structure was established. Based on experiment and theory, at 298 K, a Mg−NC/Mg−CN ratio of 95:5 is found. In the solid state and in solution, isomerization is facile with a low energy barrier.

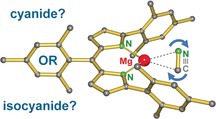

First reports on metal‐cyanide chemistry date back to the serendipitous discovery of Prussian Blue, Fe_7_(CN)_18_, in 1704 by the Berlin painter Diesbach.^1^ Like all transition‐metal cyanide complexes, this famous blue pigment is extremely stable and can only be destroyed by strong acids or carbon monoxide, a ligand isoelectronic to cyanide and one of few that can compete with cyanide in the spectrochemical series for ligand strength.^2^ Although the negative charge in C≡N^−^ is mainly located at the N,^3^ in the vast majority of transition‐metal cyanide complexes the cyanide ligand is C‐bound. This strong preference for cyanide vs. isocyanide formation is due to the HOMO (lone pair) which has a large coefficient at the C.^4^ Although d→π* backbonding to the negatively charged C≡N^−^ ligand is less prominent than that to neutral C≡O, it is not negligible and increases the donor strength at the N. This explains its strong tendency to bridge metals, forming inclusion compounds with a large variety of applications.^1^, ^5^

In contrast to the wealth of highly stable transition metal complexes stands the chemistry of s‐block metal cyanides. While the badly reputed alkali‐metal cyanides are important bulk chemicals, very little is known about Group 2 metal cyanides.^6^ The frustrations in first attempts to isolate Mg(CN)_2_ are clearly described by Fichter and Suter.^7^ Magnesium metal reacts rapidly with a 25 % solution of hydrogen cyanide in water [Eq. 1], however, isolation of Mg(CN)_2_ by evaporation of the solvent (and volatile HCN) resulted in the formation of Mg hydroxides [Eq. (2)]. Since HCN is a weak acid, the cyanide anion is a relatively strong base that can deprotonate water, epecially when this is acidified by coordination to a strong Lewis acid like Mg^2+^. Using liquid ammonia as a reaction medium circumvents this problem and led to first preparations of pure Mg(CN)_2_.^8^

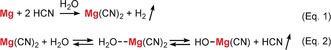



Alkali‐metal cyanides form rock‐salt‐like structures, for example, KCN (Phase I) crystallizes in the NaCl lattice and down to −100 °C, the cyanide anion rotates in a cage spanned by six K^+^ ions.^9^ This essential isotropic coordinative behavior of the spinning cyanide anion is typical for ionic metal cyanides and explains its description as a pseudohalide. Calculations on MCN (M=Li, Na, K) show that an orbiting motion of M^+^ around CN^−^ is essentially barrier‐free (<5 kcal mol^−1^).^10^

More covalently bound main‐group CN compounds generally prefer cyanide connectivity. For example, organic nitriles (RCN) are thermodynamically more stable than the corresponding isonitriles (RNC).^11^ The crystal structure of B(CN)_3_⋅pyridine shows CN/NC disorder with a main contribution of the cyanide form (B−CN/B−NC=95:5).^12^ Likewise, the anion [(CF_3_)_3_B−CN]^−^ is 8.4 kcal mol^−1^ lower in energy than [(CF_3_)_3_B−NC]^−^.^13^ Trimethylsilyl cyanide, Me_3_SiCN, was shown to contain small but significant quantities of Me_3_SiNC.^14^ Experimental and calculation data indicate that the X−CN/X−NC ratio increases with increasing electronegativity of X, that is, the cyanide isomer becomes more favorable for covalently bound CN groups (Scheme 1 a).^13^, ^14^, ^15^, ^16^, ^17^, ^18^ For ionically bound CN^−^, for example, LiCN, the cyanide/isocyanide energy differences become negligible while, at the same time, the transition states for isomerization are lowered as well.

**Scheme 1:**
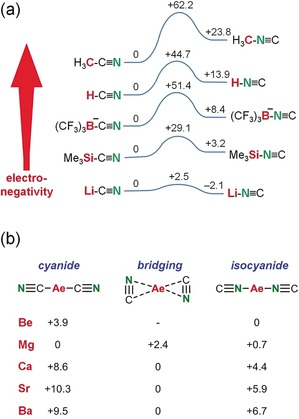
a) Simplified energy profiles (kcal mol^−1^) for cyanide‐to‐isocyanide isomerization from the literature: MeCN (exp.),^15^ HCN (calc.),^16^ (CF_3_)_3_BCN (exp.),^13^ Me_3_SiCN (calc.),^14^ LiCN (calc.).^16^ b) Relative energies (kcal mol^−1^) for Ae(CN)_2_ complexes (MP4SDTQ//MP2 including ZPE, true minima with no imaginary frequencies); values taken from ref. 19.

The alkaline‐earth‐metal cyanides, Ae(CN)_2_, are hardly explored. Being more covalent than Group 1 metal cyanides, higher transition states for isomerization are expected. The crystal structure of monomeric Be(CN)_2_⋅(pyridine)_2_ shows Be−CN/Be−NC disorder with a ratio of 40:60.^12^ High‐level ab‐initio calculations (MP4SDTQ//MP2) on Group 2 metal cyanides predict unusual features.^19^ While the small and hard Be^2+^ cation prefers the N‐bound isocyanide structure, the heavier Ca^2+^, Sr^2+^, and Ba^2+^ ions are neither cyanides nor isocyanides but instead prefer a side‐on coordination (Scheme 1 b). Our group reported the first Ca‐cyanide complex (**I**) which is stabilized for ligand exchange by the bulky ß‐diketiminate ligand ^DIPP^BDI.^20^ Jones and co‐workers described the formation of a similar, THF‐free Mg complex (**II**).^21^ The bridging cyanides in both trimers are statistically disordered and, like in related Al chemistry,^22^ their bridging nature does not allow any conclusions regarding cyanide vs. isocyanide coordination. Surprisingly, in some reports on rare examples of terminally bound metal isocyanides, the cyanide/isocyanide isomerism is not even subject of discussion.^23^, ^24^, ^25^ Since there is a broad interest in metal‐CN isomerism from a theoretical^26^ or experimental^27^ point of view, we here report the synthesis and structure of a Mg complex with a terminal CN^−^ ligand and provide a first comprehensive discussion on (iso)cyanide preference.


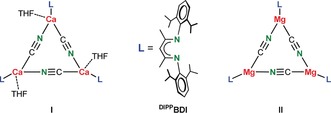


To prepare a monomeric Mg cyanide complex, we switched from the ß‐diketiminate ligand (BDI) to a dipyrromethene ligand (DPM). DPM is a subunit of porphyrin and, although already known since 1924,^28^ has only been sporadically used in Group 2 metal chemistry.^29^, ^30^ The DPM ligand is substantially more sterically demanding than the BDI ligand and noticeably encapsulates the metal by its flanking substituents that form a cavity wich prevents dimerization.

Deprotonation of ^Mes^DPM−H^31^ with Mg[N(SiMe_3_)_2_]_2_ gave (^Mes^DPM)MgN(SiMe_3_)_2_ (**1**) in excellent yield (Scheme 2). Attempted amide–cyanide substitution by reaction with Me_3_SiCN, however, only led to a coordination complex (**2**) that did not react further, also at higher temperatures. Deprotonation of ^Mes^DPM−H with Mg(*n*Bu)_2_ gave the much more reactive alkylmagnesium complex (^Mes^DPM)Mg(*n*Bu) (**3**) which, in toluene, reacted with Me_3_SiCN already at −70 °C to give the desired Mg‐cyanide complex (**4**) that was crystallized from toluene/THF in 57 % yield. All complexes have been fully characterized by crystal‐structure determination (**4** is shown in Figure 1 a; see Supporting Information for **1**–**3**).

**Figure 1:**
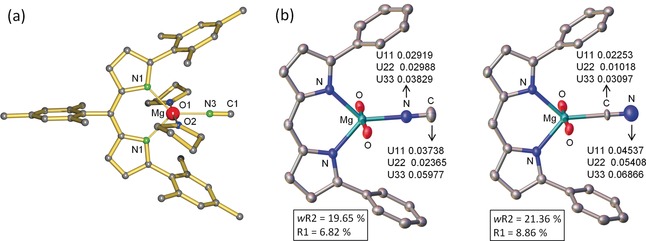
a) Crystal structure of (^Mes^DPM)MgNC⋅(THF)_2_ (**4**); H atoms omitted for clarity. Selected bond distances (Å) and angles: Mg‐N1 2.098(2), Mg‐N2 2.092(2), Mg‐N3 2.049(2), Mg‐O1 2.136(2), Mg‐O2 2.131(2), N1‐Mg‐N2 92.88(8), N1‐Mg‐N3 133.25(9), N2‐Mg‐N3 133.87(8), O1‐Mg‐O2 176.55(7). b) ORTEP representations (50 % probability) of the complex as the Mg−NC (left) and the Mg−CN isomer (right). Refinement as the Mg−NC isomer gave lower R‐values and more realistic displacement factor.

**Scheme 2:**
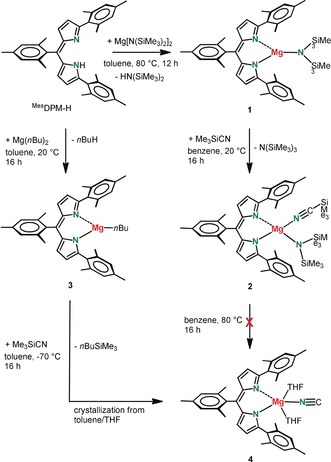
Synthesis of the Mg isocyanide complex **4**.

The Mg metal in **4** displays a distorted trigonal bipyramidal coordination geometry with axial THF ligands. The N1−Mg−N2 bite angle of 92.88(8)° deviates from the ideal equatorial angle of 120° but is in the range of values found in other DPM magnesium complexes.^29^, ^30^ Single‐crystal X‐ray diffraction also shows significant signals at high 2*θ* angles. An isocyanide arrangement, Mg−N≡C, was clearly confirmed by refinement of the alternative cyanide connectivity, Mg−C≡N. The latter did not only give much higher *w*R2 and R1 values but also showed an unrealistically high displacement parameter for the terminal N atom whereas those for the Mg‐bound C were too low (Figure 1 b). Refinement of the structure with an isocyanide/cyanide disorder model only led to small contributions of the cyanide arrangement (<8 %).

DFT calculations using the B3PW91(D3BJ)/6‐311+G** method (including D3BJ dispersion corrections)^32^ on a model system in which all mesityl substituents have been replaced by phenyl rings reproduce the crystal structure of **4** remarkably well; for example, *d*(Mg−NC)=2.049(2) Å (X‐ray) and 2.039 Å (DFT; Figure 2). The calculated Mg−CN bond length in the Mg cyanide isomer is considerably higher (2.158 Å), providing further confirmation for the presence of the Mg−NC isomer in the crystal structure. The Mg−CN isomer is also higher in energy by Δ*G*(298 K)=1.63 kcal mol^−1^. This energy difference translates to a Mg−NC/Mg−CN ratio of 94:6, which is close to the experimentally determined ratio of 92:8 from the crystal‐structure data. Interestingly, this result also compares extremely well with the 95.5:4.5 ratio for low‐valent MgNC/MgCN radicals discovered in the envelope of C‐rich stars for which an energy difference of 1.88 kcal mol^−1^ was calculated in favor of MgNC.^33^

**Figure 2:**
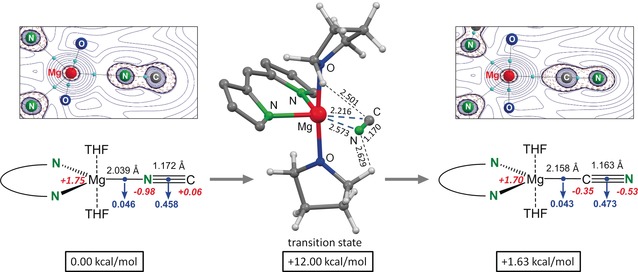
DFT calculations (B3PW91(GD3BJ)/6‐311+G**), Δ*G* at 298 K. Selected bond distances (black, in Å), NPA charges (red), and charge densities in the bond‐critical points (blue, in a.u.). Insets show contour plots of the Laplacian of the electron density (atoms in molecules).

Strong Mg−NC bonding in the isocyanide isomer is not only apparent from a short Mg−N distance but also from the electron density at the bond‐critical point (BCP) which is slightly higher than that for the cyanide isomer. Consequently, the C≡N bond in the isocyanide complex is somewhat longer and weaker than that in the cyanide isomer. Calculated NPA charges show that the isocyanide anion is extremely polarized with a high negative charge on the Mg‐bound N (−0.98) while the cyanide anion has a much lower charge on Mg‐bound C (−0.35); calculated charges for free C≡N^−^ are −0.24 (C) and −0.76 (N). Strong preference for the isocyanide isomer is therefore related to larger electrostatic contributions and polarization. Contour plots of the Laplacian of the electron density (atoms in molecules) clearly show that, although the cyanide ligand itself is much less polarized than the isocyanide ligand, the lone pair at the C is considerably better polarizable than that on the N. Therefore, the preference for the Mg−N bonding may also be explained by the hard‐soft‐acid‐base theory (HSAB): the hard Mg atom prefers interaction with the hard N atom.

Calculations on the very simple model system HMg(NC) provided valuable insight into the complicated CN/NC isomerization process (Supporting Information, Figure S24). Similar to the LiCN/LiNC isomerization,^10^ two transition states and one intermediate minimum were located. Only one transition state was found for isomerization of the larger model system (^Ph^DPM)Mg(NC)⋅(THF)_2_ (Figure 2). The barrier of 12.0 kcal mol^−1^ for rotation of the CN^−^ anion agrees well with that of 10.2 kcal mol^−1[26c]^ calculated for Mg(CN)_2_ and suggests that isomerization is facile. Non‐classical C−H⋅⋅⋅N and C−H⋅⋅⋅C hydrogen bonds between the CN^−^ anion and the THF ligands contribute to the stability of the transition state.

The infrared (ATR‐IR) spectrum of **4** in the solid state shows a sharp but relatively weak signal at 2084 cm^−1^ for the CN stretching vibration (Figure 3 a). This fits very welll with the calculated value for **4** of 2099 cm^−1^ (B3PW91/6‐311+G(2df,p), Figure S28). A much higher frequency of 2166 cm^−1^ was calculated for the alternative Mg−CN isomer. The C≡N IR stretching frequencies for cyanides are generally 70–100 cm^−1^ higher than those for isocyanides, which is in accordance with their shorter CN bonds.^13^ This is also in agreement with our calculations which show a shorter CN bond and a higher electron density at the BCP for the Mg−CN isomer. Interestingly, the solid‐state ATR‐IR spectrum of **4** also shows a very small signal at 2161 cm^−1^, a value close to that calculated for the Mg−CN isomer (2166 cm^−1^). Heating the ATR sample holder to 70 °C led to intensity changes and additional signals only in the CN spectral range (Figure S20), indicating that a fast rotation of the CN ligand may take place. An IR spectrum of **4** in a KBr pellet (Figure 3 b) shows the CN absorbances for Mg−NC (2085 cm^−1^) and Mg−CN (2162 cm^−1^) more clearly. ^13^C‐labeling of the CN ligand confirms their origin: signals for the isotope labeled complex appeared at the expected frequencies of 2043 cm^−1^ (Mg−N^13^C) and 2118 cm^−1^ (Mg−^13^CN; Table S6). These data are in agreement with the X‐ray and DFT studies which both predict minor quantities of the cyanide isomer. Since both isomers cannot be obtained in pure form, further quantification by IR is excluded. It should be noted, however, that exchange of ^12^CN for ^13^CN results in a lower Mg−NC/Mg−CN ratio. The increased cyanide content may be explained by the stronger Mg−^13^CN bond (vs. Mg−^12^CN) while the Mg−NC bond is less affected by isotope substitution.

**Figure 3:**
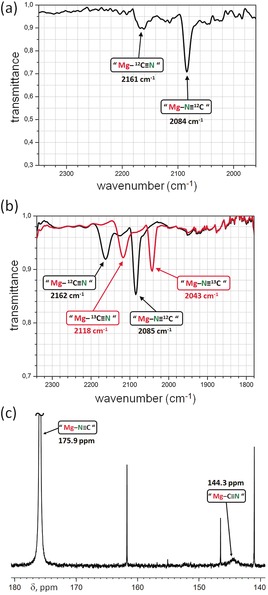
a) ATR‐IR spectrum of solid (^Mes^DPM)Mg(NC)⋅(THF)_2_ (**4**). b) IR spectra of **4** in KBr pellets (the red trace shows the spectrum for the ^13^CN isotope). c) ^13^C NMR spectrum of **4** (^13^C‐enriched CN) in [D_8_]THF at −85 °C showing separate signals for the Mg−NC and Mg−CN isomers.

^1^H NMR data and DOSY measurements on **4** dissolved in [D_8_]THF confirm that the highly symmetric monomeric solid‐state structure is retained in solution (Figures S9 and S14). While all resonances in the ^13^C NMR spectrum can be assigned, no signal for the CN ligand is observed. However, the ^13^CN‐enriched complex shows a broad resonance at 169.2 ppm which is in the typical range for isocyanide isomers: the cyanide C resonance is typically found in the 95–145 ppm range while for isocyanides, values around 155–175 ppm are common.^13^ Heating the solution led to signal sharpening and a shift to lower ppm values indicative of Mg−NC‐to‐Mg−CN isomerization. Stepwise cooling lead to signal broadening and a gradual shift of the ^13^CN signal to higher ppm values. At −85 °C, decoalescence is reached and a second, much smaller, broad signal at 144.3 ppm appears, which is typical for a cyanide group (Figure 3 c). The main signal, assigned to Mg−NC, is found at 175.9 ppm, that is, at the higher end of the range for isocyanide complexes. This clearly shows that the Mg isocyanide and cyanide isomers are in fast equilibrium. Temperature lowering increases the Mg−NC/Mg−CN ratio and results in slow exchange. Due to different ^13^C relaxation times in both isomers, no exact ratio has been estimated. It is, however, clear that the Mg−NC/Mg−CN ratio is large. Knowing the chemical shift of pure Mg−NC (175.9 ppm) and Mg−CN (144.3 ppm), however, enables an estimation of the Mg−NC/Mg−CN ratio at room temperature. The ^13^C NMR signal at 169.2 ppm (298 K) is the weighted average from which a Mg−NC^13^/Mg−^13^CN ratio of 79:21 can be deduced. For cyanide with a natural isotope distribution, this value will be higher (see above).

We have shown that the dipyrromethene ligand ^Mes^DPM succesfully blocks dimerization, enabling the isolation of a Mg complex with a terminal CN ligand. Crystal structure determination as well as IR and NMR studies show a clear preference for the isocyanide isomer: at 298 K a ratio of ≈95:5 is estimated. Due to a relatively low isomerization barrier of only 12.0 kcal mol^−1^, rotation of the CN ligand within the pocket can be observed in solution as well as in the solid state. ^13^C NMR studies in solution show that the isocyanide/cyanide exchange can be frozen at −85 °C, leading to decoalescence of ^13^C NMR signals for the CN ligand. Experiment and theory both indicate that Mg complexes with the CN^−^ ligand should not be named cyanides but rather be referred to as isocyanides. Clear preference for Mg isocyanide formation should be taken into account when discussing the mechanism of Mg‐catalyzed aldehyde or ketone cyanation.^34^

## Conflict of interest

The authors declare no conflict of interest.

## Supporting information

As a service to our authors and readers, this journal provides supporting information supplied by the authors. Such materials are peer reviewed and may be re‐organized for online delivery, but are not copy‐edited or typeset. Technical support issues arising from supporting information (other than missing files) should be addressed to the authors.

Supplementary
