# Supplementary material for: Magnesium Cyanide or Isocyanide?
Source: Angew Chem Int Ed Engl. 2019 Sep 18;58(44):15736–41. doi: 10.1002/anie.201909511 (PMC6856803; doi:10.1002/anie.201909511)
Supplement: Supplementary file 1 — Supplementary [file ANIE-58-15736-s001.pdf]

Angewandte Chemie
Eine Zeitschrift der Gesellschaft Deutscher Chemiker

Supporting Information

Magnesium Cyanide or Isocyanide?

*Gerd Ballmann, Holger Elsen, and Sjoerd Harder**

anie_201909511_sm_miscellaneous_information.pdf

SUPPORTING INFORMATION

Contents

1) General experimental procedures and complex syntheses S 2

2) Crystal structure data S 5

3) Selected ^{1}H, ^{13}C, DEPT-135, ATP, COSY, HSQC,

HMBC and DOSY NMR spectra S12

4) Selected infrared spectra S28

5) Theoretical calculations S35

6) References S56

1) General experimental procedures and complex syntheses

General experimental procedures

All experiments were carried out in dry glassware under N_2 using standard Schlenk techniques and freshly dried and degassed solvents (all solvents were dried over a column except for THF, which was dried over Na and redistilled). Starting materials and research chemicals were obtained from commercial suppliers where appropriate and used without further purification. (^{Mes}DPM-H)[1], $Mg(N(SiMe_3)_2)_2$,[2] and TMS-^{13}CN[3] were synthesized following literature known procedures. NMR spectra were measured on Bruker Avance III HD 400 MHz and Bruker Avance III HD 600 MHz spectrometers. Chemical shifts (*δ*) are given in ppm (parts per million) values, coupling constants (*J*) in Hz (Hertz). For describing signal multiplicities common abbreviations were used: s (singlet), d (doublet), t (triplet), q (quartet), m (multiplet) and br (broad). Spectra were referenced due to solvent residual signal. IR vibrational spectra were recorded on a Shimadzu, IRAffinity-1 as KBr pellets at room temperature or neat on a Bruker Alpha II Platinum ATR. Elemental analysis was performed with a Hekatech Eurovector EA3000 analyzer. All crystal structures have been measured on a SuperNova (Agilent) diffractometer with dual Cu and Mo microfocus sources and an Atlas S2 detector. Crystallographic data have been deposited with the Cambridge Crystallographic Data Centre as supplementary publication numbers: 1942970 (**1**), 1942971 (**2**), 1942972 (**3**) and 1942973 (**4**).

Complex syntheses

Synthesis of (^{Mes}DPM)MgN(SiMe$_3$)$_2$ (1)

In a 50 mL Schlenk flask ^{Mes}DPM-H (580 mg, 1.16 mmol) was suspended in toluene (12 mL). $Mg(N(TMS)_2)_2$ (421 mg, 1.22 mmol, 1.05 equiv.) was added to the stirred yellow reaction mixture resulting in a color change to red/orange. The reaction mixture was heated to 80 °C for 12h and subsequently filtered, which gave a dark orange solution. The solvent was removed in vacuo yielding a light orange powder. Recrystallization from a concentrated toluene solution at -20 °C yielded orange crystalline blocks. Yield: 744 mg (1.09 mmol; 94 %).

^{1}H NMR (400 MHz, C_6D_6, 25 °C): δ = 6.84 (s, 4H, Mes-aryl-C*H*), 6.83 (s, 2H, Mes-aryl-C*H*), 6.82 (s, 2H, Mes-aryl-C*H*), 6.74 (d, *J* = 3.9 Hz, 2H, pyrrole-C*H*), 6.13 (d, *J* = 3.9 Hz, 2H, pyrrole-C*H*), 2.26 (s, 6H, Mes-C*H$_3$*), 2.25 (s, 12H, Mes-C*H$_3$*), 2.24 (s, 3H, Mes-C*H$_3$*), 2.18 (s, 6H, Mes-C*H$_3$*), -0.05 (s, 18H, N(Si(C*H$_3$*)$_3$)$_2$) ppm.

^{13}C{^{1}H} NMR (101 MHz, C_6D_6, 25°C): δ = 162.7 (C_{arom}), 147.0 (C_{arom}), 141.5 (C_{arom}), 138.6 (C_{arom}), 137.4 (C_{arom}), 136.9 (C_{arom}), 136.8 (C_{arom}), 136.5 (C_{arom}), 133.8 (C_{arom}), 133.5 (C_{arom}), 129.0 (C_{arom}), 128.2 (C_{arom}), 119.9 (C_{arom}), 21.2 (C_{aliph}), 21.2 (C_{aliph}), 21.0 (C_{aliph}), 20.0 (C_{aliph}), 4.5 (C_{aliph}).

Elemental analysis: Calculated values for $C_{42}H_{55}N_3Si_2Mg$ (681.38 g/mol): C 73.92, H 8.12, N 6.12; Found: C 73.94, H 8.08, N 6.07.

Synthesis of (^{Mes}DPM)Mg(N(SiMe$_3$)$_2$)(NC-TMS) (2)

In a 50 mL Schlenk flask (^{Mes}DPM)MgN(SiMe$_3$)$_2$ (**1**) (346 mg, 0.508 mmol) was dissolved in benzene (20 mL) to give a dark orange solution. A solution of trimethylsilyl-cyanide (64 µL, 50 mg, 0.508 mmol, 1.0 equiv.) in benzene (2 mL) was added slowly to the stirred reaction mixture at -70 °C. The reaction mixture was kept under inert atmosphere and was warmed to room temperature overnight. The solvent was removed in vacuo yielding a reddish powder. Recrystallization from a concentrated toluene solution at -20 °C yielded orange crystalline blocks. Yield: 191 mg (0.245 mmol; 48 %).

^{1}H NMR (600 MHz, C_6D_6, 25 °C): δ = 6.85 (s, 2H, Mes-aryl-C*H*), 6.81 (d, *J* = 3.9 Hz, 2H, pyrrole-C*H*), 6.79 (s, 4H, Mes-aryl-C*H*), 6.16 (d, *J* = 3.9 Hz, 2H, pyrrole-C*H*), 2.35 (s, 6H, Mes-CH_3), 2.32 (s, 12H, Mes-CH_3), 2.25 (s, 3H, Mes-CH_3), 2.20 (s, 6H, Mes-CH_3), -0.09 (s, 18H, N(Si(CH_3)$_3$)$_2$) , -0.12 (s, 9H, N≡C−Si(CH_3)$_3$) ppm.

^{13}C{^{1}H} NMR (151 MHz, C_6D_6, 25°C): δ = 162.2 (C_{arom}), 147.0 (C_{arom}), 141.7 (C_{arom}), 137.7 (C_{arom}), 137.5 (C_{arom}), 137.4 (C_{arom}), 137.3 (C_{arom}), 137.2 (C_{arom}), 137.0 (C_{arom}), 134.7 (C_{arom}), 133.3 (C_{arom}), 128.5 (C_{arom}), 128.4 (C_{arom}), 119.2 (C_{arom}), 21.5 (C_{aliph}), 21.2 (C_{aliph}), 21.2 (C_{aliph}), 20.4 (C_{aliph}), -6.4 (C_{aliph}), -2.9 (C_{aliph}) ppm.

Elemental analysis: Calculated values for $C_{46}H_{64}N_4Si_3Mg$ (780.61 g/mol): C 70.69, H 8.25, N 7.17; Found: C 69.64, H 8.17, N 6.64.

Synthesis of (^{Mes}DPM)Mg(*n*Bu) (3)

A 50 mL Schlenk flask was charged with ^{Mes}DPM-H (1.16 g, 2.33 mmol) suspended in toluene (25 mL) and cooled to -70 °C. Dibutylmagnesium (1M in *n*-heptane, 2.44 mL, 2.44 mmol, 1.05 equiv.) was added slowly, resulting in a color change from yellow to red. The reaction mixture was kept under inert atmosphere and was warmed to room temperature overnight. Removal of solvent in vacuo gave the crude product as red solid in quantitative yield. Recrystallization in a concentrated toluene solution at -20 °C yielded a first crop of crystalline red blocks. Second crops of crystals contained higher levels of impurities. Yield: 311 mg (0.538 mmol; 23 %).

^{1}H NMR (600 MHz, C_6D_6, 25 °C): δ = 6.87 (s, 2H, Mes-aryl-C*H*), 6.82 (s, 4H, Mes-aryl-C*H*), 6.77 (d, *J* = 3.9 Hz, 2H, pyrrole-C*H*), 6.19 (d, *J* = 3.9 Hz, 2H, pyrrole-C*H*), 2.30 (s, 6H, Mes-CH_3), 2.26 (s, 3H, Mes-CH_3), 2.17 (s, 12H, Mes-CH_3), 2.15 (s, 6H, Mes-CH_3), 1.04 - 0.10 (m, 2H, Mg−γ-CH_2), 0.89 - 0.94 (m, 5H, Mg−β-CH_2 and Mg−δ-CH_3), -0.75 - -0.78 (m, 2H, Mg−α-CH_2) ppm.

^{13}C{^{1}H} NMR (151 MHz, C_6D_6, 25°C): δ = 162.5 (C_{arom}), 146.9 (C_{arom}), 141.1 (C_{arom}), 138.2 (C_{arom}), 137.4 (C_{arom}), 136.8 (C_{arom}), 136.6 (C_{arom}), 136.6 (C_{arom}), 133.1 (C_{arom}), 133.1 (C_{arom}), 133.0 (C_{arom}), 128.9 (C_{arom}), 119.8 (C_{arom}), 32.0 (C_{aliph}), 30.7 (C_{aliph}), 21.2 (C_{aliph}), 21.2 (C_{aliph}), 20.8 (C_{aliph}), 20.2 (C_{aliph}), 14.4 (C_{aliph}), 6.1 (C_{aliph}) ppm.

Elemental analysis: Calculated values for $C_{40}H_{46}N_2Mg$ (578.35 g/mol): C 82.96, H 8.01, N 4.84; Found: C 81.81, H 7.86, N 4.57.

Synthesis of (^{Mes}DPM)MgNC(THF)$_2$ (4)

A 50 mL Schlenk flask was charged with (^{Mes}DPM)Mg(*n*Bu) (**3**) (520 mg, 0.899 mmol) and dissolved in toluene (12 mL). After cooling the red solution to -70 °C a solution of precooled trimethylsilyl-cyanide (118 µL, 0.943 mmol, 1.05 equiv.) in toluene (10 mL) was added slowly, resulting in a color change from red to orange. The reaction mixture was kept under inert atmosphere and was warmed to room temperature overnight. Removal of solvent in vacuo and trituration with *n*-hexane (3x10 mL) gave the crude product as orange solid. Recrystallization in a mixture of toluene/THF (10:1) at -20 °C yielded crops of crystalline orange blocks. Yield: 346 mg (0.500 mmol; 56 %).

^{1}H NMR (600 MHz, C_6D_6, 25 °C): δ = 6.86 (s, 2H, Mes-aryl-C*H*), 6.80 (s, 4H, Mes-aryl-C*H*), 6.69 (d, *J* = 3.9 Hz, 2H, pyrrole-C*H*), 6.13 (d, *J* = 3.9 Hz, 2H, pyrrole-C*H*), 3.54 – 3.56 (m, 4H, THF^{complex}-C*H*$_2$), 3.52 (br, THF^{free}-C*H*$_2$), 2.27 (s, 6H, Mes-C*H*$_3$), 2.25 (s, 3H, Mes-C*H*$_3$), 2.15 (s, 12H, Mes-C*H*$_3$), 2.13 (s, 6H, Mes-C*H*$_3$), 1.44 – 1.47 (m, 4H, THF-C*H*$_2$), 1.41 (br, THF^{free}-C*H*$_2$) ppm.

^{13}C{^{1}H} NMR (151 MHz, C_6D_6, 25°C): δ = 162.1 (C_{arom}), 146.9 (C_{arom}), 141.2 (C_{arom}), 137.8 (C_{arom}), 137.3 (C_{arom}), 137.3 (C_{arom}), 137.2 (C_{arom}), 136.9 (C_{arom}), 136.9 (C_{arom}), 133.6 (C_{arom}), 132.9 (C_{arom}), 129.0 (C_{arom}), 119.3 (C_{arom}), 67.8 ($C_{THF}{}^{free}$), 67.0 ($C_{THF}{}^{complex}$), 25.9 ($C_{THF}{}^{free}$), 24.8 ($C_{THF}{}^{complex}$), 21.2 (C_{aliph}), 21.2 (C_{aliph}), 20.9 (C_{aliph}), 20.2 (C_{aliph}) ppm.

Signal for Mg–N≡*C* was not observed. See corresponding ^{13}C-labeled NMR spectra.

^{1}H NMR (600 MHz, THF-*d8*, 25 °C): δ = 6.94 (s, 2H, Mes-aryl-C*H*), 6.79 (s, 4H, Mes-aryl-C*H*), 6.47 (d, *J* = 4.0 Hz, 2H, pyrrole-C*H*), 6.12 (d, *J* = 3.9 Hz, 2H, pyrrole-C*H*), 3.60 – 3.56 (m, 4H, THF^{complex}-C*H*$_2$), 3.54 (br, THF^{free}-C*H*$_2$), 2.33 (s, 3H, Mes-C*H*$_3$), 2.20 (s, 6H, Mes-C*H*$_3$), 2.14 (s, 6H, Mes-C*H*$_3$), 2.02 (s, 12H, Mes-C*H*$_3$), 1.79 – 1.71 (m, 4H, THF-C*H*$_2$), 1.69 (br, THF^{free}-C*H*$_2$) ppm.

^{13}C{^{1}H} NMR (151 MHz, C_6D_6, 25°C): δ = 162.5 (C_{arom}), 147.2 (C_{arom}), 141.7 (C_{arom}), 138.4 (C_{arom}), 138.2 (C_{arom}), 138.0 (C_{arom}), 137.6 (C_{arom}), 137.5 (C_{arom}), 134.4 (C_{arom}), 132.8 (C_{arom}), 128.9 (C_{arom}), 128.6 (C_{arom}), 120.0 (C_{arom}), 68.4 ($C_{THF}{}^{complex}$), 67.6 ($C_{THF}{}^{free}$), 26.6 ($C_{THF}{}^{complex}$), 25.5 ($C_{THF}{}^{free}$), 21.5 (C_{aliph}), 21.4 (C_{aliph}), 21.0 (C_{aliph}), 20.4 ppm.

Signal for Mg–N≡*C* was not observed. See corresponding ^{13}C-labeled NMR spectra.

Elemental analysis: Calculated values for $C_{45}H_{53}N_3O_2Mg$ (691.40 g/mol): C 78.08, H 7.72, N 6.07; Found: C 78.41, H 7.25, N 6.65.

2) Crystal structure data

Crystal structure of (^{Mes}DPM)MgN(SiMe$_3$)$_2$ (1) – hasj181114a

An orange crystal of (^{Mes}DPM)MgN(SiMe$_3$)$_2$ (**1**) was embedded in inert perfluoropolyalkylether (viscosity 1800 cSt; ABCR GmbH) and mounted using a Hampton Research CryoLoop. The crystal was then flash cooled to 100 K in a nitrogen gas stream and kept at this temperature during the experiment. The crystal structure was measured on a SuperNova diffractometer with Atlas S2 detector using a CuKα microfocus source. The measured data was processed with the CrysAlisPro software package.[4] Using Olex2[5], the structure was solved with the ShelXT[6] structure solution program using Intrinsic Phasing and refined with the ShelXL[7] refinement package using Least Squares minimization. All hydrogen atoms were placed in ideal positions and refined as riding atoms with relative isotropic displacement parameters.

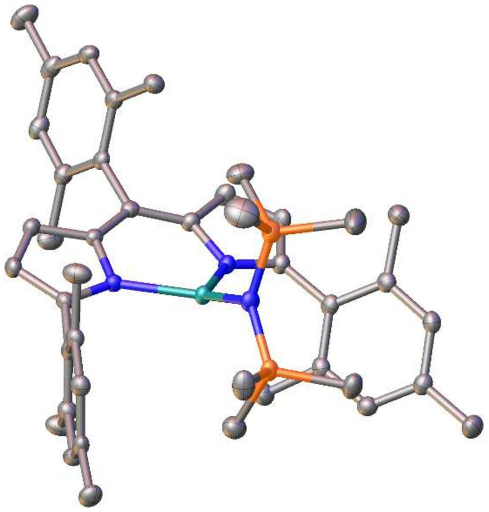

Figure S1. ORTEP plot of (^{Mes}DPM)MgN(SiMe$_3$)$_2$ (**1**) (50% probability).

**Table S1.** Crystal data and structure refinement for (^{Mes}DPM)MgN(SiMe$_3$)$_2$ (**1**)	
Identification code	hasj181114a
Empirical formula	$C_{42}H_{55}MgN_3Si_2$
Formula weight	682.38
Temperature/K	100
Crystal system	Monoclinic
Space group	$P2_1/c$
a/Å	12.77500(10)
b/Å	16.06740(10)
c/Å	19.8664(2)
α/°	90
β/°	96.2640(10)
γ/°	90
Volume/Å^3	4053.45(6)
Z	4
ρ_{calc}g/cm^3	1.118
μ/mm^{-1}	1.172
F(000)	1472.0
Crystal size/mm^3	0.565 × 0.346 × 0.187
Crystal color	Orange
Radiation	CuKα (λ = 1.54184)
2Θ range for data collection/°	6.96 to 136.198
Index ranges	-14 ≤ h ≤ 15, -18 ≤ k ≤ 19, -20 ≤ l ≤ 23
Reflections collected	22222
Independent reflections	7402 [R_{int} = 0.0242, R_{sigma} = 0.0236]
Data/restraints/parameters	7402/0/448
Goodness-of-fit on F^2	1.023
Final R indexes [I>=2σ (I)]	R_1 = 0.0365, wR_2 = 0.0952
Final R indexes [all data]	R_1 = 0.0389, wR_2 = 0.0975
Largest diff. peak/hole / e Å^{-3}	0.29/-0.41

Crystal structure of (^{Mes}DPM)Mg(N(SiMe$_3$)$_2$)(NC-TMS) (2) – hasj181016b

An orange crystal of (^{Mes}DPM)Mg(N(SiMe$_3$)$_2$)(NC-TMS) (**2**) was embedded in inert perfluoropolyalkylether (viscosity 1800 cSt; ABCR GmbH) and mounted using a Hampton Research CryoLoop. The crystal was then flash cooled to 100 K in a nitrogen gas stream and kept at this temperature during the experiment. The crystal structure was measured on a SuperNova diffractometer with Atlas S2 detector using a CuKα microfocus source. The measured data was processed with the CrysAlisPro software package.[4] Using Olex2[5], the structure was solved with the ShelXT[6] structure solution program using Intrinsic Phasing and refined with the ShelXL[7] refinement package using Least Squares minimization. All hydrogen atoms were placed in ideal positions and refined as riding atoms with relative isotropic displacement parameters.

The trimethylsilyl-group of the coordinated trimethylsilylcyanide was found to be disordered over two positions. The relative contributions of the two orientations were refined to ~ 0.50/0.50.

A satisfactory disorder model for the solvent could not found, and therefore the OLEX2 Solvent Mask routine (similar to PLATON/SQUEEZE) was used to mask out the disordered electron density (49 e$^-$).[8]

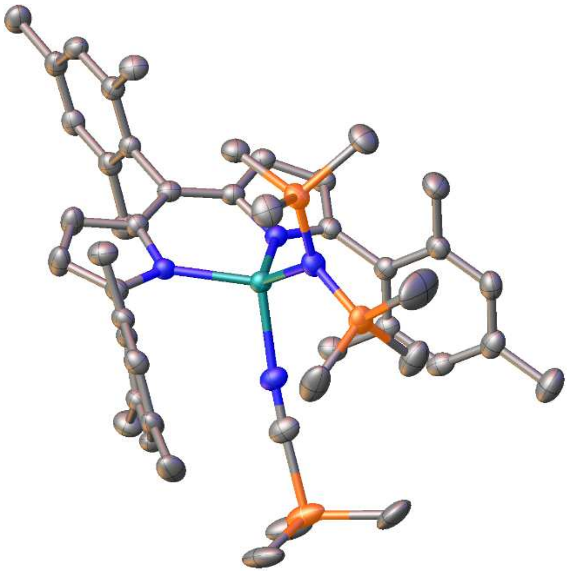

Figure S2. ORTEP plot of (^{Mes}DPM)Mg(N(SiMe$_3$)$_2$)(NC-TMS) (**2**) (50% probability).

**Table S2.** Crystal data and structure refinement for (^{Mes}DPM)Mg(N($SiMe_3$)$_2$)(NC-TMS) (**2**)	
Identification code	hasj181016b
Empirical formula	$C_{46}H_{64}MgN_4Si_3$
Formula weight	781.59
Temperature/K	100
Crystal system	Triclinic
Space group	P-1
a/Å	11.8865(3)
b/Å	12.0067(3)
c/Å	18.0349(3)
α/°	86.850(2)
β/°	82.801(2)
γ/°	75.319(2)
Volume/Å^3	2469.52(10)
Z	2
ρ_{calc}g/cm^3	1.051
μ/mm^{-1}	1.246
F(000)	1592.0
Crystal size/mm^3	0.178 × 0.141 × 0.068
Crystal color	Orange
Radiation	CuKα (λ = 1.54184)
2Θ range for data collection/°	7.614 to 136.212
Index ranges	-14 ≤ h ≤ 14, -14 ≤ k ≤ 14, -21 ≤ l ≤ 21
Reflections collected	50817
Independent reflections	9030 [R_{int} = 0.0435, R_{sigma} = 0.0262]
Data/restraints/parameters	9030/0/536
Goodness-of-fit on F^2	1.034
Final R indexes [I>=2σ (I)]	R_1 = 0.0393, wR_2 = 0.1042
Final R indexes [all data]	R_1 = 0.0454, wR_2 = 0.1091
Largest diff. peak/hole / e Å^{-3}	0.30/-0.35

Crystal structure of (^{Mes}DPM)Mg(*n*Bu) (3) – hasj180109b

A red crystal of (^{Mes}DPM)Mg(*n*Bu) (**3**) was embedded in inert perfluoropolyalkylether (viscosity 1800 cSt; ABCR GmbH) and mounted using a Hampton Research CryoLoop. The crystal was then flash cooled to 100 K in a nitrogen gas stream and kept at this temperature during the experiment. The crystal structure was measured on a SuperNova diffractometer with Atlas S2 detector using a CuKα microfocus source. The measured data was processed with the CrysAlisPro software package.[4] Using Olex2[5], the structure was solved with the ShelXT[6] structure solution program using Intrinsic Phasing and refined with the ShelXL[7] refinement package using Least Squares minimization. All hydrogen atoms were placed in ideal positions and refined as riding atoms with relative isotropic displacement parameters.

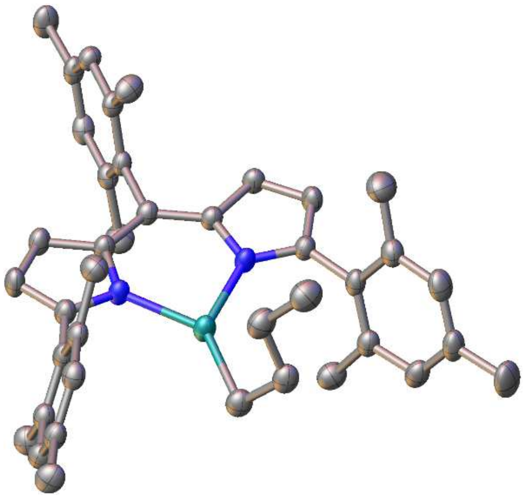

Figure S3. ORTEP plot of (^{Mes}DPM)Mg(*n*Bu) (**3**) (50% probability).

**Table S3.** Crystal data and structure refinement for (^{Mes}DPM)Mg(*n*Bu) (**3**)	
Identification code	hasj180509a
Empirical formula	$C_{40}H_{46}MgN_2$
Formula weight	579.10
Temperature/K	100
Crystal system	Monoclinic
Space group	$P2_1/c$
a/Å	15.5543(4)
b/Å	14.6575(2)
c/Å	15.5206(4)
α/°	90
β/°	107.586(3)
γ/°	90
Volume/Å^3	3373.12(14)
Z	4
ρ_{calc}g/cm^3	1.140
μ/mm^{-1}	0.662
F(000)	1248.0
Crystal size/mm^3	0.236 × 0.131 × 0.1
Crystal color	Red
Radiation	CuKα (λ = 1.54184)
2Θ range for data collection/°	5.96 to 136.226
Index ranges	-18 ≤ h ≤ 15, -17 ≤ k ≤ 17, -18 ≤ l ≤ 18
Reflections collected	18667
Independent reflections	6141 [R_{int} = 0.0248, R_{sigma} = 0.0239]
Data/restraints/parameters	6141/0/398
Goodness-of-fit on F^2	1.034
Final R indexes [I>=2σ (I)]	R_1 = 0.0522, wR_2 = 0.1446
Final R indexes [all data]	R_1 = 0.0602, wR_2 = 0.1534
Largest diff. peak/hole / e Å^{-3}	1.10/-0.39

Crystal structure of (^{Mes}DPM)MgNC(THF)$_2$ (4) – hasj181011a

An orange crystal of (^{Mes}DPM)MgNC(THF)$_2$ (**4**) was embedded in inert perfluoropolyalkylether (viscosity 1800 cSt; ABCR GmbH) and mounted using a Hampton Research CryoLoop. The crystal was then flash cooled to 100 K in a nitrogen gas stream and kept at this temperature during the experiment. The crystal structure was measured on a SuperNova diffractometer with Atlas S2 detector using a CuKα microfocus source. The measured data was processed with the CrysAlisPro software package.[4] Using Olex2[5], the structure was solved with the ShelXT[6] structure solution program using Intrinsic Phasing and refined with the ShelXL[7] refinement package using Least Squares minimization. All hydrogen atoms were placed in ideal positions and refined as riding atoms with relative isotropic displacement parameters.

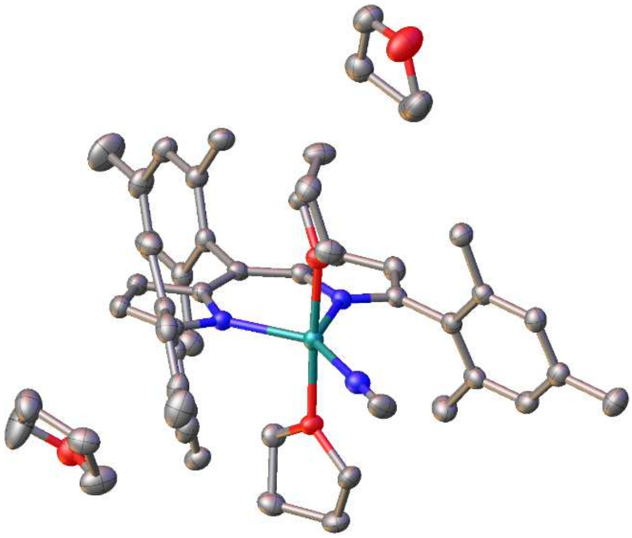

Figure S4. ORTEP plot of (^{Mes}DPM)MgNC(THF)$_2$ (**4**) (50% probability).

**Table S4.** Crystal data and structure refinement for (^{Mes}DPM)MgNC(THF)$_2$ (**4**)	
Identification code	hasj181011a
Empirical formula	$C_{53}H_{69}MgN_3O_4$
Formula weight	836.42
Temperature/K	100
Crystal system	Monoclinic
Space group	$P2_1/n$
a/Å	11.3228(5)
b/Å	23.1796(9)
c/Å	18.7284(8)
α/°	90
β/°	105.867(5)
γ/°	90
Volume/Å^3	4728.1(4)
Z	4
ρ_{calc}g/cm^3	1.175
μ/mm^{-1}	0.690
F(000)	1808.0
Crystal size/mm^3	0.45 × 0.271 × 0.259
Crystal color	Orange
Radiation	CuKα (λ = 1.54184)
2Θ range for data collection/°	6.214 to 136.216
Index ranges	-13 ≤ h ≤ 9, -27 ≤ k ≤ 26, -22 ≤ l ≤ 22
Reflections collected	25636
Independent reflections	8585 [R_{int} = 0.0568, R_{sigma} = 0.0498]
Data/restraints/parameters	8585/0/559
Goodness-of-fit on F^2	1.026
Final R indexes [I>=2σ (I)]	R_1 = 0.0682, wR_2 = 0.1818
Final R indexes [all data]	R_1 = 0.0841, wR_2 = 0.1965
Largest diff. peak/hole / e Å^{-3}	0.62/-0.48

3) Selected ^{1}H, ^{13}C, DEPT-135, ATP, COSY, HSQC, HMBC and DOSY NMR spectra

Figure S5. ^{1}H, ^{13}C NMR spectra of (^{Mes}DPM)MgN($SiMe_3$)$_2$ (**1**) in benzene-*d*$_6$ (*).

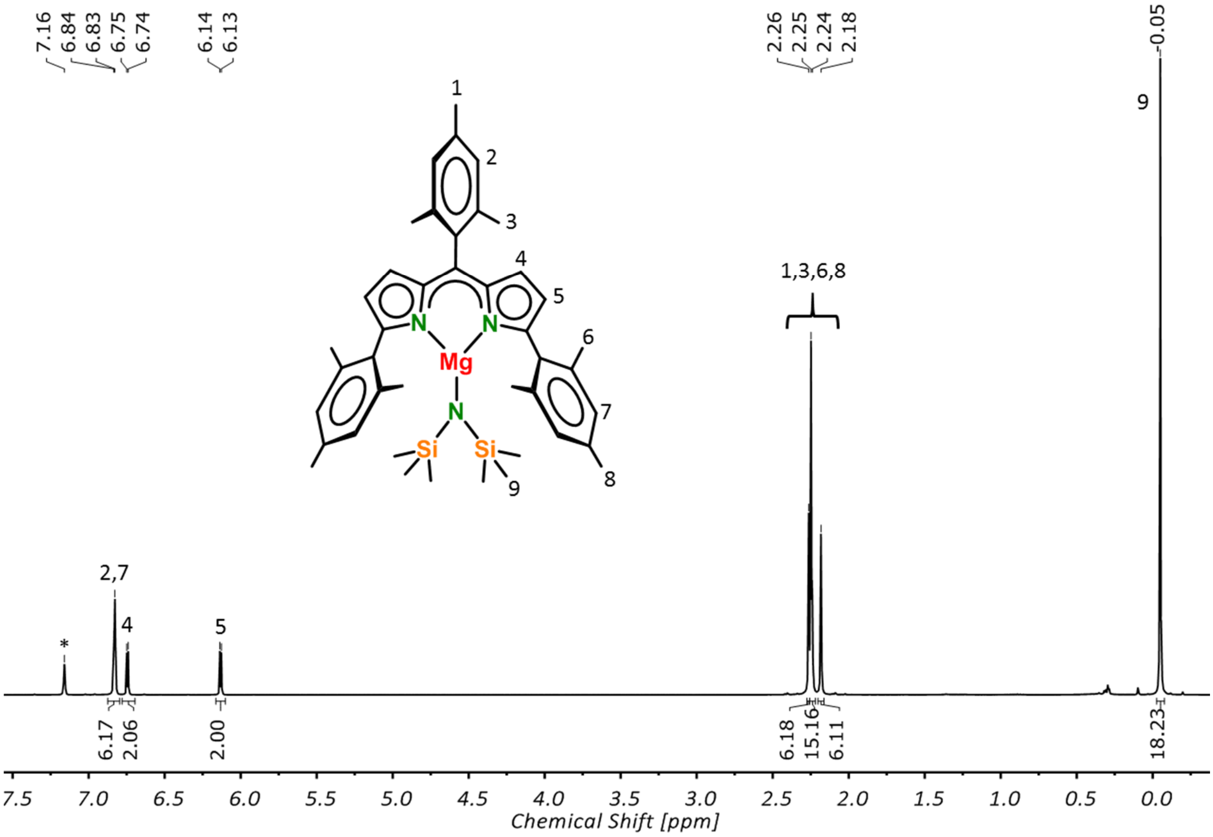

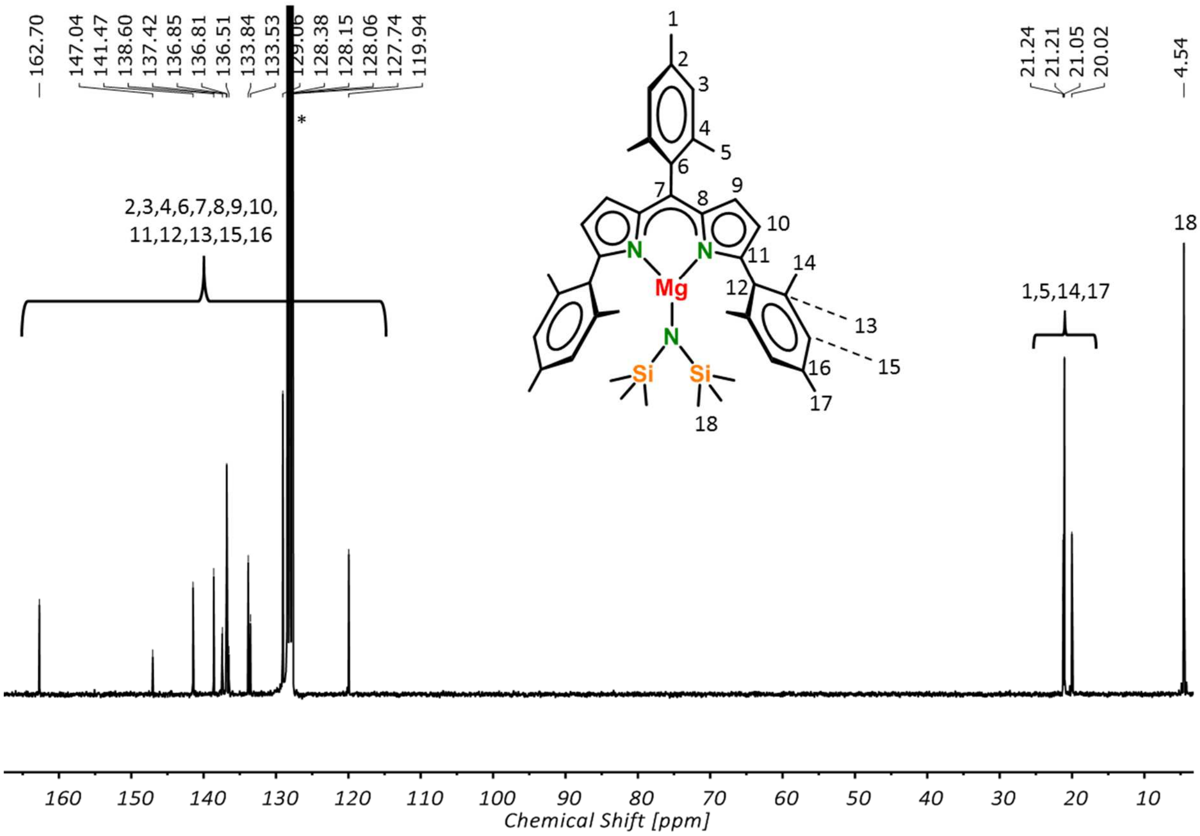

Figure S6. ^{1}H, ^{13}C, COSY, HSQC and HMBC spectra of (^{Mes}DPM)Mg(N(SiMe$_3$)$_2$)(NC-TMS) (**2**) in benzene-d_6 (*).

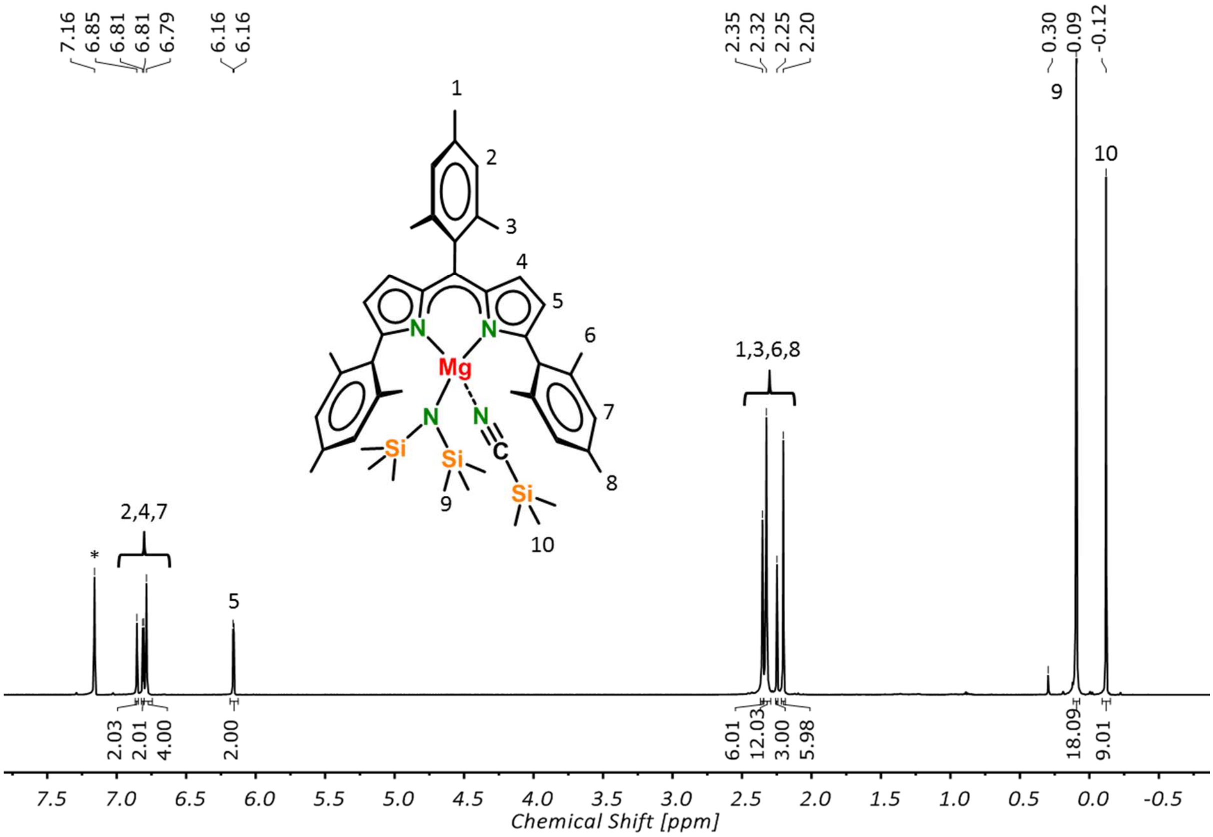

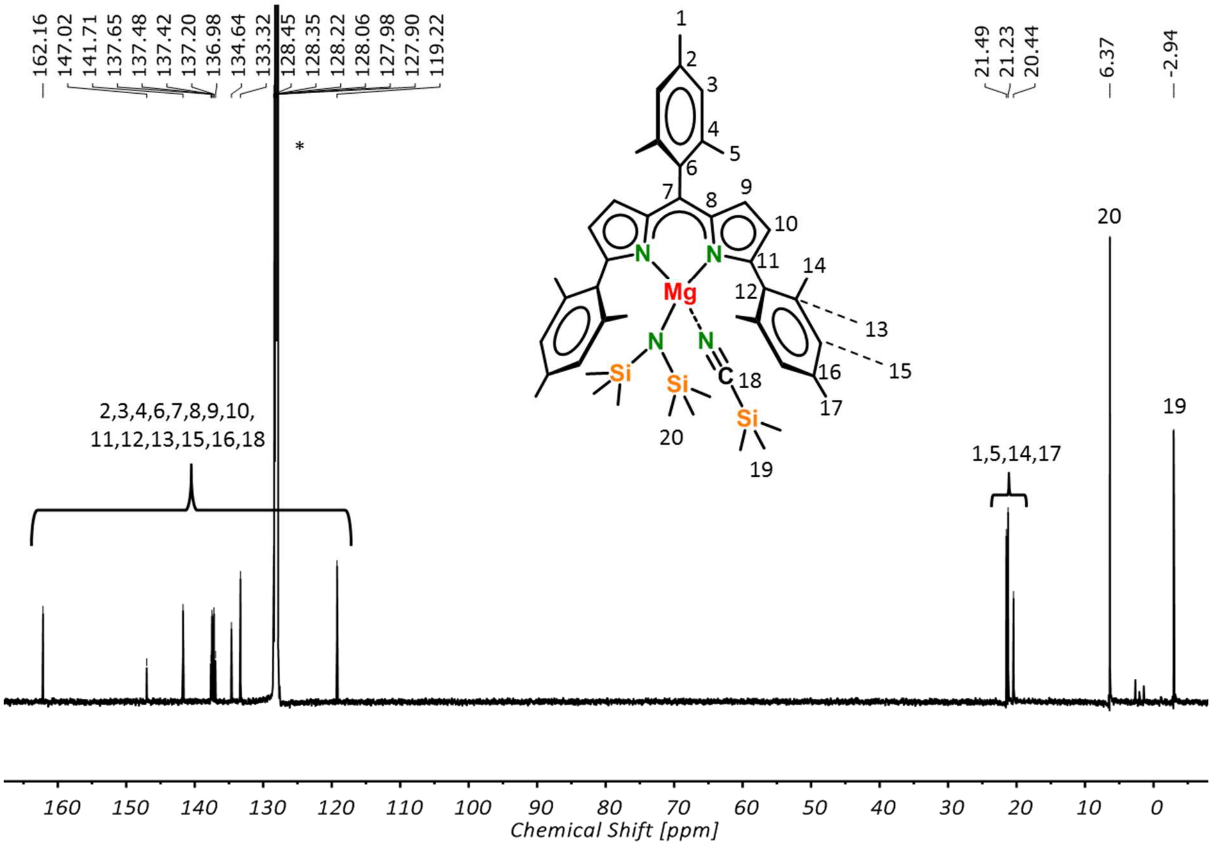

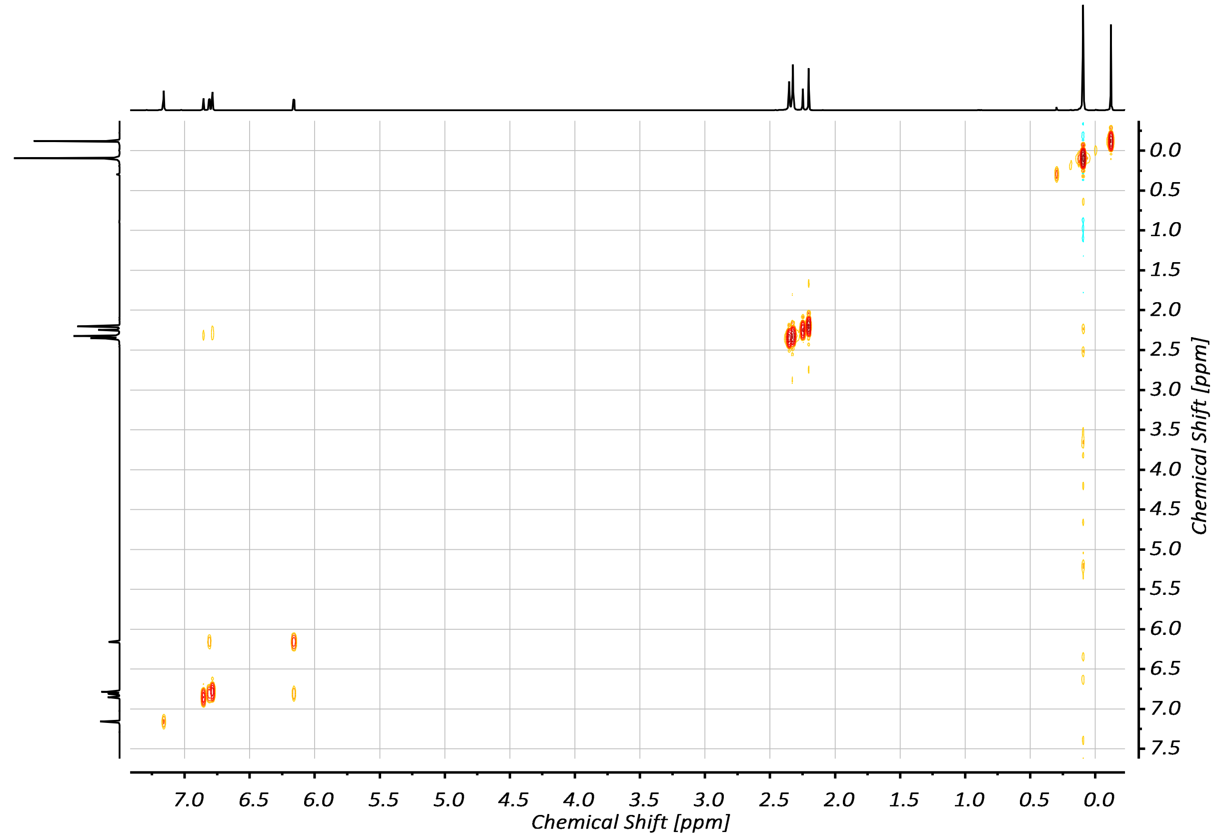

Chemical Shift [ppm]
Chemical Shift [ppm]

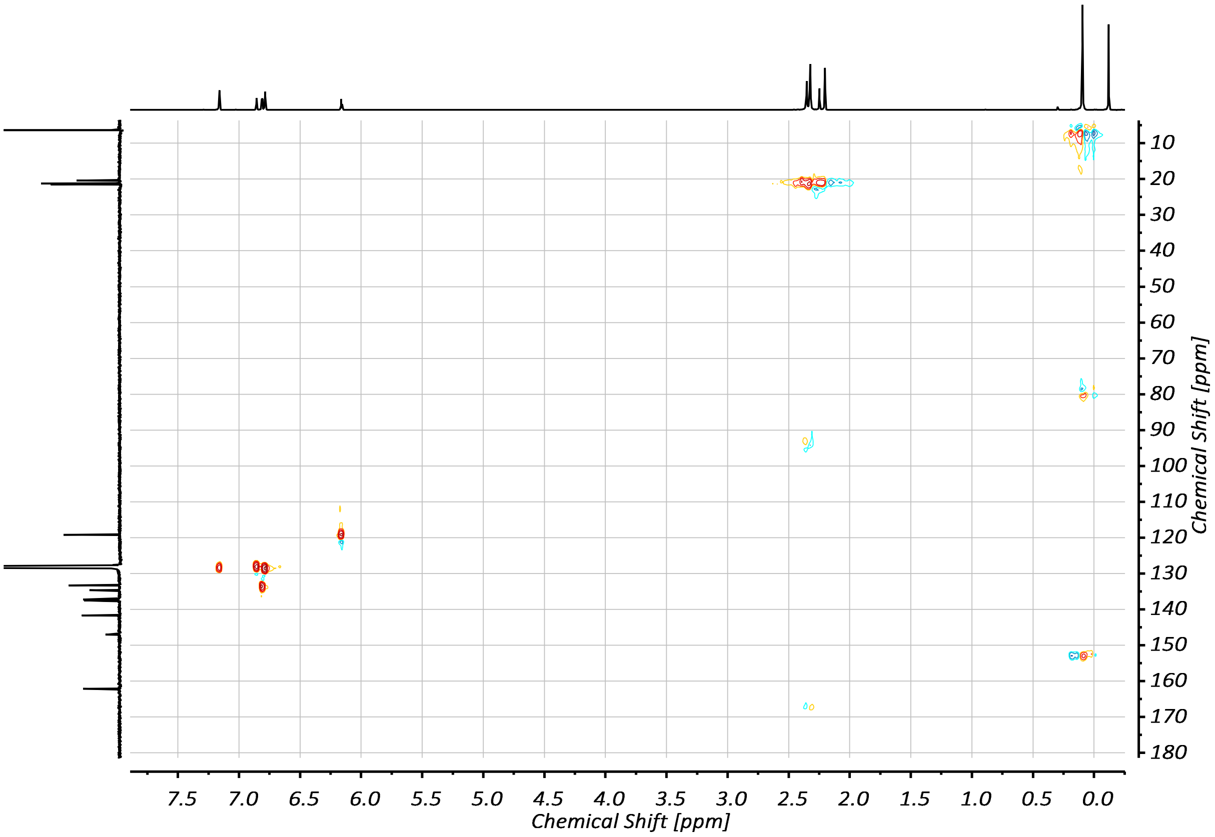

Chemical Shift [ppm]
Chemical Shift [ppm]

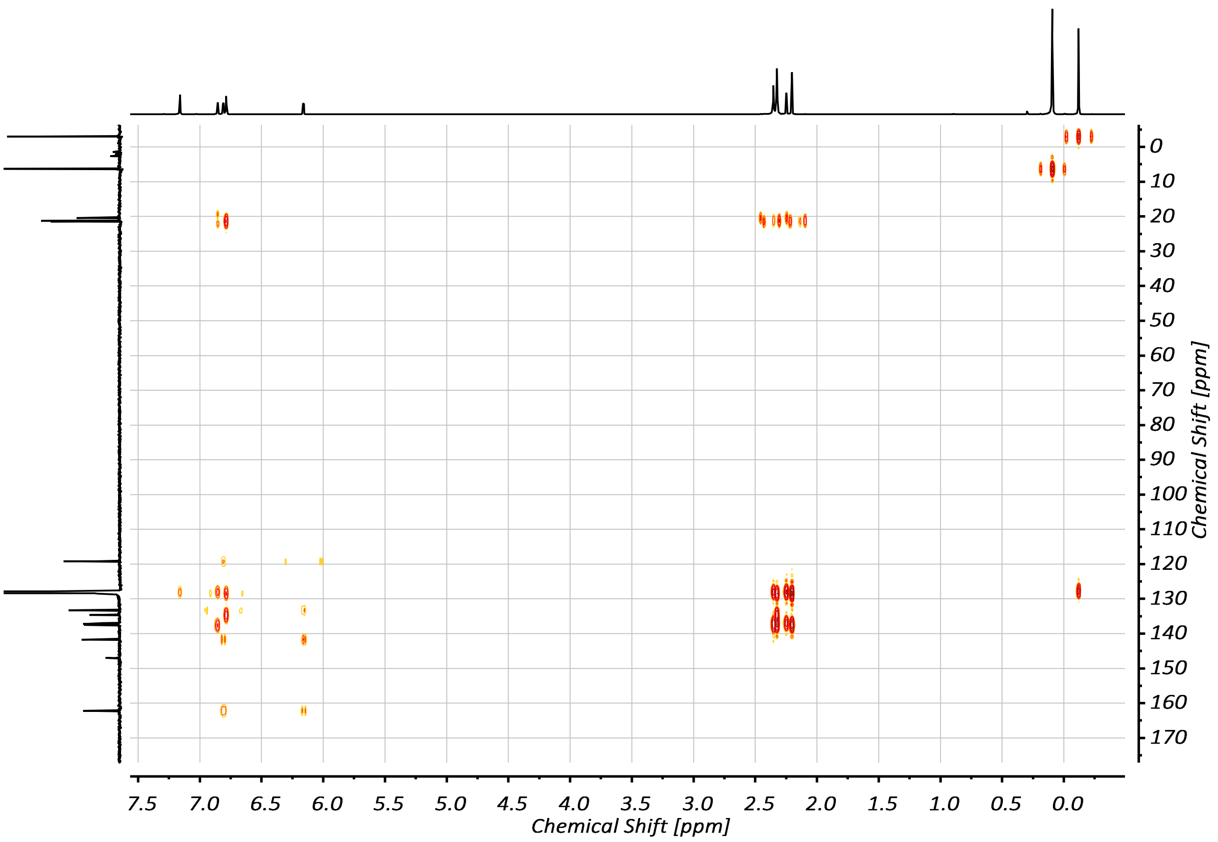

Figure S7. ^{1}H, ^{13}C, COSY, HSQC and HMBC spectra of (^{Mes}DPM)Mg(*n*Bu) (**3**) in benzene-d_6 (*).

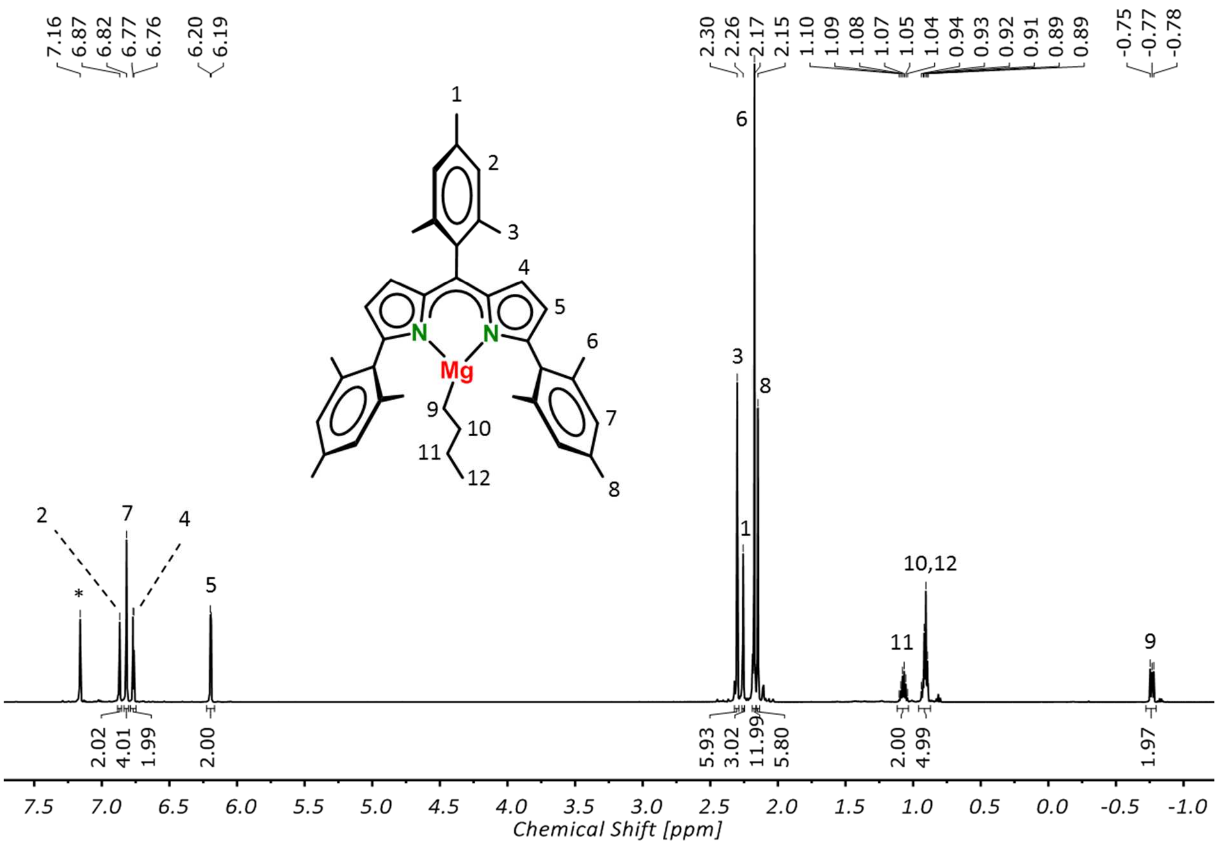

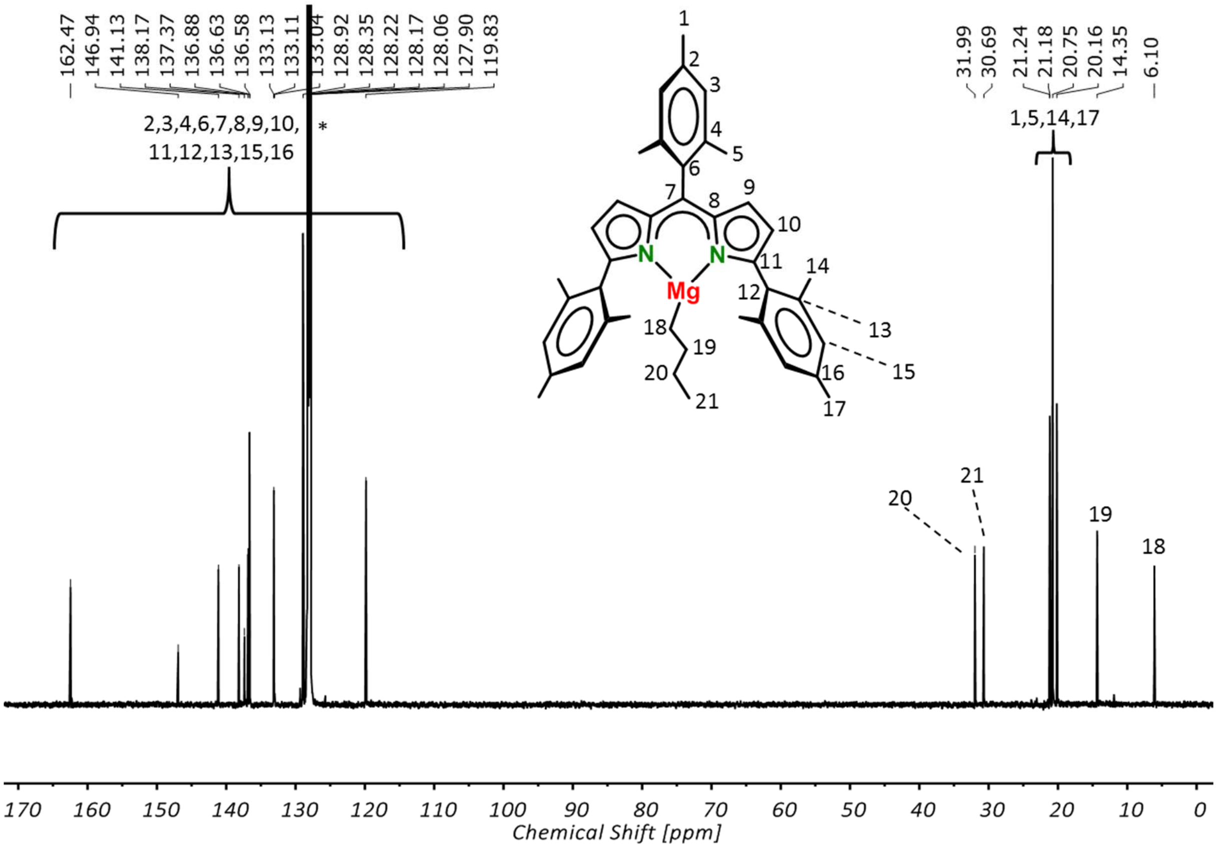
162.47
146.94
141.13
138.17
137.37
136.88
136.63
136.58
133.13
133.11
133.04
128.92
128.35
128.22
128.17
128.06
127.90
119.83
31.99
30.69
21.24
21.18
20.75
20.16
14.35
6.10
2,3,4,6,7,8,9,10,
11,12,13,15,16
*
1,5,14,17
N
N
Mg
20
21
19
18
170 160 150 140 130 120 110 100 90 80 70 60 50 40 30 20 10 0
Chemical Shift [ppm]

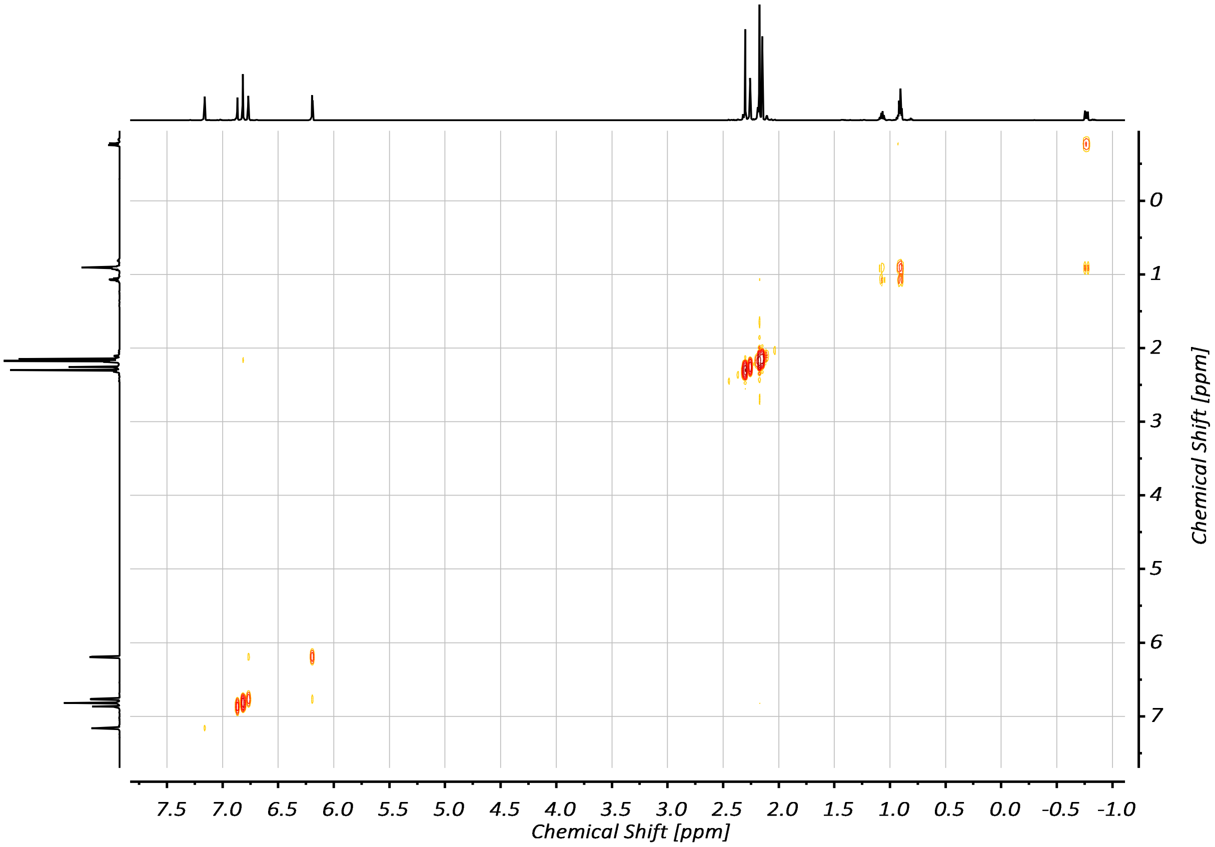
7.5 7.0 6.5 6.0 5.5 5.0 4.5 4.0 3.5 3.0 2.5 2.0 1.5 1.0 0.5 0.0 -0.5 -1.0
Chemical Shift [ppm]
0 1 2 3 4 5 6 7
Chemical Shift [ppm]

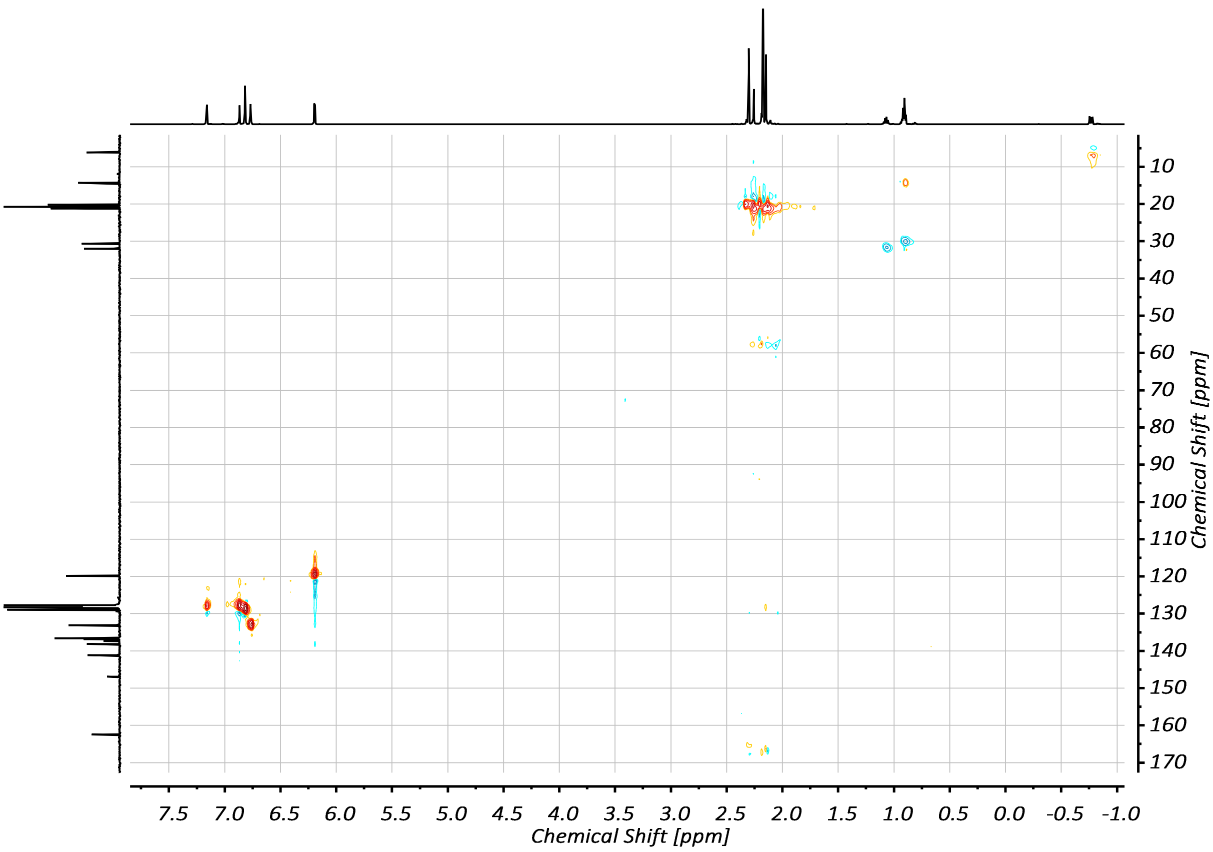
Chemical Shift [ppm]
Chemical Shift [ppm]

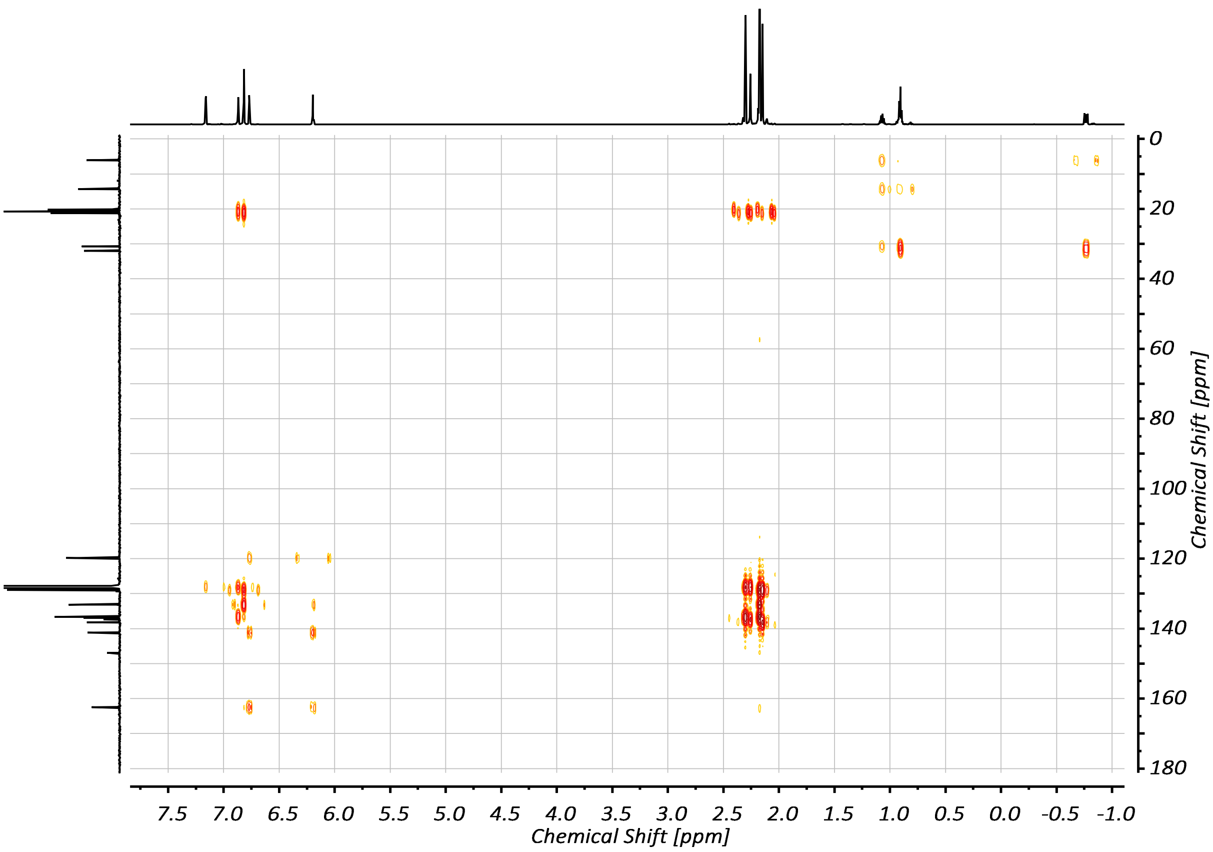
Chemical Shift [ppm]
Chemical Shift [ppm]

Figure S8. ^{1}H, ^{13}C, COSY, HSQC and HMBC spectra of (^{Mes}DPM)MgNC(THF)$_2$ (**4**) in benzene-d_6 (*).

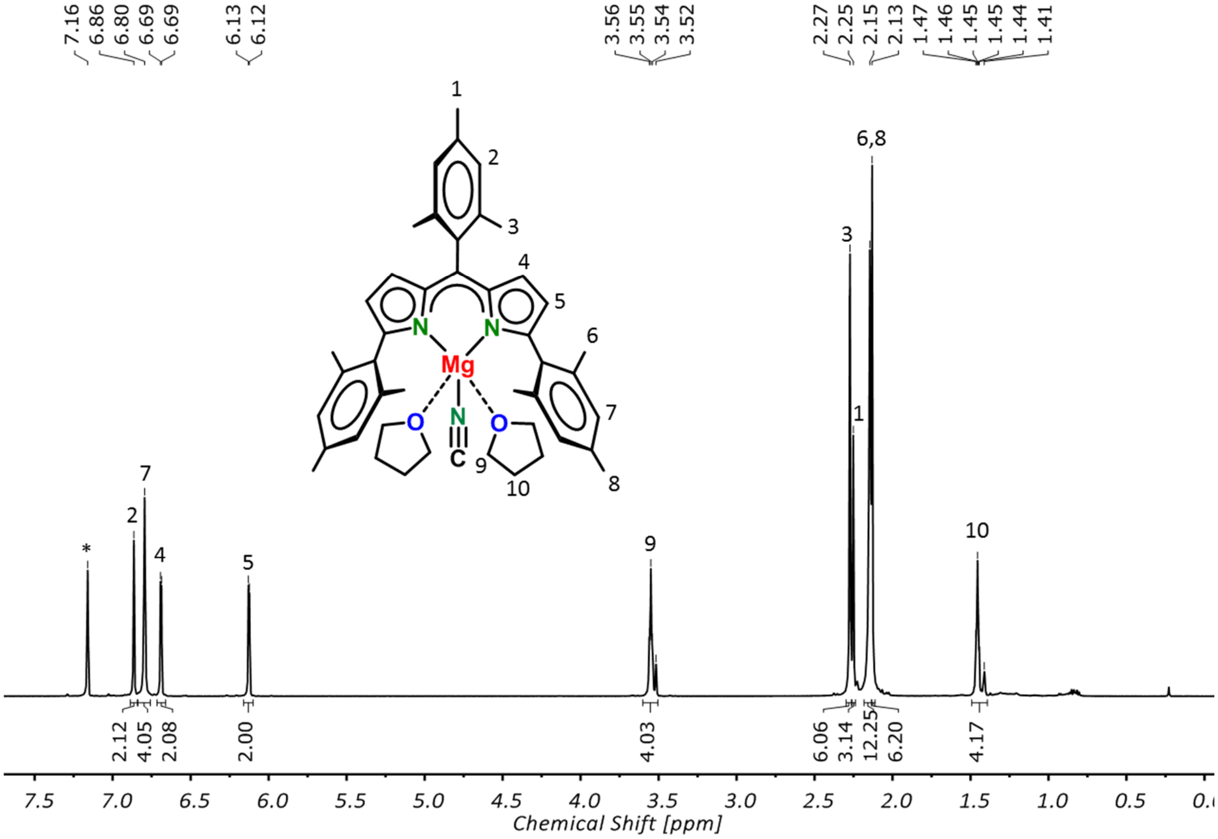

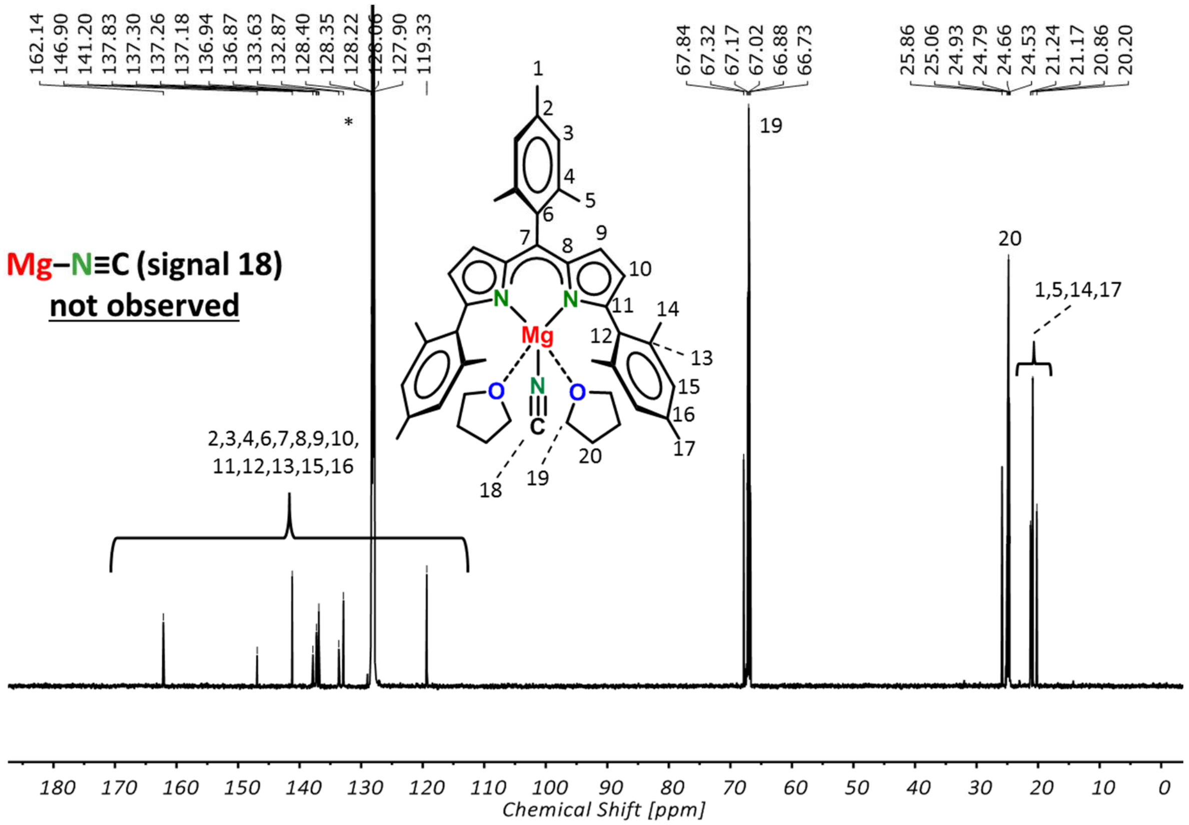

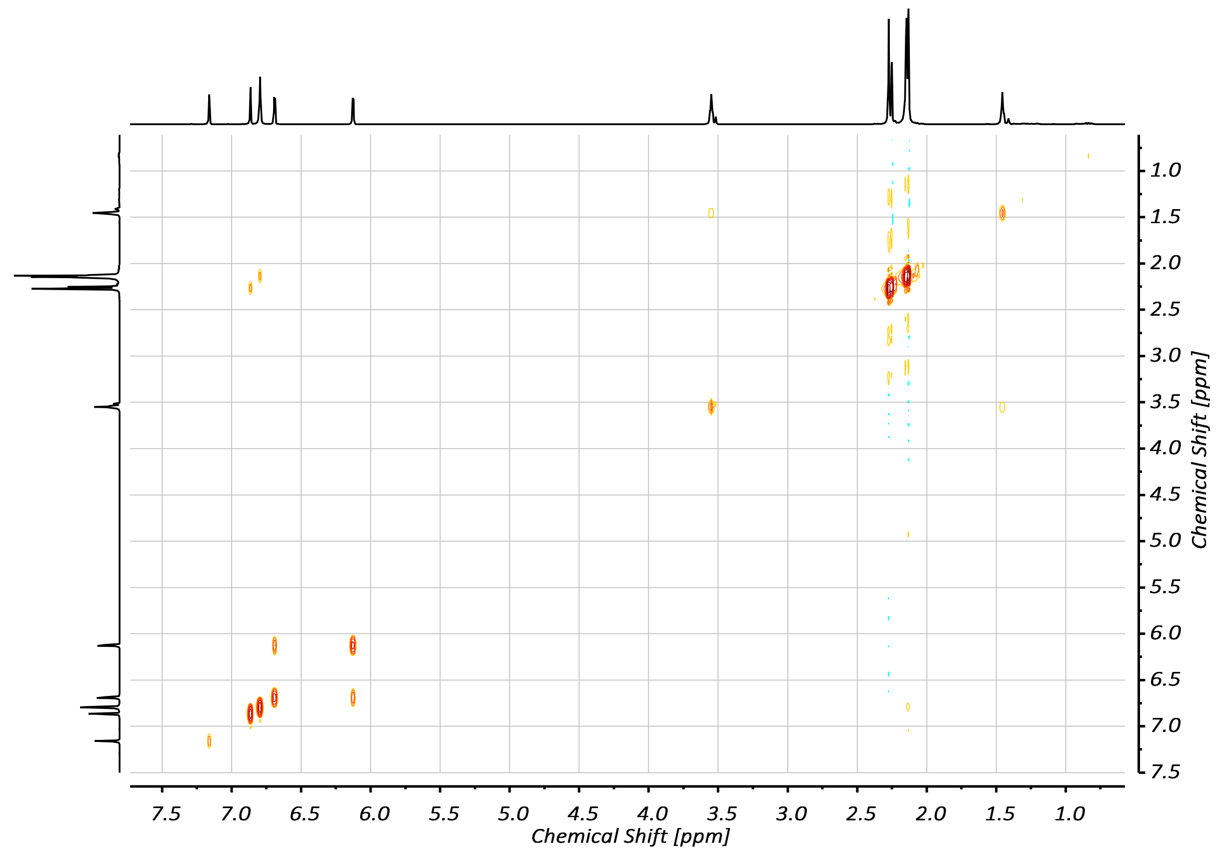

7.5
7.0
6.5
6.0
5.5
5.0
4.5
4.0
3.5
3.0
2.5
2.0
1.5
1.0
Chemical Shift [ppm]
Chemical Shift [ppm]

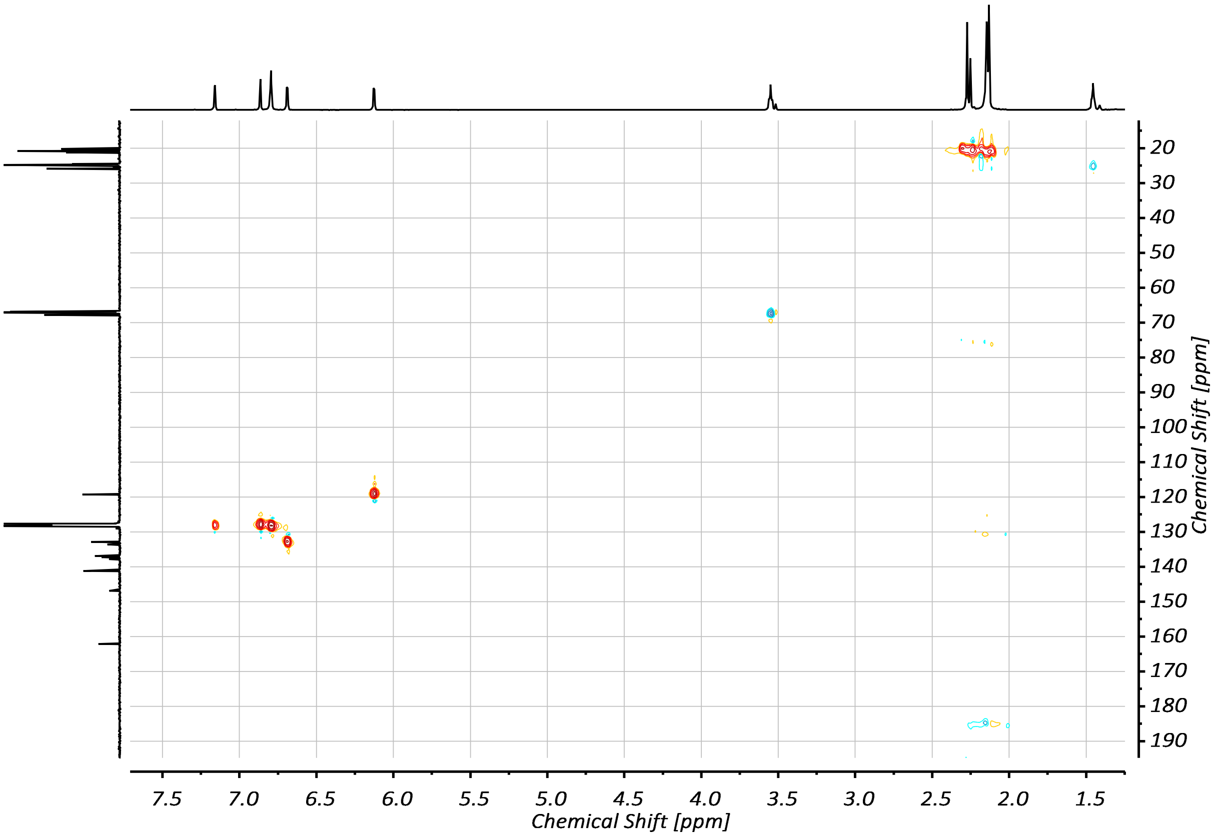

20
30
40
50
60
70
80
90
100
110
120
130
140
150
160
170
180
190
7.5
7.0
6.5
6.0
5.5
5.0
4.5
4.0
3.5
3.0
2.5
2.0
1.5
Chemical Shift [ppm]
Chemical Shift [ppm]

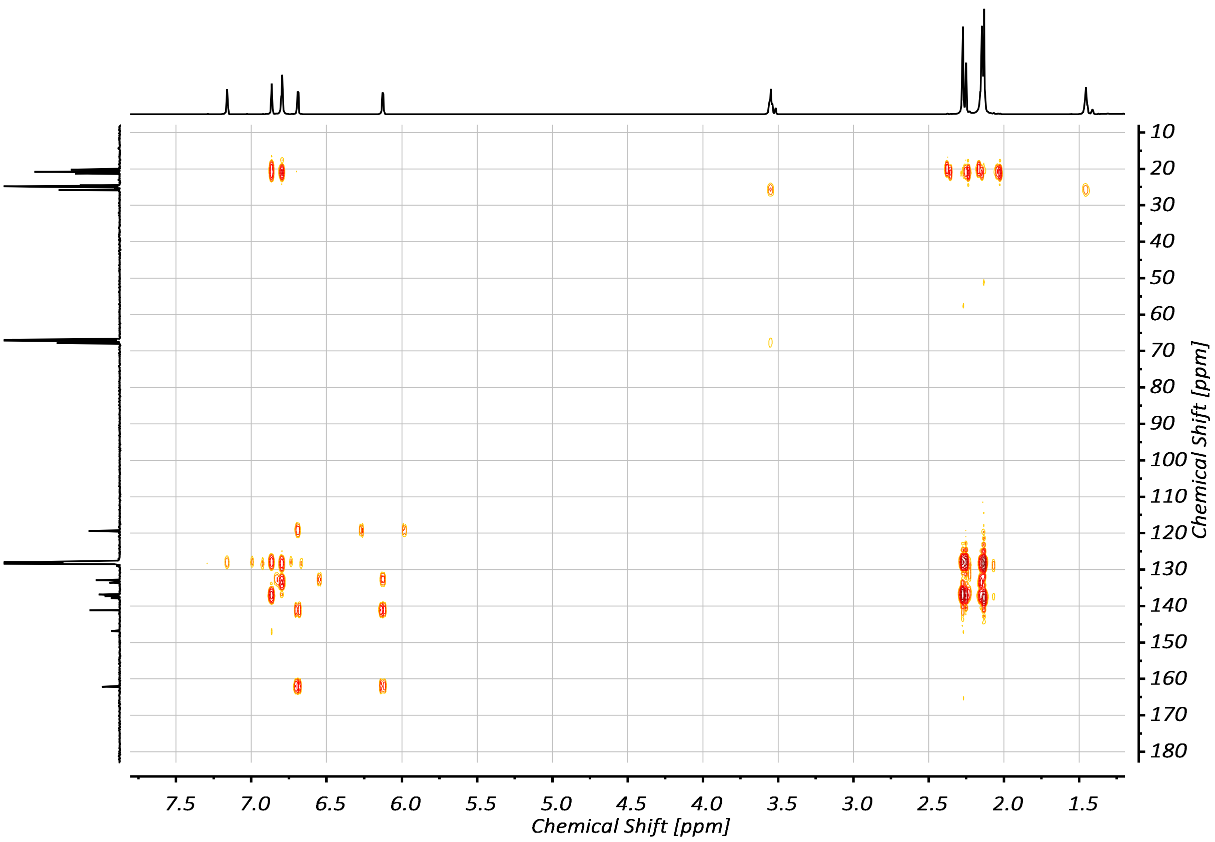

Figure S9. ^{1}H, ^{13}C, DEPT-135, ATP, COSY, HSQC and HMBC spectra of (^{Mes}DPM)MgNC(THF)$_2$ (**4**) in THF-d_8 (*).

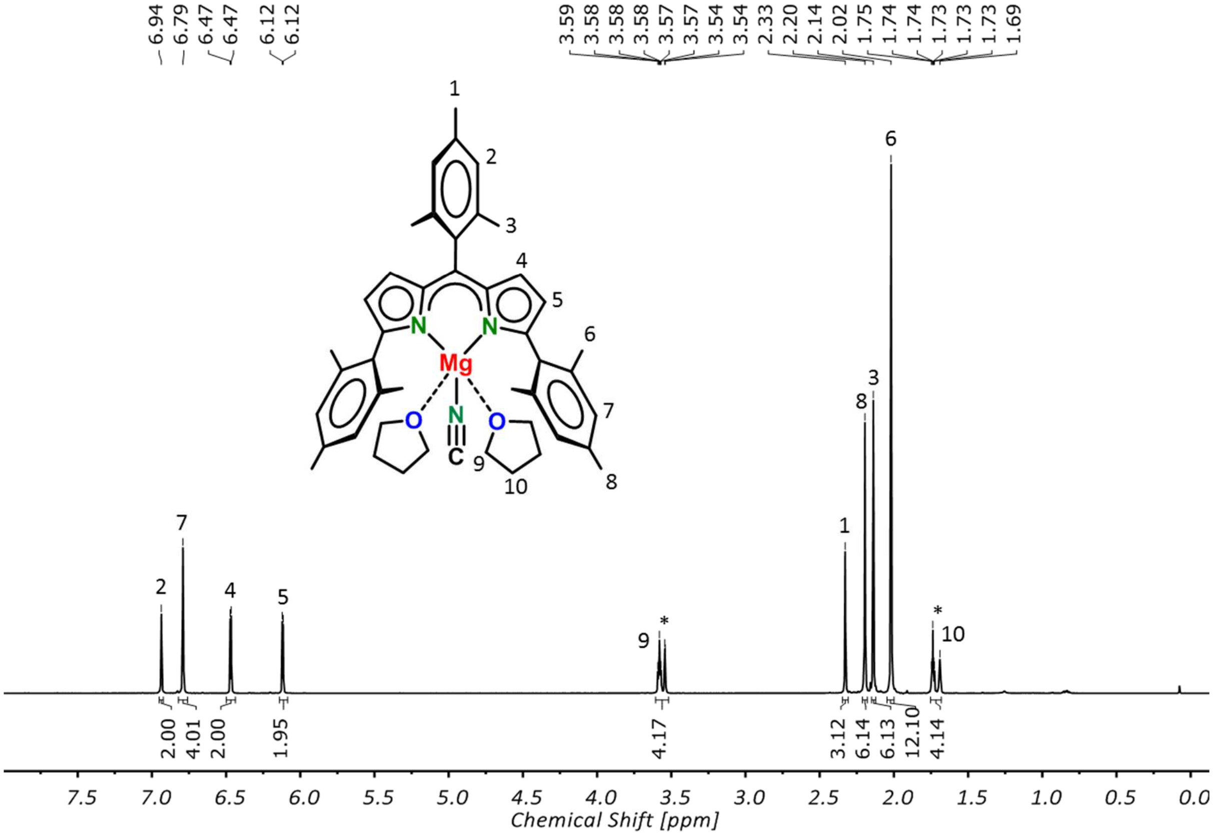

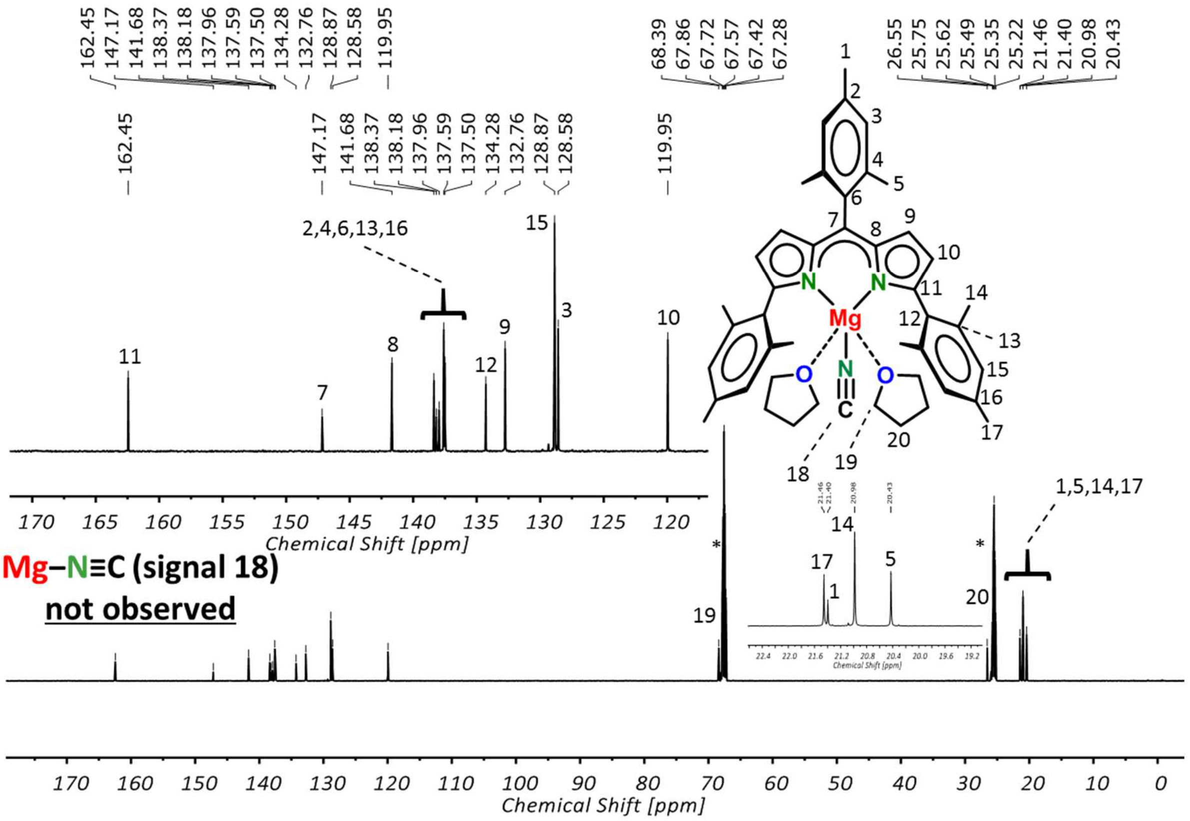

162.45
147.17
141.68
138.37
138.18
137.96
137.59
137.50
134.28
132.76
128.87
128.58
119.95
68.39
67.86
67.72
67.57
67.42
67.28
26.55
25.75
25.62
25.49
25.35
25.22
21.46
21.40
20.98
20.43
2,4,6,13,16
1,5,14,17
Mg
N
O
C
Mg–N≡C (signal 18)
not observed
Chemical Shift [ppm]

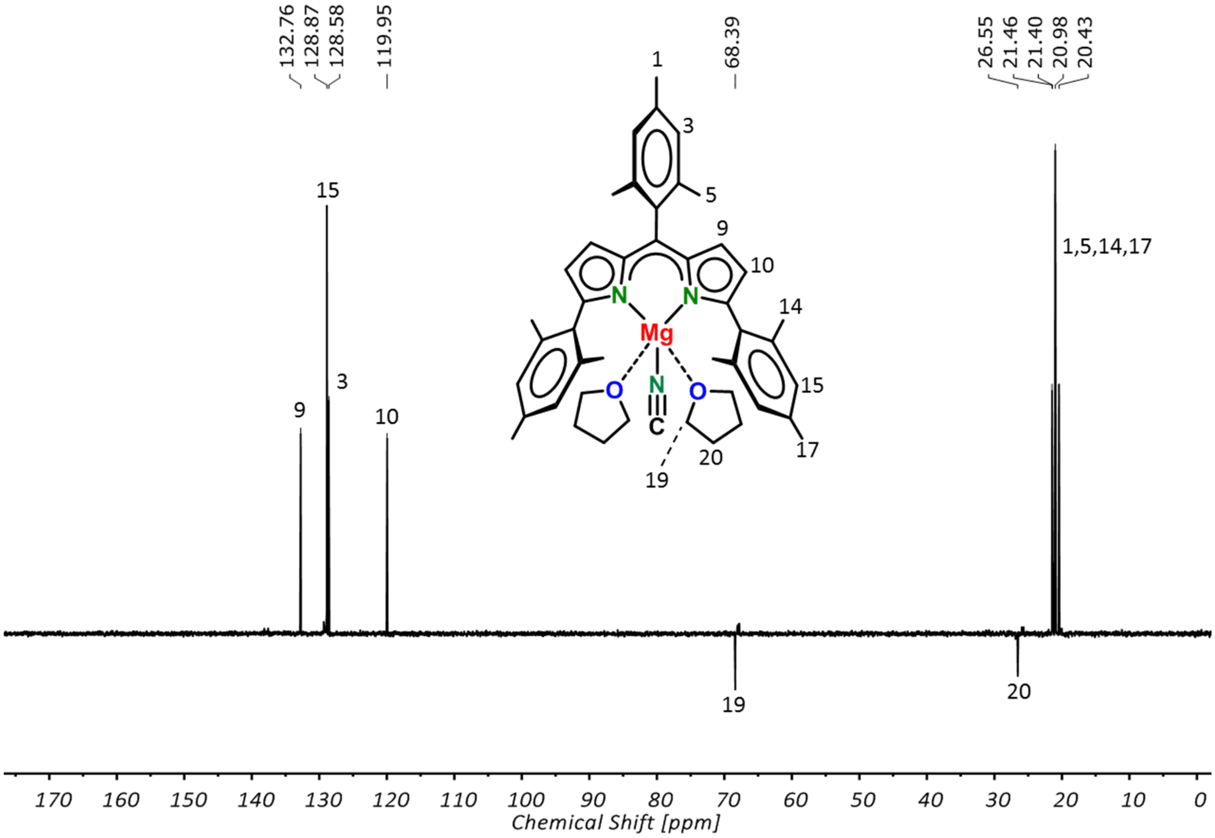

132.76
128.87
128.58
119.95
68.39
26.55
21.46
21.40
20.98
20.43
1,5,14,17
Mg
N
O
C
Chemical Shift [ppm]

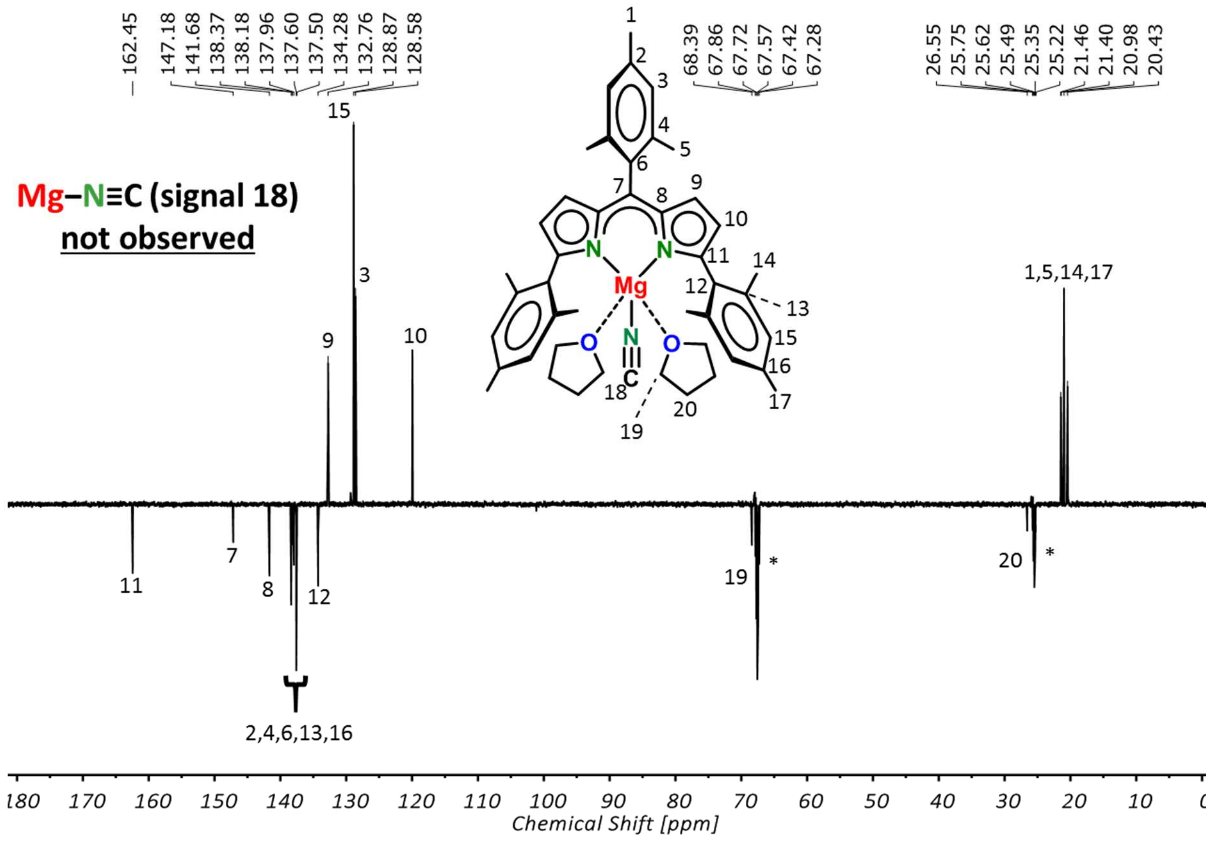
162.45
147.18
141.68
138.37
138.18
137.96
137.60
137.50
134.28
132.76
128.87
128.58
68.39
67.86
67.72
67.57
67.42
67.28
26.55
25.75
25.62
25.49
25.35
25.22
21.46
21.40
20.98
20.43
Mg–N≡C (signal 18)
not observed
1,5,14,17
2,4,6,13,16
Chemical Shift [ppm]

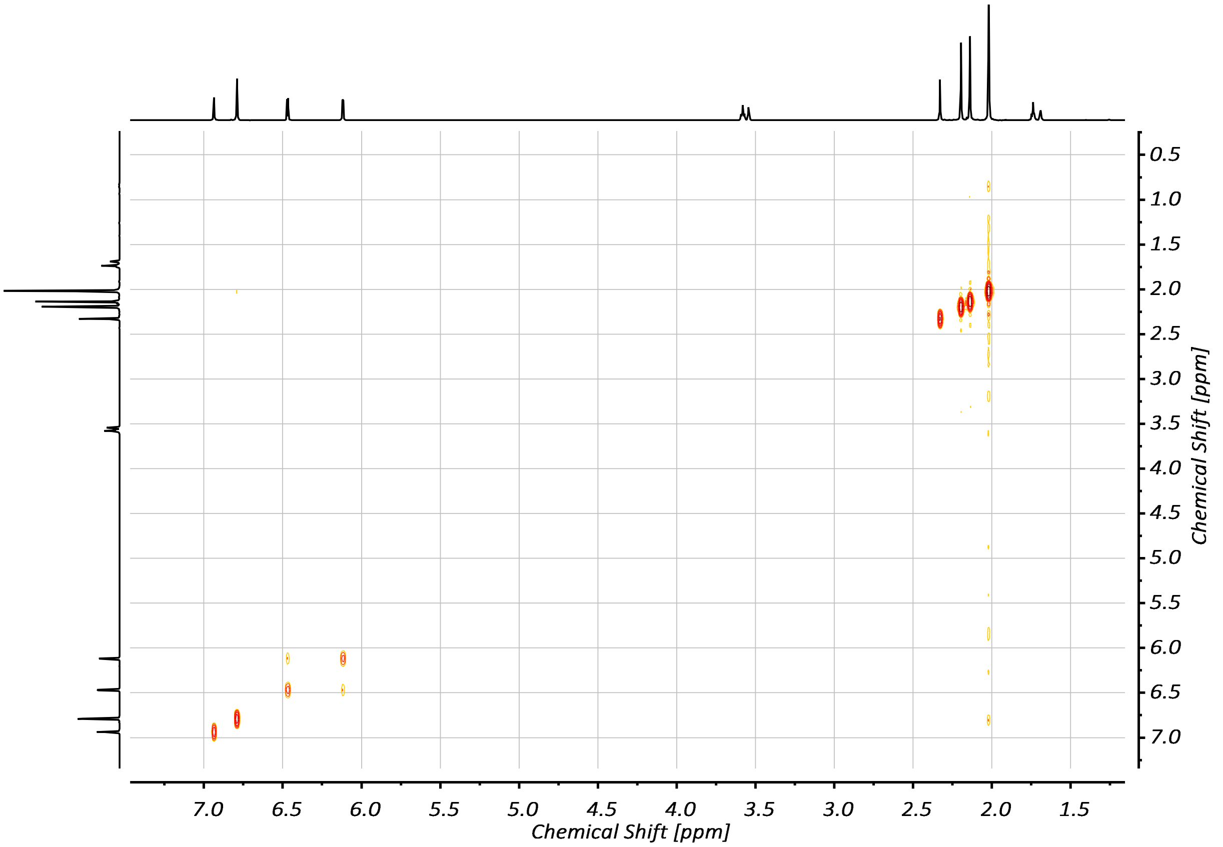
Chemical Shift [ppm]
Chemical Shift [ppm]

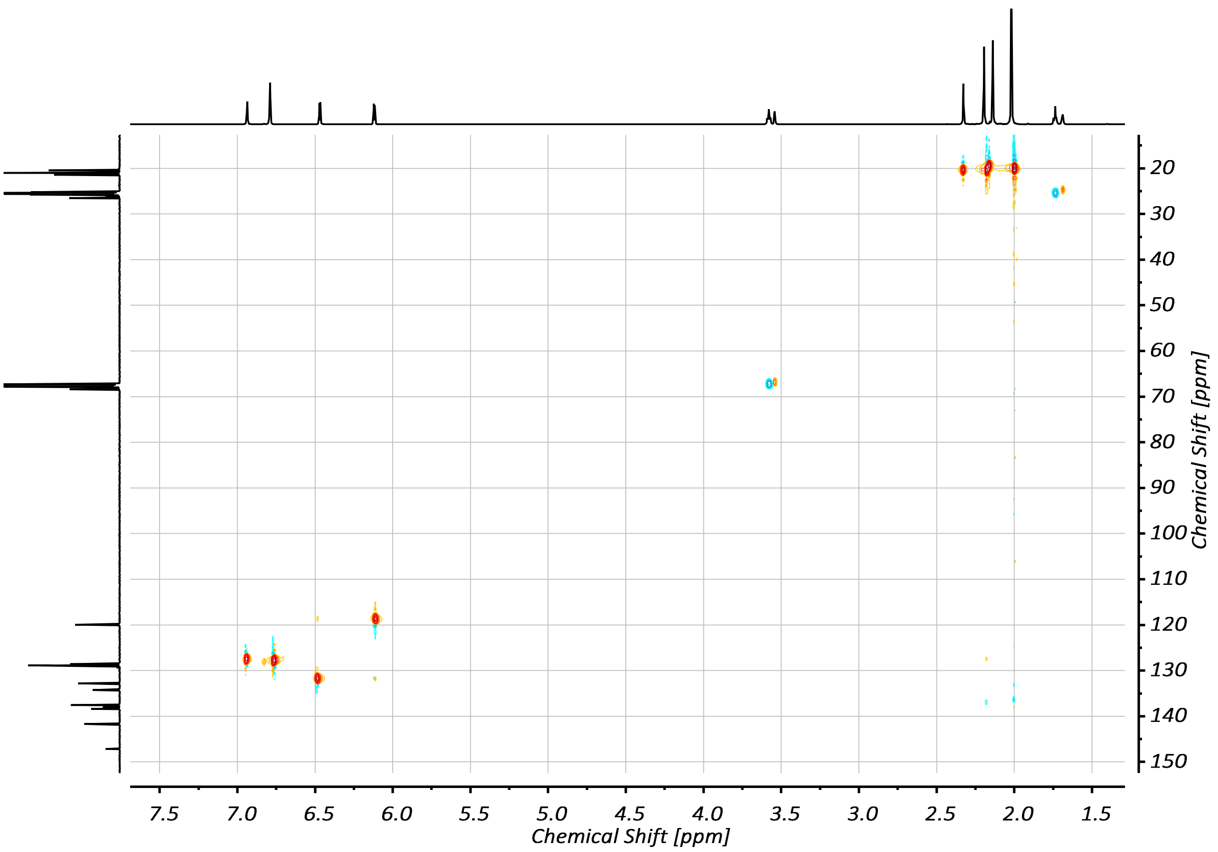

7.5
7.0
6.5
6.0
5.5
5.0
4.5
4.0
3.5
3.0
2.5
2.0
1.5
Chemical Shift [ppm]
20
30
40
50
60
70
80
90
100
110
120
130
140
150
Chemical Shift [ppm]

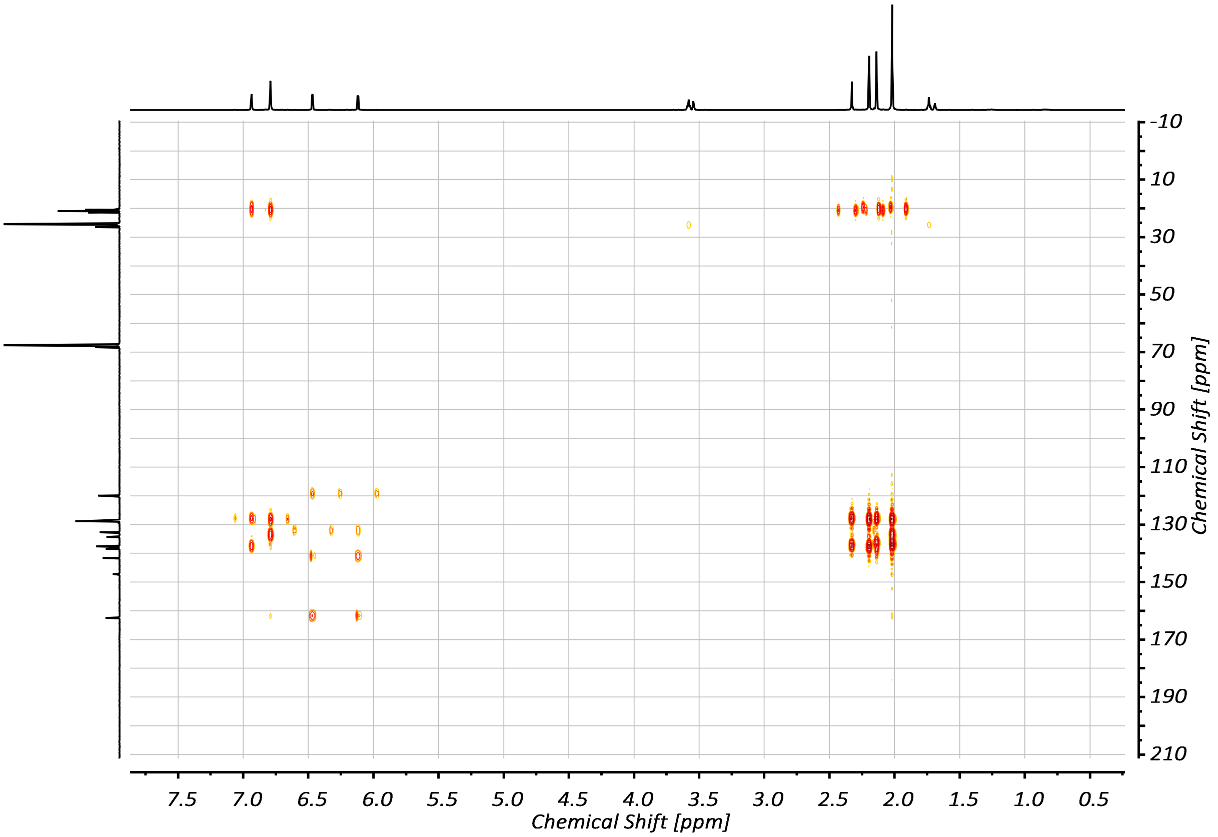

7.5
7.0
6.5
6.0
5.5
5.0
4.5
4.0
3.5
3.0
2.5
2.0
1.5
1.0
0.5
Chemical Shift [ppm]
-10
10
30
50
70
90
110
130
150
170
190
210
Chemical Shift [ppm]

Figure S10. High-temperature ^{13}C NMR spectra of (^{Mes}DPM)MgNC(THF)$_2$ (**4**) in THF-d_8 (*), measured at 25°C (black) and 45°C (red).

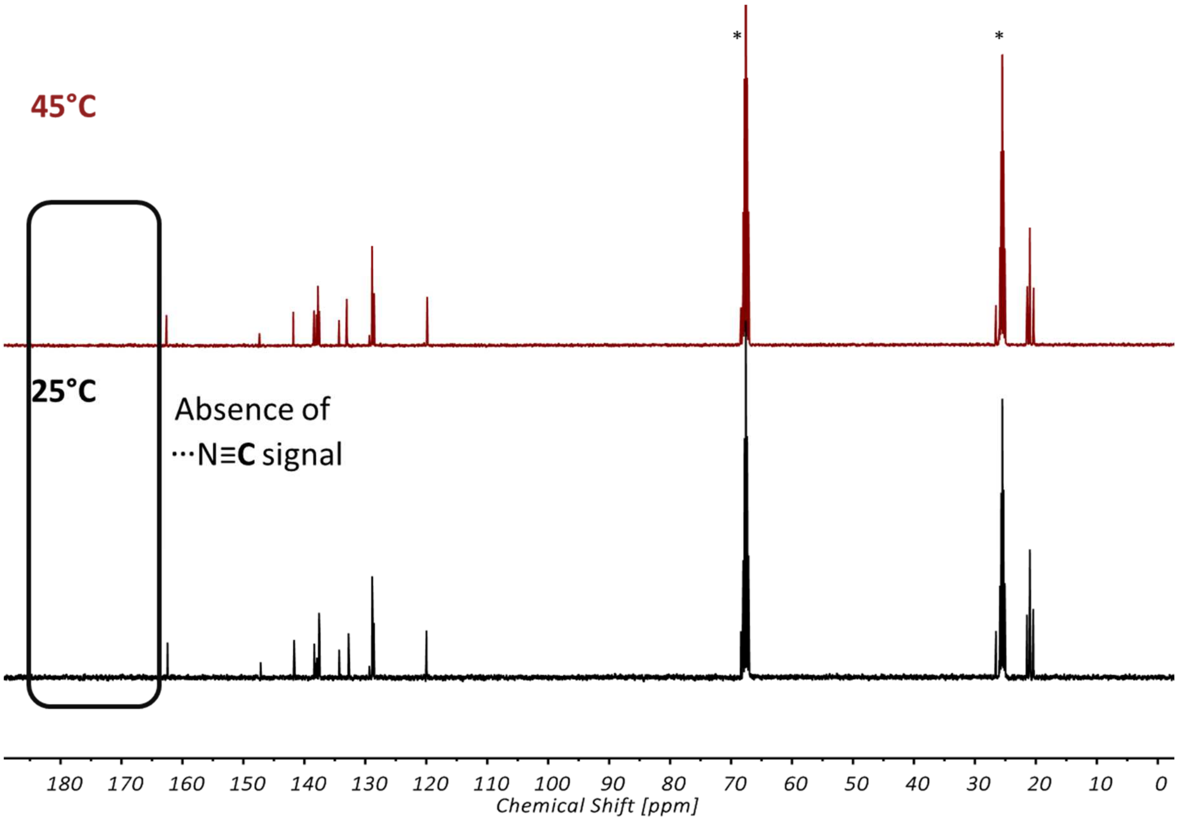

Figure S11. High-temperature ^{13}C NMR spectra of (^{Mes}DPM)MgN^{13}C(THF)$_2$ in THF-d_8 (*), measured from 25°C (black) to 55°C (green).

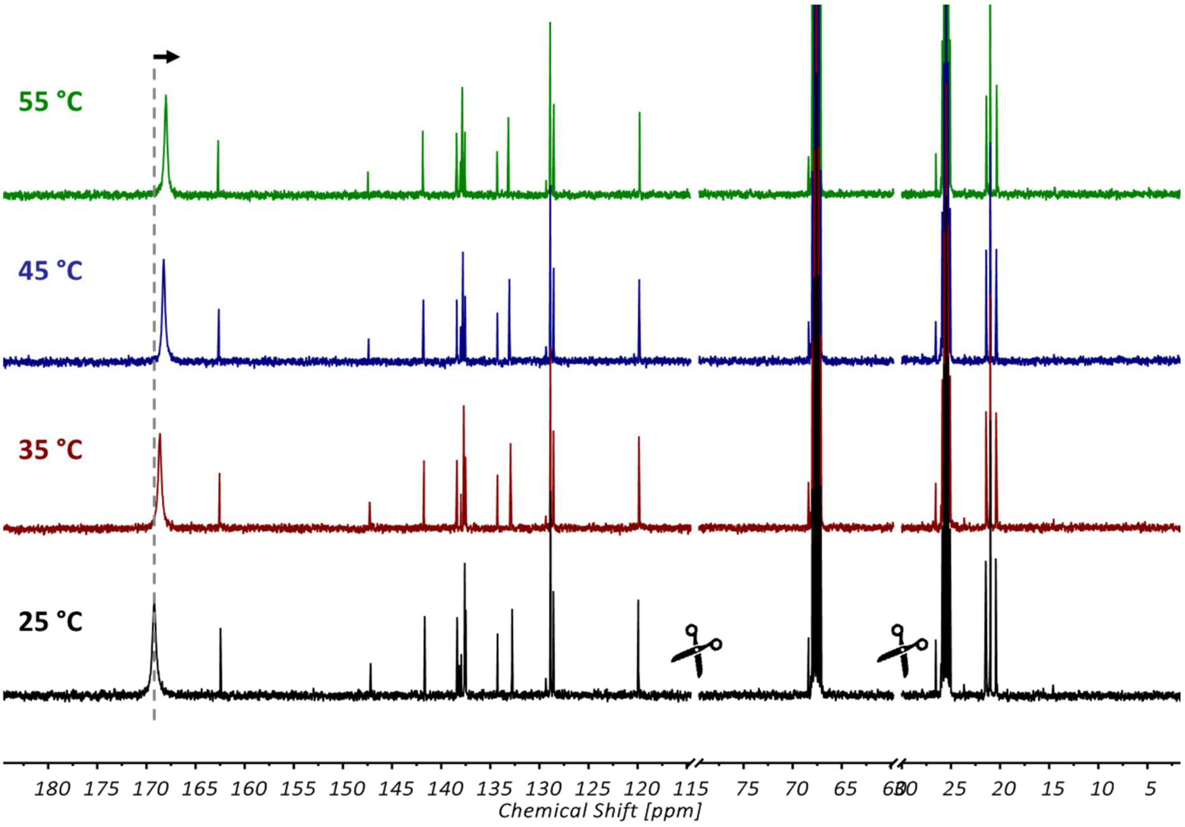

Figure S12. Low-temperature ^{13}C NMR spectra of (^{Mes}DPM)MgN^{13}C(THF)$_2$ in THF-*d₈* (*), measured from 20°C to -85°C.

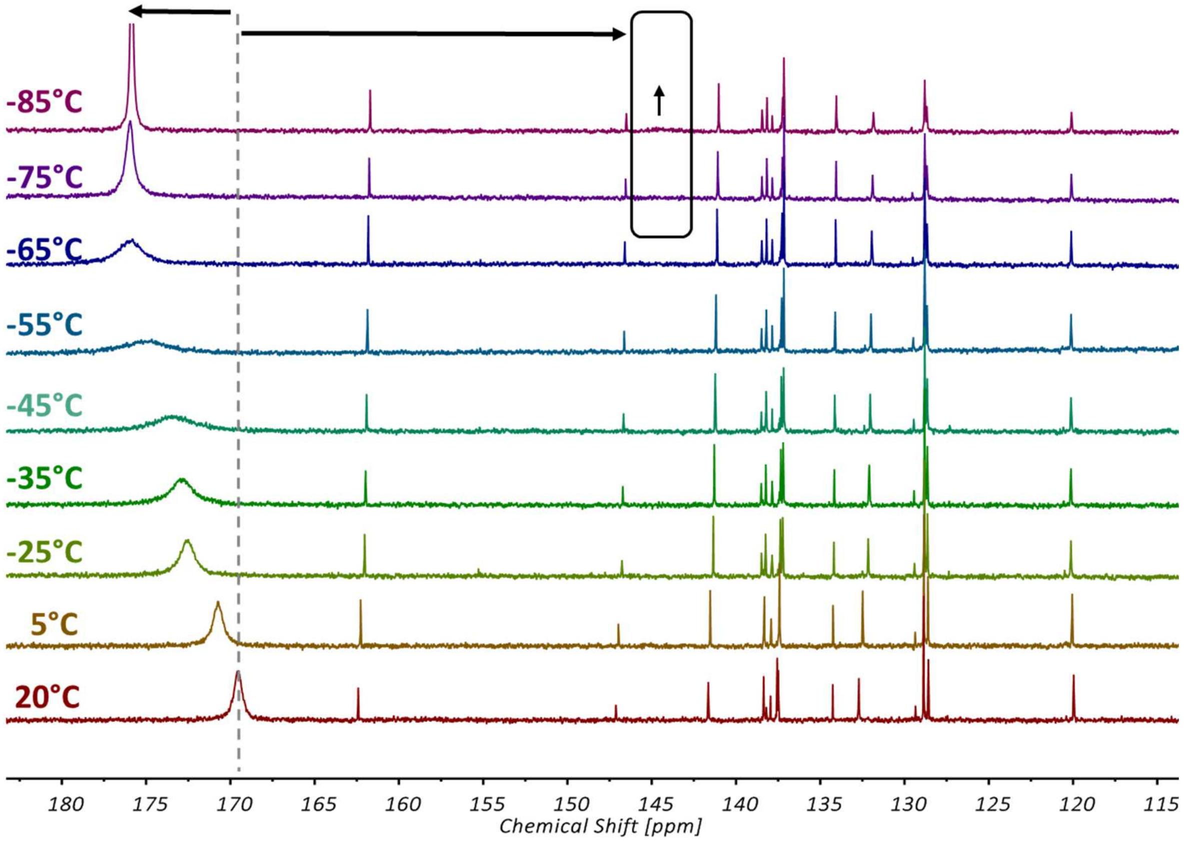

Figure S13. Low-temperature ^{13}C NMR spectra of (^{Mes}DPM)MgN^{13}C(THF)$_2$ in THF-d_8 (*), measured at -86°C.

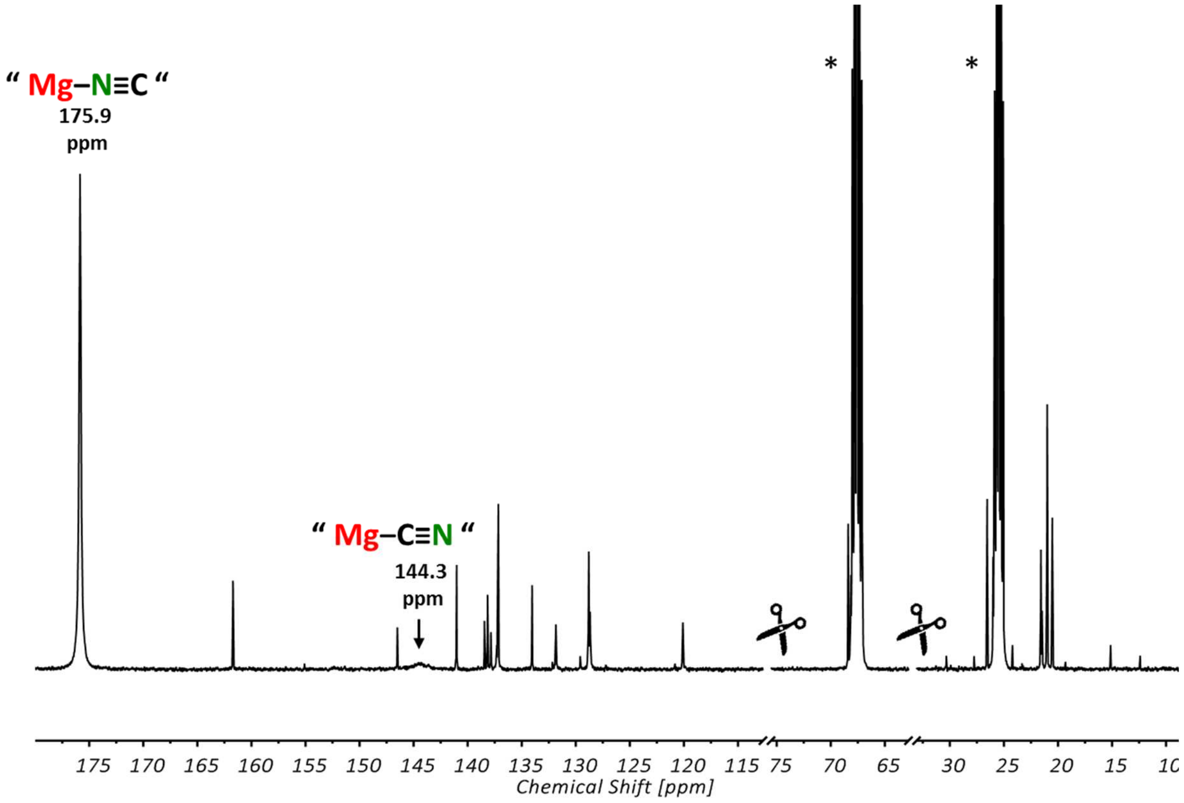

Diffusion-Ordered-Spectroscopy (DOSY)

Figure S14. DOSY NMR spectrum of (^{Mes}DPM)MgNC(THF)$_2$ (**4**) in THF-d_8.

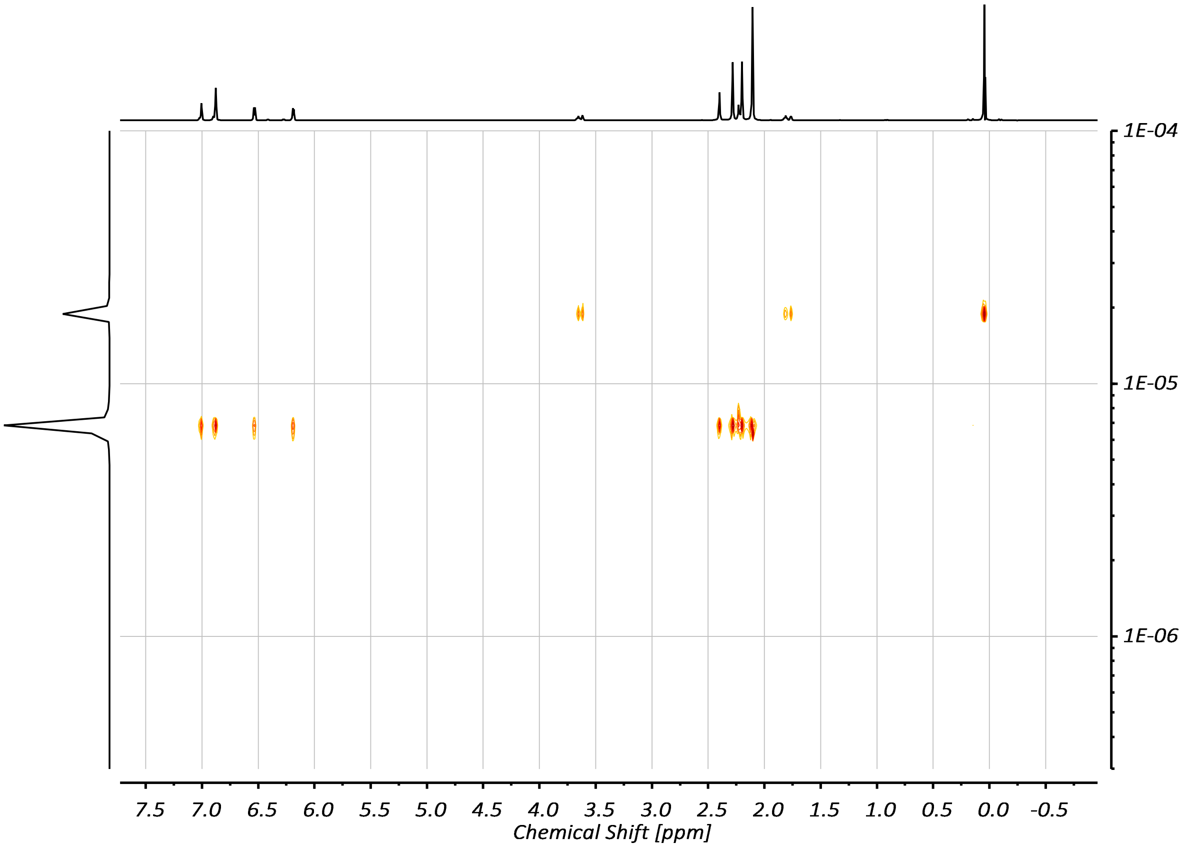

Table S5. DOSY parameter for (^{Mes}DPM)MgNC(THF)$_2$ (**4**) in THF-*d8*.

**Formula**	**D [m²/s]**	**MW** $^{Exp.}_{Monomer}$ **[g/mol]**	**MW** $^{Calc.}_{Monomer}$ **[g/mol]**
**$C_{37}H_{37}N_3Mg$**	$6.719*10^{-10}$	539 (**DMW**)<br>620 (**ECC**)	548 (without THF)<br>692 (with THF)

Diffusion measurements were conducted on a Bruker AVANCE NMR spectrometer operating at 600.13 MHz for proton resonance equipped with a 5 mm PABDO BB/19F-1H/D probe with Z-GRD and actively shielded gradient coil with a maximum gradient strength of 5.3500094 G/mm (at 10 A).

Parameter optimization was carried out empirically employing the pulse programme ledbpgp2s1D using stimulated echo and LED (D21 = 5 ms, longitudinal eddy current delay as a Z-filter) with bipolar gradient pulses (P30) and two spoiling gradients (P19 = 600 µs) leading to values for gradient pulse length (P30 = 1250 µs, in case of bipolar gradients *little DELTA*0.5*) and diffusion time (D20 = 60 ms, *big DELTA*). Delay for gradient recovery was set to 200 µs.

The diffusion experiment was executed with variable gradients from 2% to 98% gradient strength with 32 increment values (difframp calculated with the AU-program *DOSY*). In this case the pulse program ledbpgp2s was applied for data aquiring of this pseudo-2D Experiment. Data processing was performed

with the T1/T2 software package (SimFit) of TopSpin (version 3.2, Bruker Biospin) by fitting area data (integration of all peaks of interest of the same molecule) of diffusion decays. From these Stejskal-Tanner fitting curves calculated diffusion constants were obtained and assimilated statistically.

For molecular weight estimation Stalke´s method was applied (external calibration curves **ECC**´s under assumption of DSE-shaped molecules (dissipated spheres and ellipsoides) with tetramethylsilane (TMS) as internal reference with normalized diffusion coefficients.[9,10]

4) Selected infrared spectra

IR vibrational spectra, shown in **Figure S15** – **S17**, were recorded from 3600 to 400 cm^{-1} (Shimadzu, IRAffinity-1) as KBr pellets at room temperature.

Figure S15. IR vibrational spectrum of (^{Mes}DPM)MgN^{12}C(THF)$_2$ (**4**).

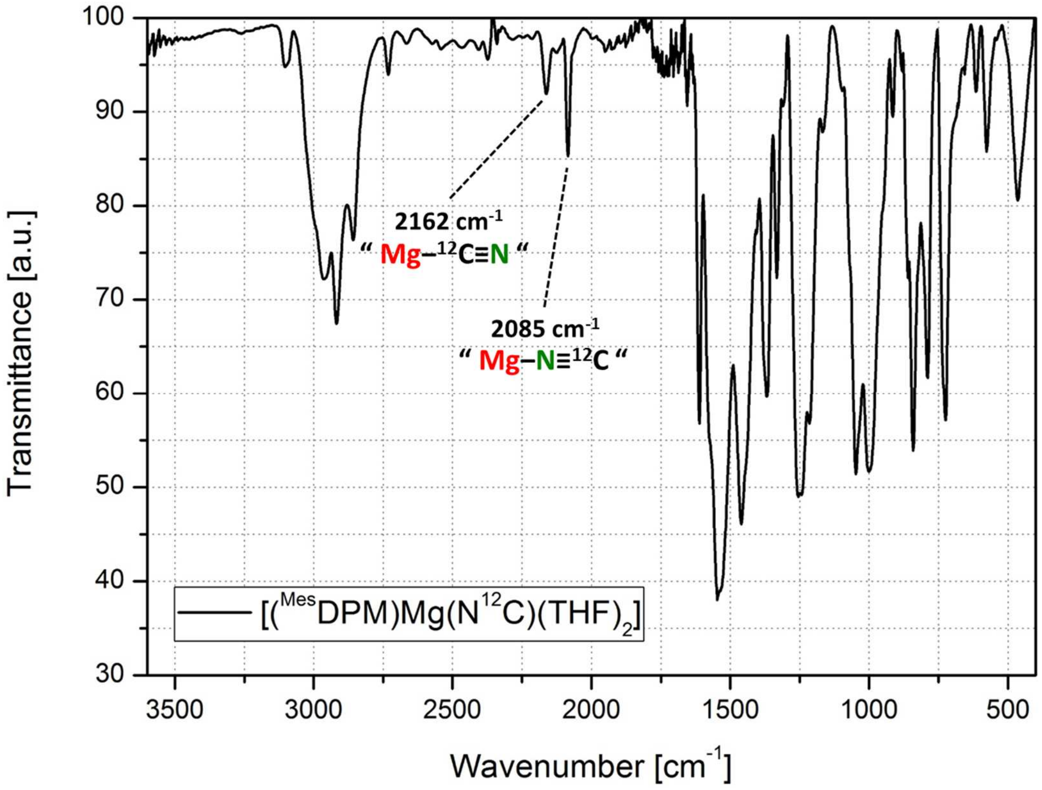

Figure S16. IR vibrational spectrum of (^{Mes}DPM)MgN^{13}C(THF)$_2$.

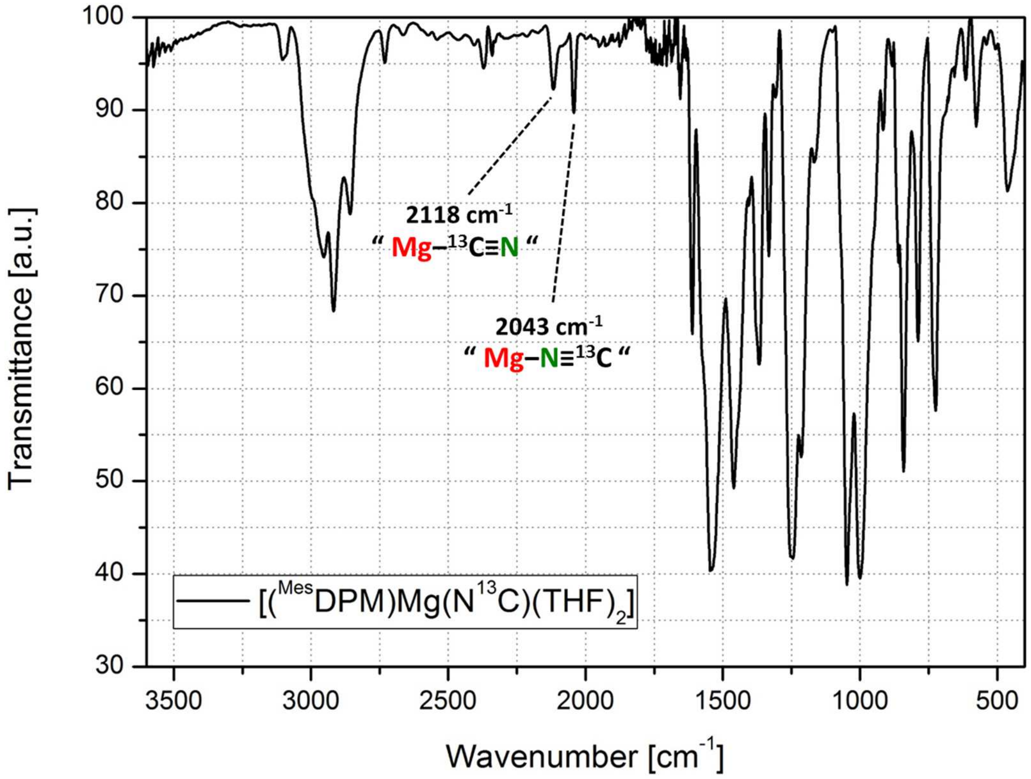

Figure S17. Superimposed IR vibrational spectra of (^{Mes}DPM)MgN^{12}C(THF)$_2$ (black) and (^{Mes}DPM)MgN^{13}C(THF)$_2$ (red).

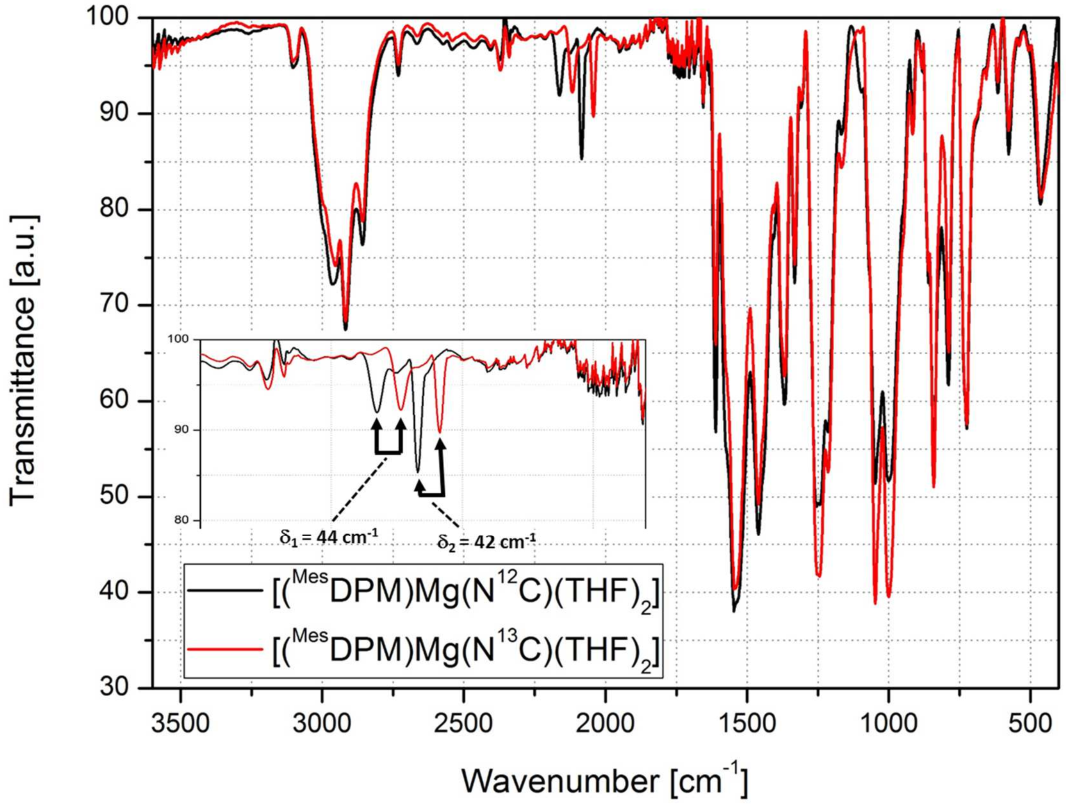

IR vibrational spectra, shown in **Figure S18** – **S21**, were recorded on a Bruker Alpha II Platinum ATR.

Figure S18. IR vibrational spectrum of (^{Mes}DPM)MgN^{12}C(THF)$_2$.

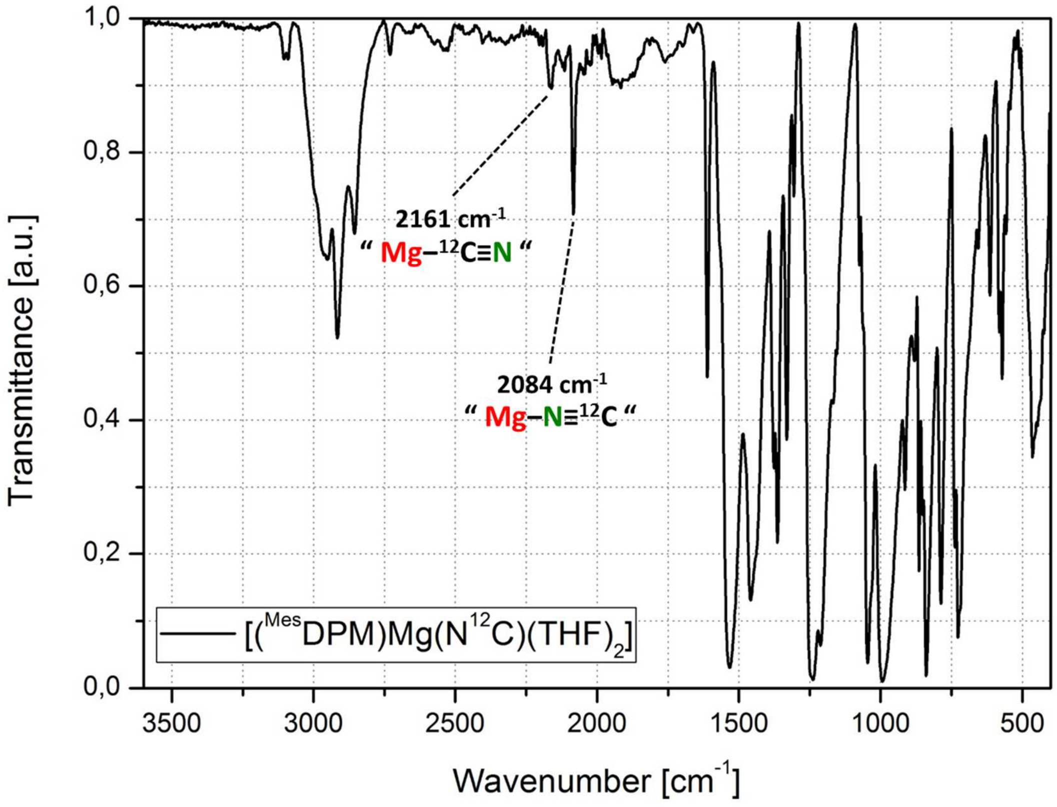

Figure S19. IR vibrational spectrum of (^{Mes}DPM)MgN^{13}C(THF)$_2$.

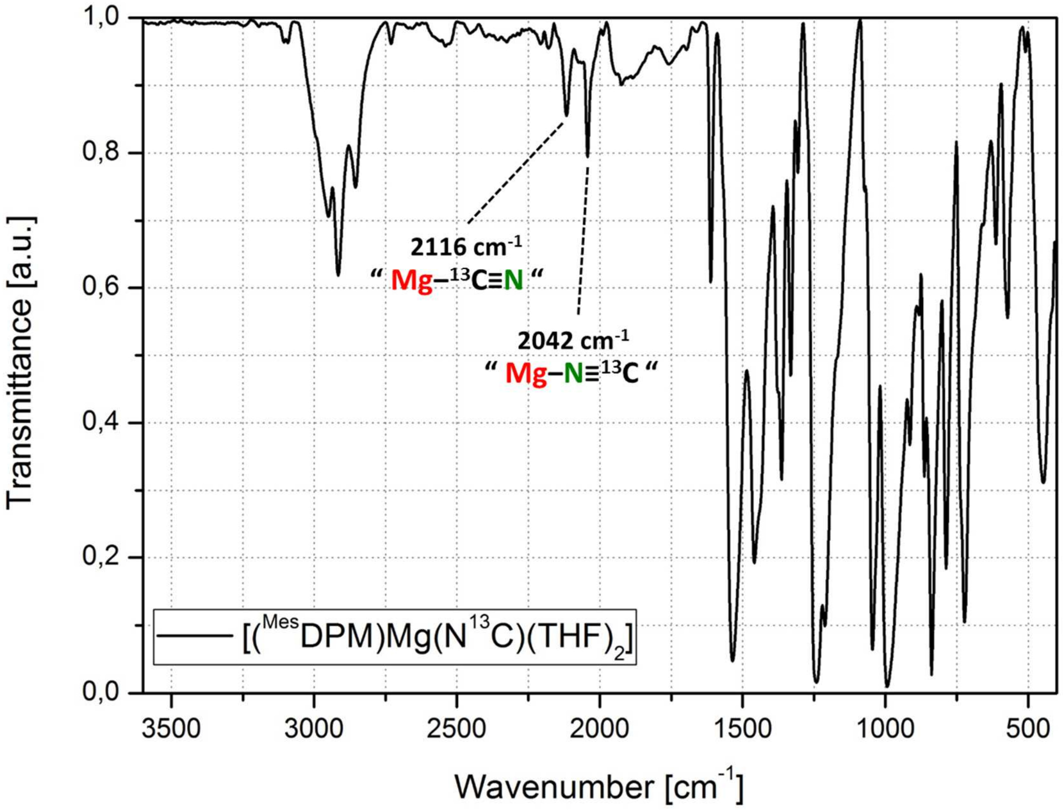

Figure S20. Superimposed (top left) and stacked (top right) IR vibrational spectra of (^{Mes}DPM)MgN^{12}C(THF)$_2$ from 30°C to 70°C and superimposed (bottom left) and stacked (bottom right) IR vibrational spectra of (^{Mes}DPM)MgN^{12}C(THF)$_2$ upon cooling from 70°C to 30°C.

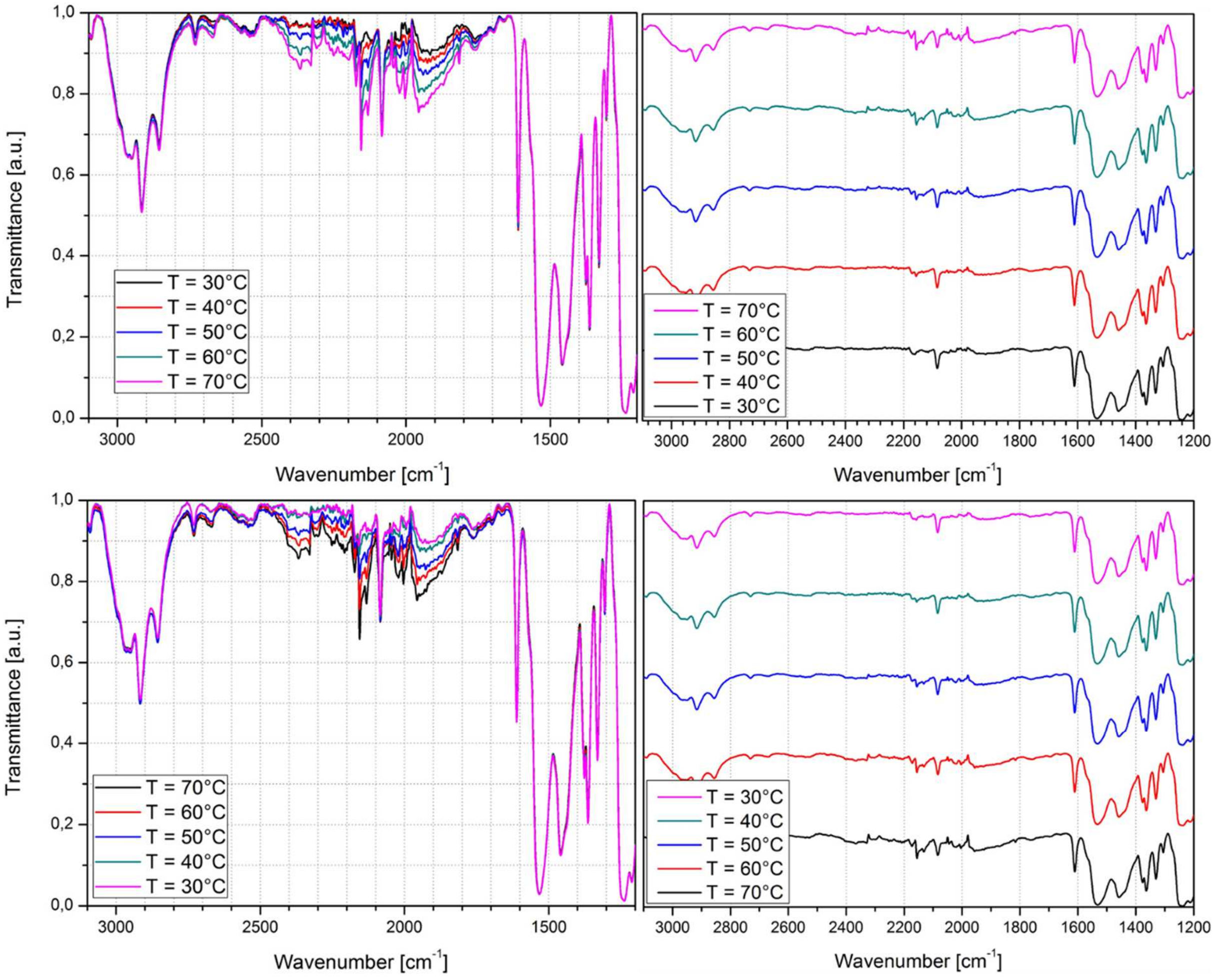

Figure S21. Superimposed (top left) and stacked (top right) IR vibrational spectra of ^{13}C-labeled (^{Mes}DPM)MgN^{13}C(THF)$_2$ from 30°C to 70°C and superimposed (bottom left) and stacked (bottom right) IR vibrational spectra of ^{13}C-labeled (^{Mes}DPM)MgN^{13}C(THF)$_2$ upon cooling from 70°C to 30°C.

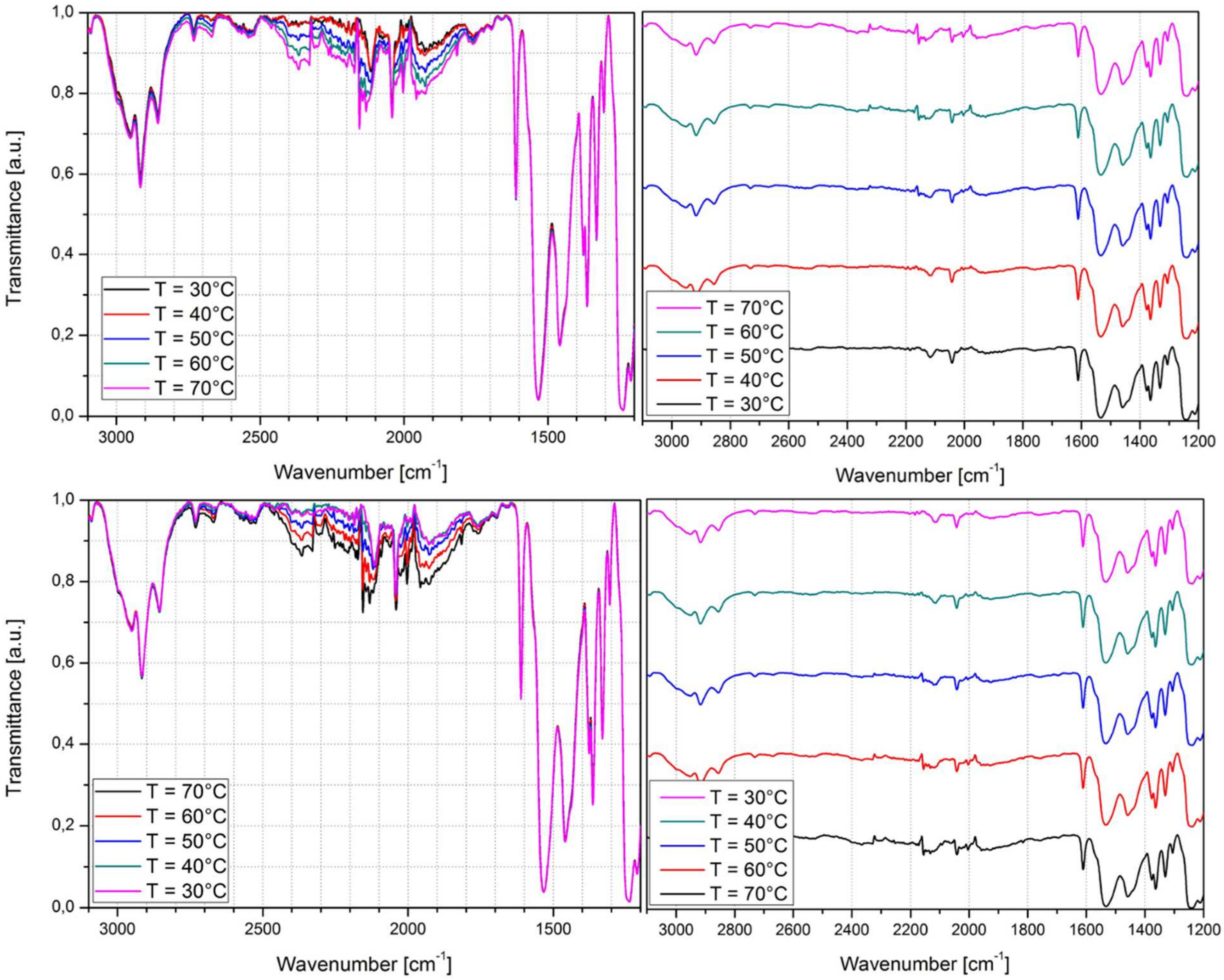

Theoretical prediction of IR signals of (^{Mes}DPM)MgN^{12}C(THF)$_2$ and (^{Mes}DPM)MgN^{13}C(THF)$_2$

In accordance with Hooke´s law,[11]

$$v_{max} = \frac{1}{2\pi c}\sqrt{\frac{k}{\mu}}$$

where c = speed of light, *k* = force constant, $\mu = \frac{m1m2}{m1+m2}$ reduced mass.

given the mass of ^{12}C = 12.011 u, ^{13}C = 13.003 u and ^{14}N = 14.007 u

In the case of ^{12}C-^{14}N stretch for (^{Mes}DPM)MgN^{12}C(THF)$_2$:

$$v_{max} = \frac{1}{2\pi c}\sqrt{\frac{k}{\mu}} = \frac{1}{2\pi c}\sqrt{k}\sqrt{\frac{m1+m2}{m1m2}}$$

$$2085 = \frac{1}{2\pi c}\sqrt{k}\sqrt{\frac{m1+m2}{m1m2}} = \frac{1}{2\pi c}\sqrt{k}\sqrt{\frac{12.011+14.007}{12.011\text{x}14.007}} = \frac{1}{2\pi c}\sqrt{k}\ 0.393$$

$$\frac{1}{2\pi c}\sqrt{k} = 5302$$

In the case of ^{13}C-^{14}N stretch of (^{Mes}DPM)MgN^{13}C(THF)$_2$ for (^{Mes}DPM)MgN^{12}C(THF)$_2$

assuming that the force constant is similar in both (^{Mes}DPM)MgN^{12}C(THF)$_2$ and (^{Mes}DPM)MgN^{13}C(THF)$_2$

$$v_{max} = \frac{1}{2\pi c}\sqrt{\frac{k}{\mu}} = \frac{1}{2\pi c}\sqrt{k}\sqrt{\frac{m1+m2}{m1m2}}$$

$$v_{max} = \frac{1}{2\pi c}\sqrt{k}\sqrt{\frac{13.003+14.007}{13.003\text{x}14.007}} = 5302\sqrt{\frac{13.003+14.007}{13.003\text{x}14.007}} = 2042$$

Theoretical v_{max} for ^{13}C-^{14}N stretch of (^{Mes}DPM)MgN^{13}C(THF)$_2$ calculated from (^{Mes}DPM)MgN^{12}C(THF)$_2$ is 2042 cm^{-1}.

Experimental v_{max} was found to be 2042 cm^{-1} (**ATR**), 2043 cm^{-1} (**KBr**).

Analogous for (^{Mes}DPM)Mg^{12}CN(THF)$_2$ and (^{Mes}DPM)Mg^{13}CN(THF)$_2$:

$$v_{max} = \frac{1}{2\pi c}\sqrt{\frac{k}{\mu}} = \frac{1}{2\pi c}\sqrt{k}\sqrt{\frac{m1+m2}{m1m2}}$$

$$2161 = \frac{1}{2\pi c}\sqrt{k}\sqrt{\frac{m1+m2}{m1m2}} = \frac{1}{2\pi c}\sqrt{k}\sqrt{\frac{12.011+14.007}{12.011\text{x}14.007}} = \frac{1}{2\pi c}\sqrt{k}\ 0.393$$

$$\frac{1}{2\pi c}\sqrt{k} = 5541$$

$$v_{max} = \frac{1}{2\pi c}\sqrt{k}\sqrt{\frac{13.003+14.007}{13.003\text{x}14.007}} = 5541\sqrt{\frac{13.003+14.007}{13.003\text{x}14.007}} = 2134$$

Theoretical v_{max} for ^{13}C-^{14}N stretch of (^{Mes}DPM)Mg^{13}CN(THF)$_2$ calculated from (^{Mes}DPM)Mg^{12}CN(THF)$_2$ is 2134 cm^{-1}.

Experimental v_{max} was found to be 2116 cm^{-1} (**ATR**), 2118 cm^{-1} (**KBr**).

Table S6. Overview of experimental and calculated IR vibrational CN stretch signals.

**Compound**	**Experimentally found wavenumbers [cm^{-1}]**	**Calculated wavenumbers using Hooke's law [cm^{-1}]**	**Calculated wavenumbers by DFT [cm^{-1}]^a**
**"MgN^{12}C"**	2085 (**KBr**)<br>2084 (**ATR**)	---	2099
**"Mg^{12}CN"**	2162 (**KBr**)<br>2161 (**ATR**)	---	2166
**"MgN^{13}C"**	2043 (**KBr**)<br>2042 (**ATR**)	2042	2065
**"Mg^{13}CN"**	2118 (**KBr**)<br>2116 (**ATR**)	2134	2127

^{a} B3PW91/6-311+G(2df,p); correction factor 0.9686.[12]

5) Theoretical calculations

General

All calculations were carried out using Gaussian 16A.[13] All methods were used as implemented. All structures were fully optimized on a B3PW91/6-311+G** level.[14-17] Specific structures were also optimized using Grimme's third dispersion method with Becke-Johnson dampening (GD3BJ).[18] Frequency calculations were carried out on the respective levels of theory in order to characterize them as true minima (NIMAG=0) or transition states (NIMAG=1). Charges were calculated via NBO analysis.[19] For IR spectra, structures were fully optimized on a B3PW91/6-311+G(2df,p) level of theory. A frequency scaling factor of 0.9686, as determined by Radom *et al*. was used.[12] Molecules were drawn and evaluated using Molecule V2.3.[20]

QTAIM analysis was carried out using AIMAll V17. Wave functions were obtained from the optimizations.[21,22]

The following model system was used: $(^{Ph}DPM)Mg(NC)\cdot(THF)_2$

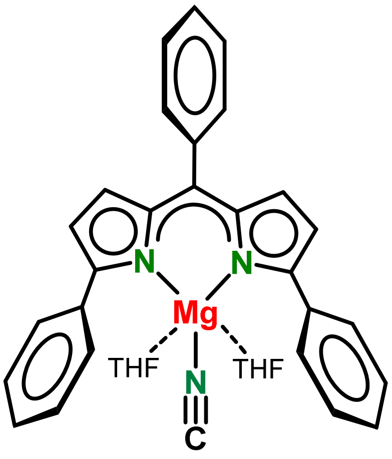

$(^{Ph}DPM)Mg(NC).(THF)_2$

Figure S22. Chemical formula of used model system $(^{Ph}DPM)Mg(NC)\cdot(THF)_2$.

Energies

Table S7. Calculated enthalpies (ΔH).

ΔH [kcal/mol]	B3PW91/6-311+G**	B3PW91(GD3BJ)/6-311+G**
$(^{Ph}DPM)Mg(THF)_2$–N≡C	0.00	0.00
TS Side on	+10.7	+10.91
$(^{Ph}DPM)Mg(THF)_2$–C≡N	+0.90	+1.60

Table S8. Calculated Gibbs free energies (ΔG) and entropies (ΔS, in brackets).

ΔG [kcal/mol] (ΔS [cal/mol*K]) (298K)	B3PW91/6-311+G**	B3PW91(GD3BJ)/6-311+G**
(^{Ph}DPM)Mg(THF)$_2$–N≡C	0.00 (0.00)	0.00 (0.00)
TS Side on	12.21 (-4.93)	12.00 (-3.66)
(^{Ph}DPM)Mg(THF)$_2$–C≡N	0.83 (0.23)	1.63 (-0.08)

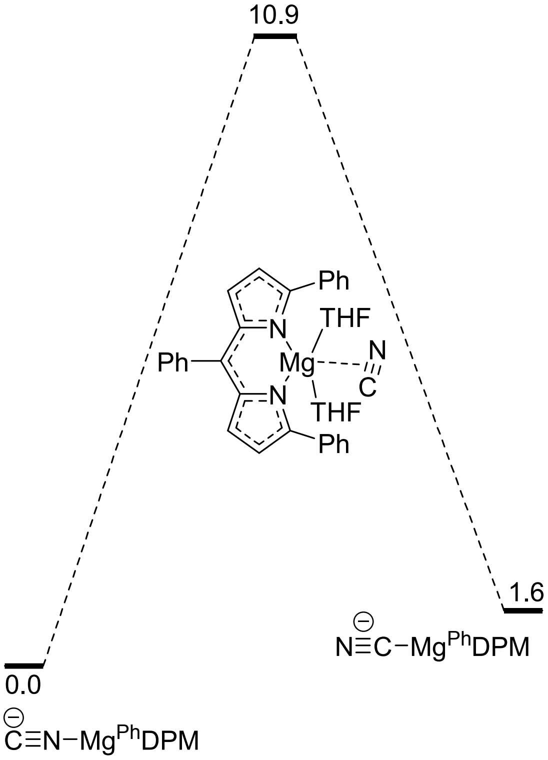

Figure S23. Energy diagram for NC/CN isomerization in (^{Ph}DPM)Mg(NC)·(THF)$_2$; B3PW91(GD3BJ)/6-311+G**, ΔH in kcal/mol.

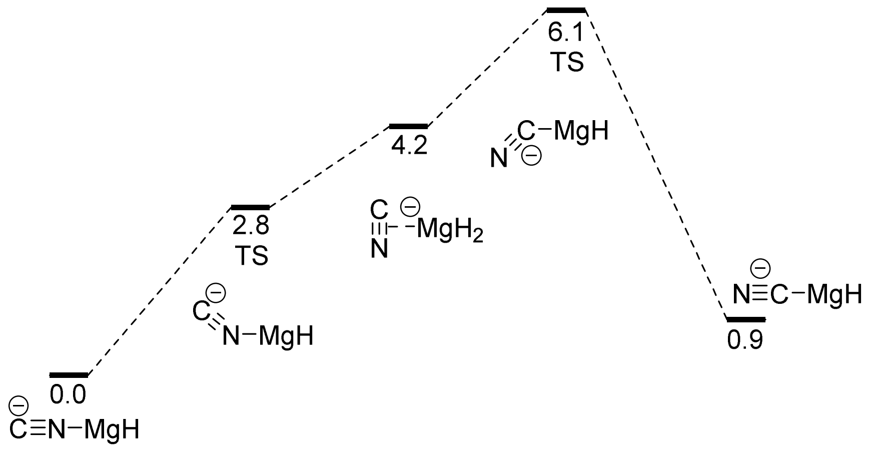

Figure S24. Energy diagram for NC/CN isomerization in H-Mg(NC) ; B3PW91/6-311+G**, ΔH in kcal/mol.

Table S9. Calculated ΔH, ΔG and ΔS for isomerization of H-Mg–N≡C to H-Mg–C≡N (B3PW91/6-311+G**).

Structure	ΔH (gas phase) kcal/mol	ΔG (gas phase) kcal/mol	ΔS (gas phase) cal/mol*K
H-Mg–N≡C	0.00	0.00	0.00
TS H-Mg–N≡C	2.82	0.07	9.22
H-Mg NC Side on	4.19	0.92	10.97
TS H-Mg–C≡N	6.13	3.34	9.34
H-Mg–C≡N	0.93	-1.03	6.59

Charges and bond parameters

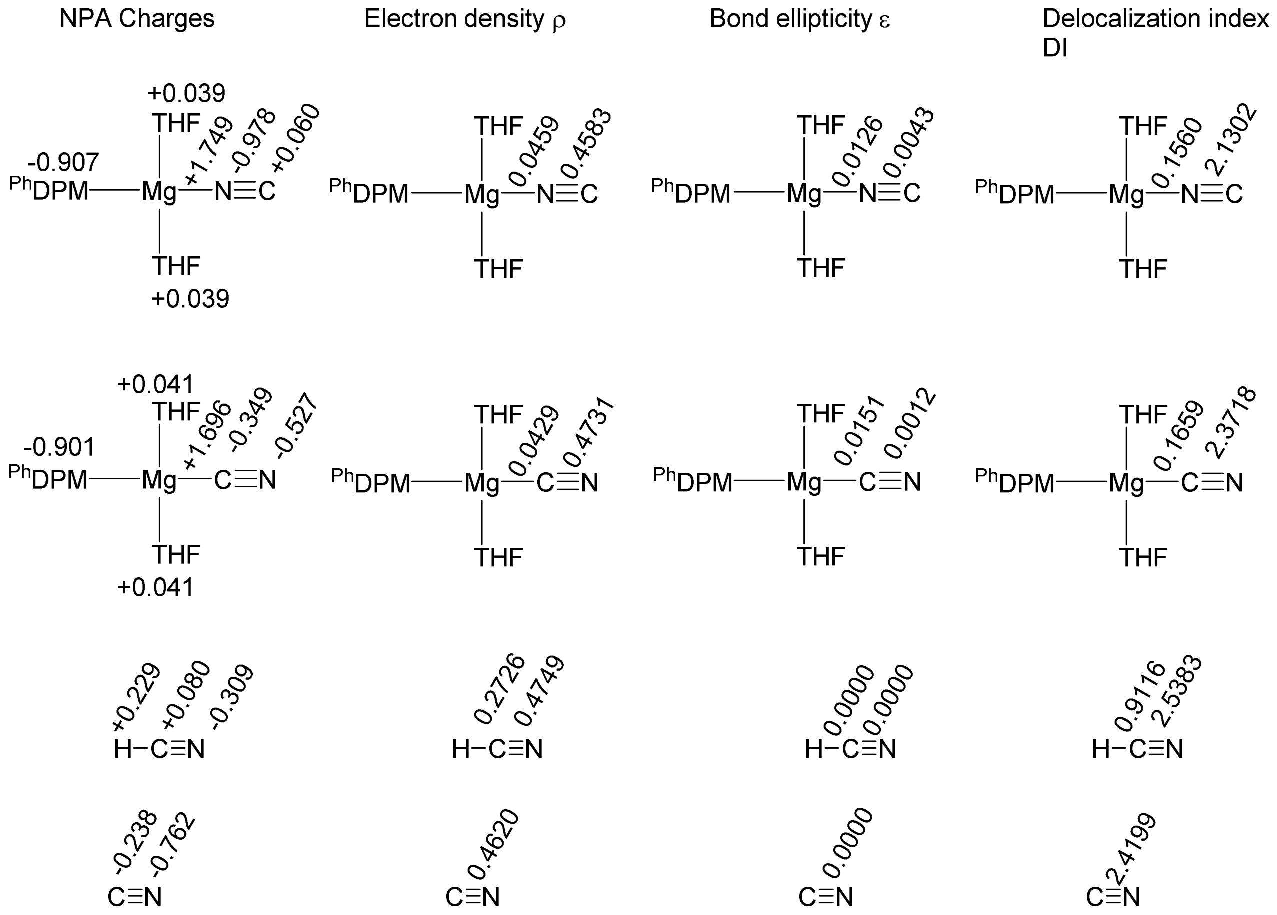

Figure S25. Selected charges and bond parameters. All charges and bond parameters were determined at the B3PW91/6-311+G** level of theory.

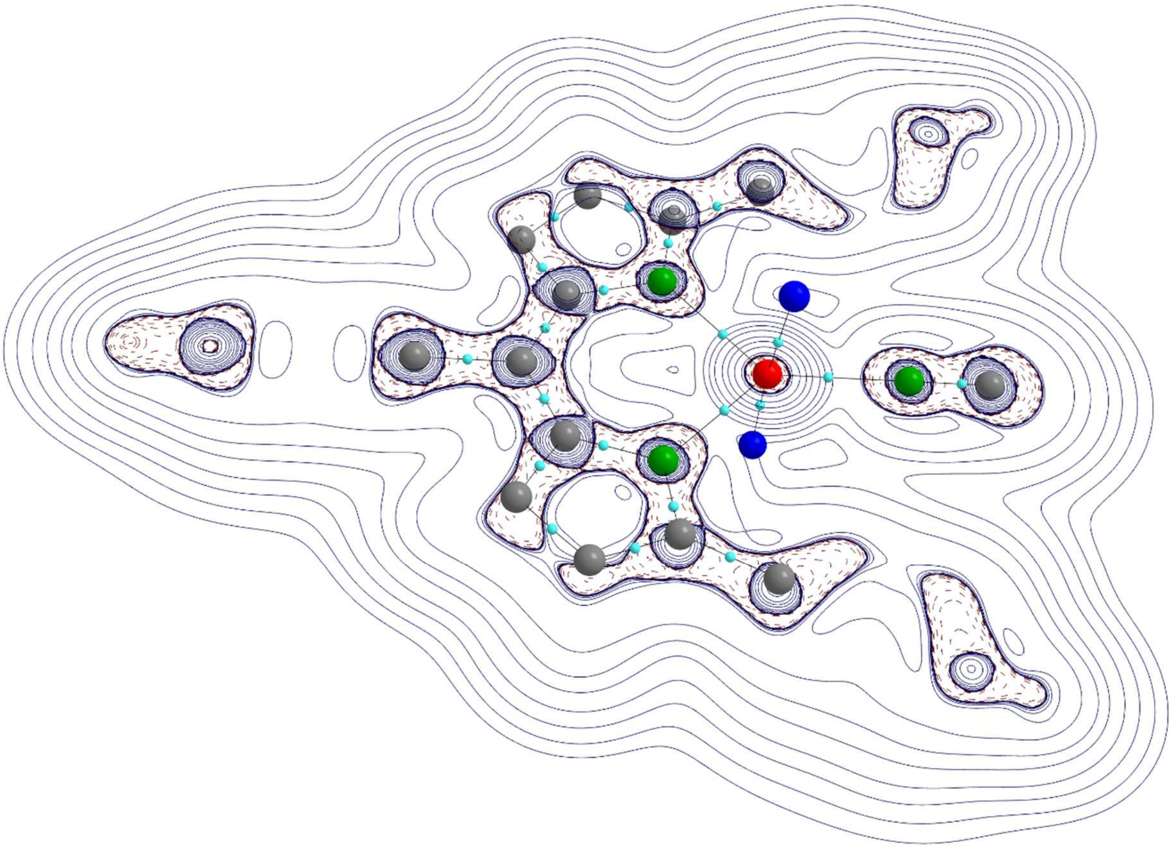

Figure S26. Laplacian of the electron density for (^{Ph}DPM)Mg(N≡C)·(THF)$_2$.

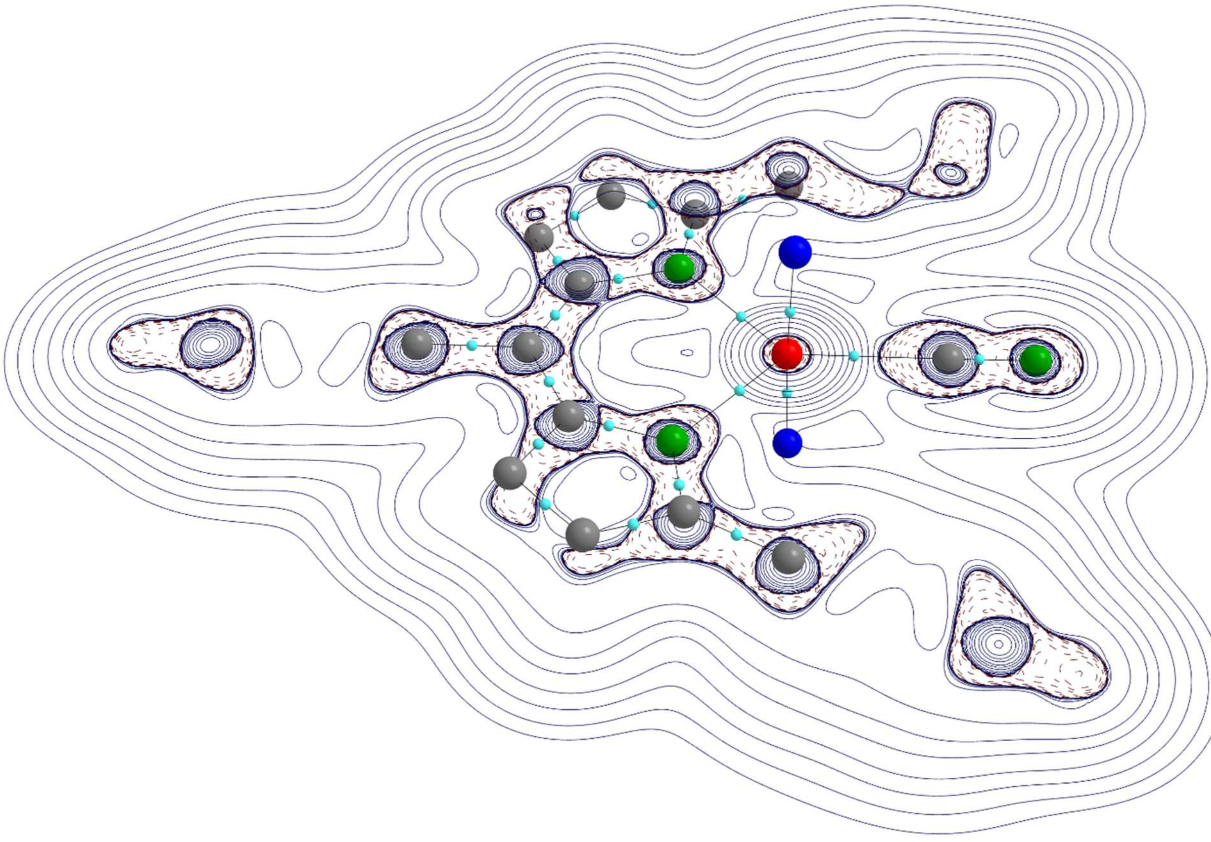

Figure S27. Laplacian of the electron density for (^{Ph}DPM)Mg(C≡N)·(THF)$_2$.

Calculated IR spectra

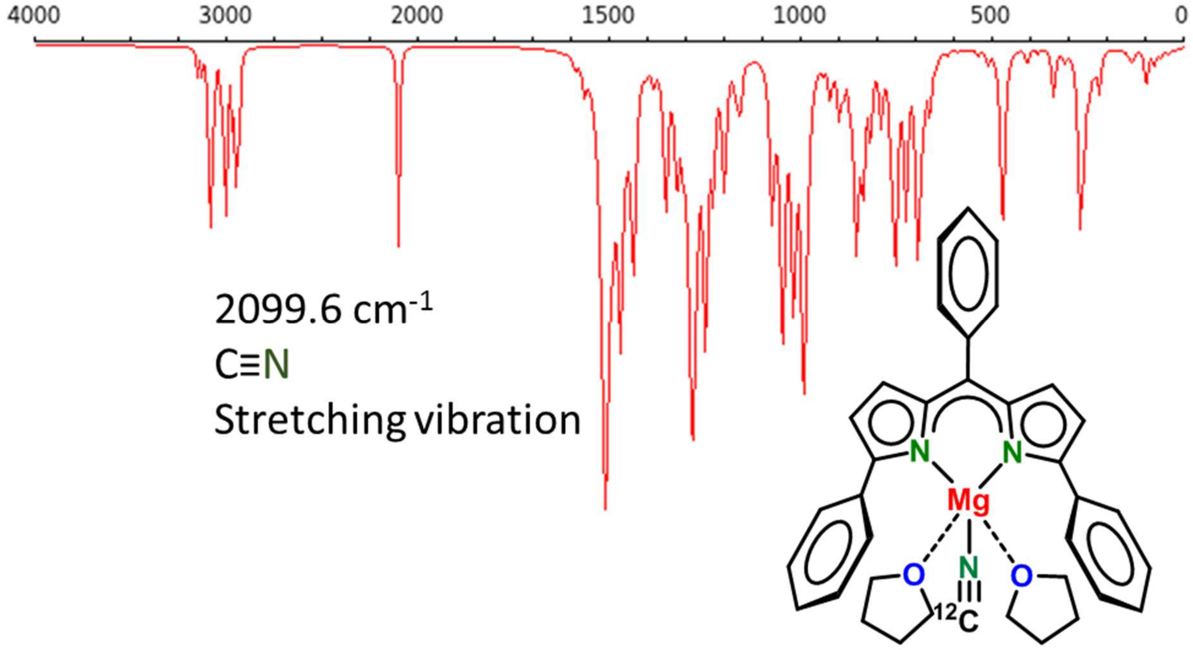

Figure S28. Calculated IR spectrum for (^{Ph}DPM)Mg(N≡^{12}C)·(THF)$_2$, B3PW91/6-311+G(2df,p).

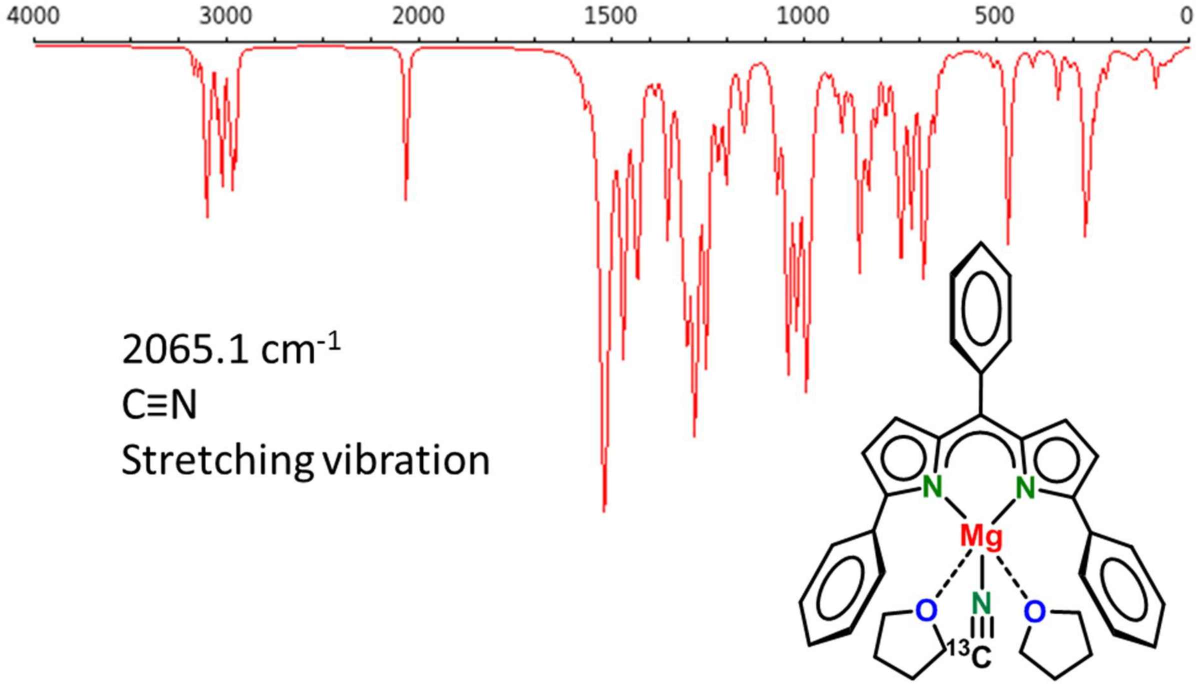

Figure S29. Calculated IR spectrum for (^{Ph}DPM)Mg(N≡^{13}C)·(THF)$_2$, B3PW91/6-311+G(2df,p).

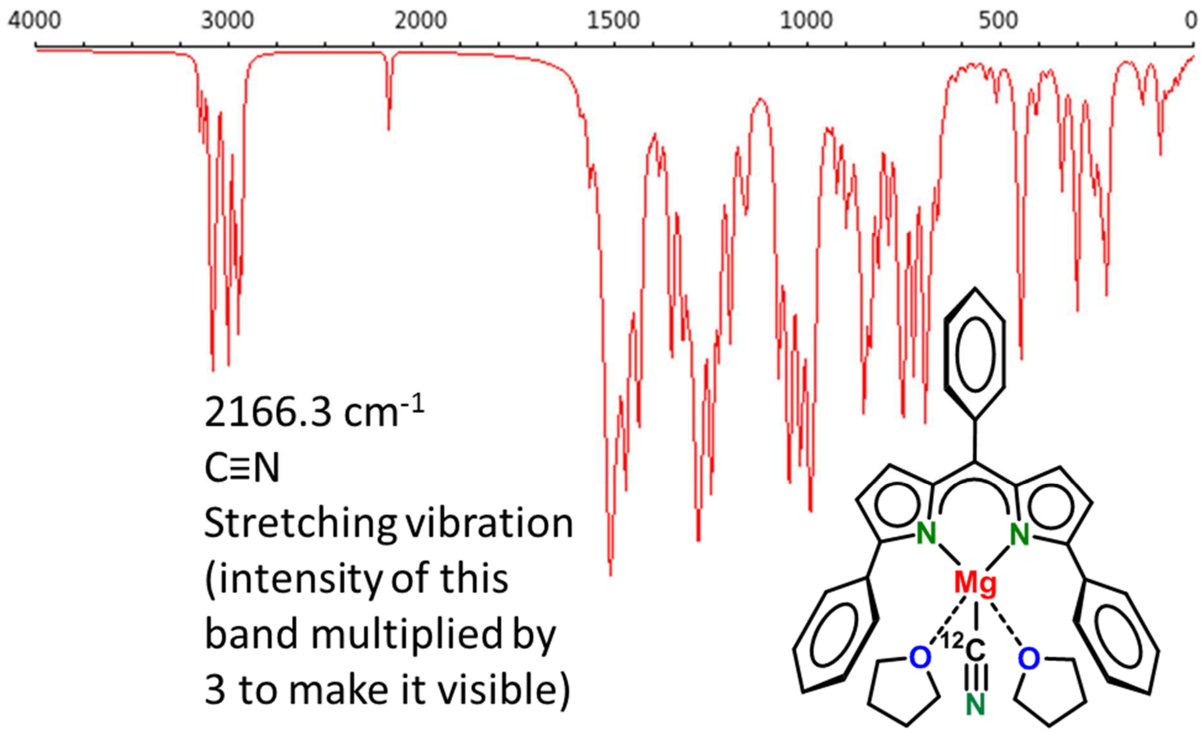

Figure S30. Calculated IR spectrum for (^{Ph}DPM)Mg(^{12}C≡N)·(THF)$_2$, B3PW91/6-311+G(2df,p).

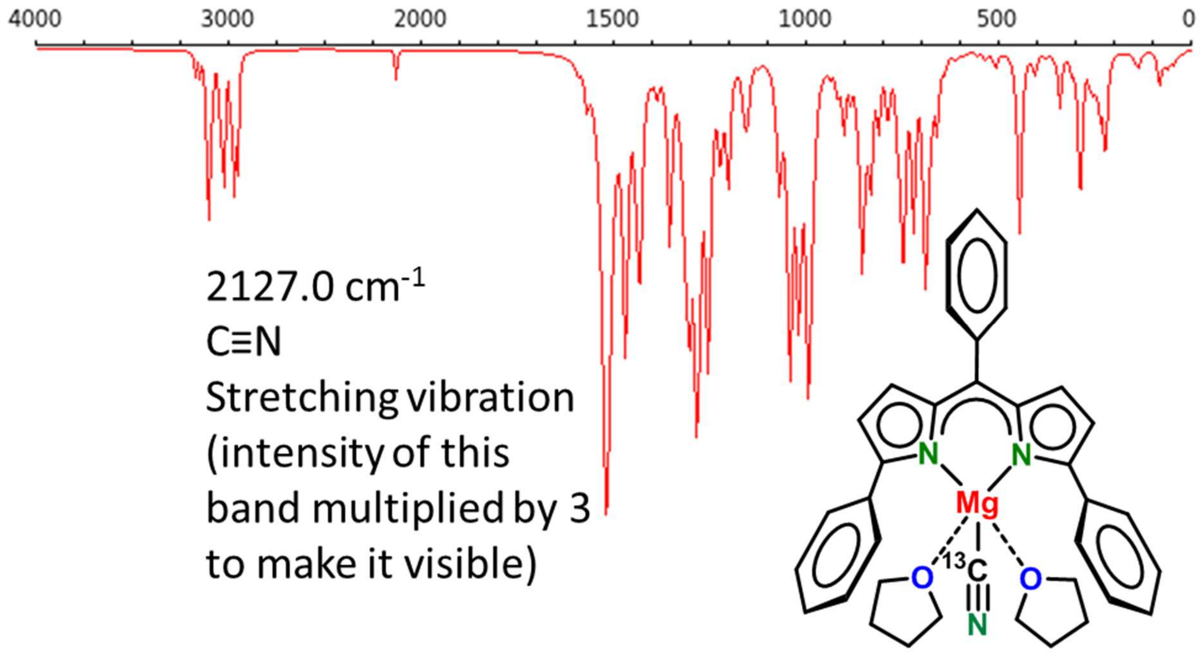

Figure S31. Calculated IR spectrum for (^{Ph}DPM)Mg(^{13}C≡N)·(THF)$_2$, B3PW91/6-311+G(2df,p).

XYZ coordinates

	77		
LMg(THF)2CN		B3PW91(GD3BJ)/6-311+G**	
Mg	1.028502	-0.058653	0.070209
O	0.937818	-0.856235	2.045400
O	1.230308	0.763785	-1.877628
N	-0.390835	1.444418	0.505258
N	-0.519862	-1.378283	-0.504265
N	4.336219	-0.320212	0.214234
C	-1.754359	1.332305	0.264932
C	-0.130098	2.751607	0.678119
C	0.888581	-3.345536	-0.924187
C	-0.403236	-2.702589	-0.704688
C	-2.435166	0.142353	-0.043973
C	-1.873308	-1.114783	-0.324539
C	1.945241	-2.655343	-1.528937
C	1.221763	3.242095	0.926647
C	-3.917452	0.224500	-0.079049
C	-2.338167	2.637201	0.300627
H	-3.381616	2.862918	0.141361
C	3.196451	-3.243712	-1.651464
H	4.012686	-2.674470	-2.079931
C	-1.316659	3.527376	0.546411
H	-1.391241	4.599364	0.656303
C	2.166823	2.449252	1.588032
C	-4.618561	-0.092330	-1.245630
C	3.404934	-4.541887	-1.199584
C	-4.631287	0.621712	1.054767
C	0.074919	0.985018	-2.718905
H	-0.748051	1.321197	-2.083287
H	-0.195393	0.031669	-3.175733
C	1.613904	4.502115	0.452643
C	-1.675589	-3.339321	-0.643927
H	-1.866856	-4.392517	-0.788607
C	1.105276	-4.660124	-0.486725
C	2.352169	-5.251905	-0.624933
H	2.509919	-6.264751	-0.269296
C	-2.598664	-2.343663	-0.416951
H	-3.667372	-2.451354	-0.310858
C	-0.288636	-0.834137	2.813333
H	-1.124130	-0.853423	2.111232
H	-0.319355	0.099869	3.377457
C	2.922436	4.936505	0.606005
H	3.215797	5.907640	0.221175
C	1.664149	-2.081035	2.312438
H	2.449737	-1.863983	3.043147
H	2.134523	-2.404526	1.384659
C	3.863054	4.120751	1.232669

C	3.480161	2.877895	1.724790
H	4.205722	2.225317	2.195439
C	-6.005197	-0.007130	-1.279209
H	-6.537365	-0.243616	-2.194443
C	0.616253	-3.029284	2.857719
H	1.053154	-3.841253	3.441657
H	0.039921	-3.460584	2.034460
C	1.512940	2.858491	-2.898188
H	2.184731	3.461269	-3.511774
H	1.004957	3.517830	-2.189013
C	-6.018858	0.692238	1.024857
H	-6.562451	0.989777	1.915226
C	-6.708893	0.381455	-0.143095
C	-0.256502	-2.084015	3.682881
H	-1.258738	-2.472818	3.871523
H	0.216449	-1.874516	4.647099
C	2.245749	1.762689	-2.149893
H	3.029120	1.296064	-2.754635
H	2.680108	2.074386	-1.200842
C	3.177148	-0.238746	0.162134
C	0.493842	2.059555	-3.709985
H	-0.355657	2.655474	-4.048460
H	0.971015	1.612831	-4.587654
H	-4.088091	0.864124	1.961388
H	-7.791655	0.442262	-0.167978
H	-4.064907	-0.394753	-2.127538
H	4.890942	4.452006	1.332370
H	0.892517	5.125355	-0.064974
H	1.874678	1.490910	2.003522
H	4.385038	-4.997736	-1.287947
H	1.788031	-1.651653	-1.907955
H	0.297170	-5.203770	-0.009202

	77		
LMg(THF)2NC		B3PW91(GD3BJ)/6-311+G**	
Mg	-1.024798	-0.000001	-0.000001
O	-1.097327	-0.841750	-1.940626
O	-1.097333	0.841747	1.940622
N	0.461039	1.408219	-0.517727
N	0.461042	-1.408217	0.517727
N	-3.063331	-0.000006	-0.000003
C	1.822498	1.220896	-0.310983
C	0.272431	2.725576	-0.708809
C	-1.051615	-3.294905	0.937890
C	0.272439	-2.725575	0.708809
C	2.444637	0.000004	0.000001
C	1.822501	-1.220889	0.310985
C	-2.061260	-2.550710	1.558251
C	-1.051624	3.294903	-0.937891

C	3.929646	0.000006	0.000002
C	2.479384	2.488856	-0.385463
H	3.538020	2.655952	-0.257444
C	-3.343642	-3.066509	1.686065
H	-4.121214	-2.454867	2.128010
C	1.505304	3.432543	-0.622127
H	1.639232	4.496058	-0.755296
C	-2.061267	2.550704	-1.558253
C	4.638439	-0.340111	1.155311
C	-3.631365	-4.345683	1.223725
C	4.638439	0.340125	-1.155307
C	0.099177	0.982343	2.740490
H	0.927892	1.226844	2.071516
H	0.299035	0.021876	3.218220
C	-1.348166	4.590778	-0.490759
C	1.505314	-3.432537	0.622129
H	1.639245	-4.496052	0.755299
C	-1.348151	-4.590783	0.490760
C	-2.626123	-5.110320	0.633772
H	-2.845260	-6.108945	0.270500
C	2.479392	-2.488847	0.385465
H	3.538028	-2.655940	0.257447
C	0.099186	-0.982340	-2.740492
H	0.927901	-1.226834	-2.071517
H	0.299037	-0.021873	-3.218224
C	-2.626139	5.110311	-0.633771
H	-2.845280	6.108934	-0.270498
C	-2.013765	-1.931844	-2.217061
H	-2.811656	-1.549953	-2.860869
H	-2.453559	-2.252965	-1.274287
C	-3.631378	4.345671	-1.223725
C	-3.343650	3.066498	-1.686067
H	-4.121220	2.454854	-2.128013
C	6.028106	-0.332746	1.157043
H	6.566988	-0.586447	2.063704
C	-1.163746	-2.979377	-2.907860
H	-1.759396	-3.655122	-3.524113
H	-0.624861	-3.572361	-2.163571
C	-1.163766	2.979372	2.907861
H	-1.759421	3.655112	3.524115
H	-0.624884	3.572361	2.163574
C	6.028106	0.332764	-1.157038
H	6.566988	0.586466	-2.063698
C	6.726258	0.000010	0.000003
C	-0.190618	-2.116950	-3.710844
H	0.718756	-2.644807	-4.003637
H	-0.674426	-1.738698	-4.616605
C	-2.013778	1.931836	2.217058
H	-2.811668	1.549939	2.860863

H	-2.453572	2.252957	1.274283
C	-4.235535	-0.000008	-0.000004
C	-0.190635	2.116949	3.710844
H	0.718736	2.644811	4.003640
H	-0.674442	1.738692	4.616604
H	4.089025	0.599316	-2.053489
H	7.811015	0.000011	0.000003
H	4.089025	-0.599304	2.053492
H	-4.635477	4.744859	-1.317017
H	-0.576378	5.176294	-0.002724
H	-1.845497	1.561226	-1.945815
H	-4.635463	-4.744875	1.317017
H	-1.845494	-1.561231	1.945812
H	-0.576361	-5.176296	0.002726

	77		
LMg(THF)2NC		B3PW91/6-311+G**	
Mg	0.996986	0.001518	-0.001665
O	1.102446	-0.494195	2.121274
O	1.093321	0.495314	-2.125701
N	-0.469025	1.502150	0.299336
N	-0.466224	-1.502763	-0.297695
N	3.039462	0.005880	-0.005442
C	-1.829846	1.258463	0.139381
C	-0.324303	2.838442	0.362801
C	0.968523	-3.509928	-0.559693
C	-0.318527	-2.838767	-0.360610
C	-2.440991	-0.002440	0.002082
C	-1.827323	-1.262064	-0.135963
C	2.008019	-2.912998	-1.283248
C	0.961390	3.512547	0.560718
C	-3.935267	-0.004080	0.003138
C	-2.524465	2.506041	0.102220
H	-3.590137	2.630669	-0.015976
C	3.221472	-3.567379	-1.456316
H	4.019690	-3.076198	-2.001888
C	-1.580329	3.494405	0.232631
H	-1.747808	4.560896	0.272478
C	2.003037	2.917817	1.282979
C	-4.647845	-0.380718	-1.139396
C	3.414972	-4.838538	-0.925817
C	-4.647060	0.370956	1.146689
C	-0.063982	0.509858	-2.989570
H	-0.765761	1.265762	-2.620307
H	-0.536634	-0.471511	-2.928606
C	1.165450	4.797724	0.033098
C	-1.572882	-3.497484	-0.228403
H	-1.738026	-4.564372	-0.267408
C	1.176236	-4.794384	-0.031738

C	2.385155	-5.451164	-0.214490
H	2.529033	-6.440806	0.207516
C	-2.519086	-2.511160	-0.097247
H	-3.584306	-2.638127	0.022532
C	-0.053376	-0.518578	2.986975
H	-0.749915	-1.279315	2.617773
H	-0.533564	0.459261	2.927971
C	2.372945	5.457368	0.214937
H	2.514025	6.447514	-0.206829
C	2.219783	-1.168418	2.761380
H	2.978476	-0.415953	2.992053
H	2.646616	-1.875833	2.051195
C	3.404910	4.846969	0.925056
C	3.215018	3.575127	1.455206
H	4.014994	3.085727	1.999803
C	-6.038868	-0.374774	-1.140801
H	-6.577110	-0.658720	-2.039406
C	1.640328	-1.810968	4.013533
H	2.384128	-1.899851	4.807979
H	1.260359	-2.812689	3.790583
C	1.617689	1.814548	-4.020183
H	2.359743	1.908331	-4.815697
H	1.229782	2.813459	-3.798287
C	-6.038062	0.361924	1.150064
H	-6.575657	0.644666	2.049435
C	-6.738032	-0.007199	0.005126
C	0.486122	-0.870730	4.363342
H	-0.272730	-1.331840	4.999051
H	0.858526	0.025077	4.870368
C	2.204006	1.178642	-2.767784
H	2.969112	0.432625	-2.998063
H	2.625178	1.890853	-2.058969
C	4.212818	0.007768	-0.008268
C	0.470763	0.864415	-4.367150
H	-0.292650	1.318407	-5.002526
H	0.849798	-0.029074	-4.873351
H	-4.102331	0.662443	2.038490
H	-7.823171	-0.008403	0.005896
H	-4.103737	-0.671023	-2.031961
H	4.352240	5.358528	1.059990
H	0.375489	5.271134	-0.540656
H	1.861439	1.939197	1.727841
H	4.363411	-5.347853	-1.061456
H	1.863692	-1.935004	-1.728621
H	0.387995	-5.269477	0.542988

	77		
LMg(THF)2CN		B3PW91/6-311+G**	
Mg	-0.991154	-0.000055	0.000028

O	-1.082597	0.514782	2.130089
O	-1.082733	-0.514738	-2.130086
N	0.477612	-1.498001	0.309398
N	0.477526	1.497972	-0.309432
N	-4.321219	-0.000004	0.000094
C	1.838399	-1.259169	0.143625
C	0.328798	-2.833392	0.377403
C	-0.959982	3.499947	-0.581828
C	0.328645	2.833357	-0.377434
C	2.451761	0.000042	-0.000017
C	1.838327	1.259220	-0.143646
C	-1.991743	2.900072	-1.314235
C	-0.959787	-3.500060	0.581806
C	3.945753	0.000085	-0.000026
C	2.528872	-2.509131	0.107478
H	3.593684	-2.637580	-0.014521
C	-3.208928	3.546810	-1.489871
H	-4.001561	3.052554	-2.040650
C	1.581871	-3.494194	0.244141
H	1.745641	-4.561217	0.286133
C	-1.991569	-2.900269	1.314253
C	4.658029	0.379099	-1.142061
C	-3.413209	4.813892	-0.953611
C	4.658071	-0.378887	1.141998
C	0.073494	-0.535580	-2.995129
H	0.777503	-1.286235	-2.619353
H	0.543993	0.447393	-2.944310
C	-1.177833	-4.780710	0.049428
C	1.581679	3.494230	-0.244154
H	1.745387	4.561263	-0.286138
C	-1.178116	4.780575	-0.049431
C	-2.390624	5.430186	-0.235144
H	-2.543102	6.416927	0.190628
C	2.528731	2.509218	-0.107490
H	3.593535	2.637725	0.014521
C	0.073682	0.536003	2.995031
H	0.777328	1.287084	2.619410
H	0.544626	-0.446742	2.943948
C	-2.390275	-5.430426	0.235198
H	-2.542683	-6.417185	-0.190558
C	-2.189879	1.214455	2.760409
H	-2.963716	0.478209	2.993270
H	-2.601034	1.925550	2.043919
C	-3.412879	-4.814222	0.953714
C	-3.208686	-3.547118	1.489958
H	-4.001327	-3.052946	2.040802
C	6.049018	0.371506	-1.144516
H	6.586930	0.657149	-2.042779
C	-1.604422	1.857309	4.010036

H	-2.348609	1.960560	4.802334
H	-1.211394	2.852719	3.781703
C	-1.604387	-1.857461	-4.009872
H	-2.348472	-1.961069	-4.802220
H	-1.211271	-2.852745	-3.781145
C	6.049060	-0.371208	1.144430
H	6.587003	-0.656819	2.042685
C	6.748598	0.000171	-0.000048
C	-0.462659	0.904942	4.368123
H	0.301023	1.361296	5.001507
H	-0.846900	0.017573	4.881102
C	-2.190030	-1.214226	-2.760560
H	-2.963624	-0.477846	-2.993837
H	-2.601566	-1.925032	-2.044003
C	-3.157641	-0.000064	0.000106
C	-0.462709	-0.905057	-4.368145
H	0.301093	-1.361504	-5.001316
H	-0.847027	-0.017914	-4.881459
H	4.113678	-0.672067	2.033436
H	7.833740	0.000206	-0.000057
H	4.113603	0.672241	-2.033491
H	-4.364258	-5.317659	1.090587
H	-0.395222	-5.257984	-0.531226
H	-1.836128	-1.926413	1.765439
H	-4.364644	5.317239	-1.090426
H	-1.836226	1.926229	-1.765424
H	-0.395524	5.257912	0.531196

	77		
LMg(THF)2NC		B3PW91/6-311+G(2df,p)	
Mg	0.980277	-0.000292	0.000241
O	1.083619	-0.464820	2.147462
O	1.084924	0.464656	-2.146801
N	-0.454683	1.511620	0.281080
N	-0.455313	-1.511495	-0.281271
N	3.007945	-0.001054	0.000714
C	-1.819274	1.261814	0.127121
C	-0.311671	2.848427	0.341398
C	0.969843	-3.533294	-0.531893
C	-0.312934	-2.848365	-0.341655
C	-2.429228	0.000524	-0.000307
C	-1.819826	-1.261043	-0.127610
C	2.015185	-2.960861	-1.265023
C	0.971414	3.532730	0.531808
C	-3.927742	0.000875	-0.000470
C	-2.512246	2.509808	0.092610
H	-3.576469	2.636227	-0.016203
C	3.218929	-3.631758	-1.431670
H	4.020080	-3.158832	-1.985670

C	-1.570695	3.498355	0.215019
H	-1.741048	4.562528	0.252250
C	2.016356	2.959826	1.265145
C	-4.640166	-0.317967	-1.158415
C	3.399864	-4.895212	-0.882610
C	-4.640255	0.320062	1.157326
C	-0.053014	0.422122	-3.040184
H	-0.783864	1.167383	-2.712632
H	-0.499153	-0.568353	-2.962527
C	1.167791	4.811203	-0.013246
C	-1.572300	-3.497698	-0.215594
H	-1.743154	-4.561786	-0.252941
C	1.165473	-4.811920	0.013070
C	2.365638	-5.484045	-0.160474
H	2.498722	-6.466952	0.276029
C	-2.513401	-2.508710	-0.093326
H	-3.577709	-2.634626	0.015235
C	-0.054627	-0.421008	3.040404
H	-0.786210	-1.165384	2.712484
H	-0.499583	0.569991	2.962651
C	2.368283	5.482717	0.160403
H	2.501939	6.465514	-0.276173
C	2.187042	-1.180462	2.777691
H	2.993438	-0.465967	2.947698
H	2.544711	-1.939195	2.084792
C	3.402106	4.893410	0.882730
C	3.220433	3.630103	1.431888
H	4.021255	3.156803	1.986043
C	-6.030073	-0.310575	-1.161037
H	-6.566577	-0.550526	-2.071306
C	1.619671	-1.744462	4.076107
H	2.380801	-1.821648	4.852934
H	1.202325	-2.740925	3.912284
C	1.623085	1.743750	-4.075249
H	2.384676	1.820065	-4.851709
H	1.206650	2.740654	-3.911789
C	-6.030167	0.313336	1.159657
H	-6.566746	0.553546	2.069814
C	-6.729535	0.001548	-0.000763
C	0.504063	-0.750326	4.417076
H	-0.254605	-1.163421	5.082659
H	0.917067	0.146219	4.886331
C	2.189266	1.179392	-2.776458
H	2.995194	0.464246	-2.945927
H	2.547133	1.937912	-2.083425
C	4.176038	-0.001378	0.001030
C	0.506646	0.750687	-4.416634
H	-0.251286	1.164465	-5.082632
H	0.918967	-0.146338	-4.885570

H	-4.099054	0.569618	2.061771
H	-7.812871	0.001809	-0.000877
H	-4.098896	-0.567781	-2.062748
H	4.343159	5.414104	1.012220
H	0.377405	5.270202	-0.594330
H	1.885864	1.987454	1.720510
H	4.340662	-5.416387	-1.012022
H	1.885258	-1.988365	-1.720281
H	0.374764	-5.270562	0.593997

	77		
LMg(THF)2CN		B3PW91/6-311+G(2df,p)	
Mg	-0.972403	0.000035	0.000030
O	-1.071108	0.479540	2.154885
O	-1.071268	-0.479521	-2.154801
N	0.465564	-1.508412	0.289055
N	0.465636	1.508397	-0.289079
N	-4.289226	0.000167	0.000129
C	1.829973	-1.260784	0.130856
C	0.320865	-2.844814	0.351875
C	-0.963546	3.525332	-0.546261
C	0.321010	2.844807	-0.351908
C	2.440462	-0.000060	-0.000037
C	1.830036	1.260695	-0.130915
C	-2.001606	2.951583	-1.288839
C	-0.963726	-3.525267	0.546246
C	3.938736	-0.000101	-0.000057
C	2.521042	-2.509822	0.096285
H	3.584771	-2.637943	-0.015462
C	-3.209264	3.615254	-1.456093
H	-4.005186	3.140163	-2.015601
C	1.578309	-3.496962	0.222726
H	1.746882	-4.561417	0.260801
C	-2.001739	-2.951467	1.288849
C	4.651295	0.324176	-1.156495
C	-3.400528	4.873779	-0.899235
C	4.651308	-0.324416	1.156364
C	0.063510	-0.428371	-3.051322
H	0.801411	-1.168220	-2.726871
H	0.502065	0.565493	-2.974274
C	-1.169603	-4.799053	-0.005707
C	1.578493	3.496886	-0.222797
H	1.747124	4.561332	-0.260887
C	-1.169337	4.799138	0.005677
C	-2.373084	5.464729	-0.169252
H	-2.514422	6.443984	0.272840
C	2.521175	2.509696	-0.096372
H	3.584914	2.637759	0.015346
C	0.063712	0.428254	3.051346

H	0.801687	1.168008	2.726847
H	0.502140	-0.565665	2.974283
C	-2.373387	-5.464574	0.169233
H	-2.514792	-6.443814	-0.272869
C	-2.164691	1.220076	2.774256
H	-2.989755	0.524556	2.932462
H	-2.496287	1.989919	2.079887
C	-3.400784	-4.873572	0.899240
C	-3.209435	-3.615066	1.456114
H	-4.005319	-3.139934	2.015641
C	6.041176	0.316977	-1.159033
H	6.577719	0.561013	-2.068189
C	-1.599659	1.770063	4.080343
H	-2.365747	1.852972	4.851621
H	-1.168718	2.762037	3.925019
C	-1.600062	-1.769993	-4.080229
H	-2.366211	-1.852813	-4.851458
H	-1.169212	-2.762013	-3.924951
C	6.041189	-0.317295	1.158865
H	6.577742	-0.561360	2.068007
C	6.740591	-0.000178	-0.000093
C	-0.498793	0.760818	4.425427
H	0.260617	1.162909	5.096900
H	-0.925701	-0.131917	4.889428
C	-2.164954	-1.219971	-2.774095
H	-2.989976	-0.524386	-2.932230
H	-2.496557	-1.989796	-2.079708
C	-3.130141	0.000125	0.000093
C	-0.499116	-0.760855	-4.425372
H	0.260208	-1.163015	-5.096900
H	-0.925962	0.131932	-4.889331
H	4.110088	-0.578061	2.059642
H	7.823927	-0.000208	-0.000107
H	4.110066	0.577850	-2.059760
H	-4.344640	-5.389162	1.028591
H	-0.384326	-5.258734	-0.593207
H	-1.861393	-1.983915	1.751997
H	-4.344354	5.389424	-1.028577
H	-1.861325	1.984015	-1.751972
H	-0.384022	5.258780	0.593157

	4		
HMgNC		B3PW91/6-311+G**	
Mg	-0.002794	-1.176654	0.000000
C	0.006334	1.940449	0.000000
H	-0.004476	-2.861497	0.000000
N	0.000000	0.762665	0.000000

	4

HMgNC TS		B3PW91/6-311+G**	
Mg	-0.998789	-0.028097	-0.004931
C	1.385718	-0.535272	0.001228
H	-2.668126	-0.251787	0.032520
N	0.905613	0.542941	0.002755
	4		
HMgNC SideOn		B3PW91/6-311+G**	
Mg	-0.973280	-0.081348	-0.000005
C	1.244679	-0.563284	0.000005
H	-2.620408	0.276554	0.000037
N	0.975957	0.582760	0.000000
	4		
HMgCN TS		B3PW91/6-311+G**	
Mg	-0.587108	-0.907935	0.000000
C	0.000000	1.057099	0.000000
H	-1.126858	-2.504917	0.000000
N	1.167451	1.008221	0.000000
	4		
HMgCN		B3PW91/6-311+G**	
Mg	0.000000	0.000000	-1.280031
C	0.000000	0.000000	0.784771
H	0.000000	0.000000	-2.966563
N	0.000000	0.000000	1.945472
	77		
TS LMg(THF)2CN		B3PW91/6-311+G** GD3BJ	
Mg	-0.712374	0.289031	0.481321
O	0.676152	0.485784	2.090572
O	-2.166215	-0.070338	-0.967174
N	0.159531	-1.576997	0.070438
N	0.719483	1.299937	-0.668886
C	1.498455	-1.654490	-0.288454
C	-0.327973	-2.830984	0.034491
C	-0.340005	3.511307	-0.870720
C	0.823932	2.631828	-0.819790
C	2.356046	-0.565155	-0.537075
C	2.014578	0.788433	-0.676372
C	-1.543320	3.078614	-1.437105
C	-1.728618	-3.156288	0.300243
C	3.792068	-0.905518	-0.709094
C	1.837023	-3.021764	-0.522219
H	2.807412	-3.385345	-0.824321
C	-2.648739	3.915341	-1.469207
H	-3.576045	3.568560	-1.913085
C	0.685770	-3.756147	-0.338808
H	0.567326	-4.826218	-0.427118

C	-2.496532	-2.437764	1.221955
C	4.454978	-0.639108	-1.909625
C	-2.572395	5.197830	-0.935607
C	4.496334	-1.509128	0.335873
C	-1.828253	-0.684246	-2.233623
H	-1.402528	-1.667483	-2.026162
H	-1.067037	-0.065625	-2.713932
C	-2.340229	-4.206958	-0.399915
C	2.188692	3.025348	-0.915959
H	2.548679	4.032607	-1.066530
C	-0.268589	4.807734	-0.348353
C	-1.377442	5.641395	-0.376579
H	-1.313725	6.637299	0.048848
C	2.935063	1.871107	-0.829426
H	4.010007	1.782001	-0.868069
C	0.525314	-0.324103	3.270229
H	1.387968	-0.996447	3.338969
H	-0.383534	-0.917441	3.161171
C	-3.676859	-4.518615	-0.190407
H	-4.133736	-5.329270	-0.748433
C	1.111230	1.820721	2.449187
H	0.287667	2.506612	2.230857
H	1.972548	2.066104	1.826248
C	-4.432600	-3.786883	0.722816
C	-3.834406	-2.746302	1.427582
H	-4.406751	-2.168805	2.145910
C	5.795962	-0.971315	-2.061967
H	6.296697	-0.771112	-3.003286
C	1.415796	1.760829	3.938500
H	1.243913	2.721564	4.426844
H	2.457102	1.470243	4.110166
C	-4.160602	-0.901660	-1.863477
H	-5.176349	-0.639328	-2.165003
H	-4.166774	-1.917852	-1.459754
C	5.840937	-1.827598	0.187978
H	6.380152	-2.285146	1.010663
C	6.493389	-1.561728	-1.012269
C	0.466693	0.663120	4.420757
H	0.764861	0.216380	5.371290
H	-0.549673	1.054142	4.514074
C	-3.600031	0.058471	-0.834297
H	-3.870123	1.099543	-1.037586
H	-3.856366	-0.175153	0.196616
C	-3.140459	-0.777750	-2.995695
H	-3.150592	-1.625021	-3.683483
H	-3.319725	0.134381	-3.573463
H	3.979642	-1.717630	1.266076

H	7.541292	-1.816267	-1.130147
H	3.905427	-0.182892	-2.725438
H	-5.477917	-4.027639	0.884042
H	-1.763346	-4.764072	-1.130247
H	-2.061528	-1.624692	1.789838
H	-3.440072	5.848442	-0.952827
H	-1.597710	2.081323	-1.852837
H	0.654689	5.147895	0.108286
C	-2.239240	0.905640	1.964918
N	-2.118822	2.045507	1.729574

	77		
TS LMg(THF)2CN		B3PW91/6-311+G**	
Mg	-0.750286	0.323960	0.500028
O	0.516994	0.259636	2.287335
O	-2.158259	0.277146	-1.122525
N	-0.060970	-1.611159	-0.025656
N	0.830042	1.322609	-0.484539
C	1.277695	-1.768140	-0.371996
C	-0.622938	-2.833033	-0.114367
C	0.114816	3.706840	-0.609561
C	1.114676	2.636117	-0.578717
C	2.233833	-0.745924	-0.537642
C	2.048258	0.648440	-0.557286
C	-1.154847	3.514551	-1.165189
C	-2.034434	-3.138700	0.148387
C	3.634947	-1.216286	-0.765092
C	1.529593	-3.143690	-0.656320
H	2.478318	-3.561694	-0.956612
C	-2.067647	4.558964	-1.228818
H	-3.047205	4.393076	-1.665635
C	0.336712	-3.806284	-0.509249
H	0.155519	-4.864102	-0.631728
C	-2.806992	-2.429589	1.075901
C	4.258281	-1.037571	-2.003446
C	-1.733619	5.816145	-0.735439
C	4.341467	-1.852100	0.259775
C	-1.786121	-0.207673	-2.434570
H	-1.604300	-1.284259	-2.366540
H	-0.857934	0.290319	-2.721569
C	-2.642522	-4.203769	-0.537732
C	2.517278	2.846343	-0.690900
H	3.007675	3.801307	-0.811596
C	0.440530	4.979578	-0.117929
C	-0.474804	6.021297	-0.177037
H	-0.207901	6.995433	0.220100
C	3.101745	1.603936	-0.679631

H	4.153785	1.376200	-0.759008
C	0.253551	-0.665558	3.365144
H	0.962488	-1.496012	3.278852
H	-0.762944	-1.046463	3.251354
C	-3.968543	-4.543372	-0.304633
H	-4.415650	-5.367837	-0.851143
C	1.075406	1.489230	2.812121
H	0.293436	2.255174	2.786060
H	1.899318	1.782597	2.159389
C	-4.723615	-3.827210	0.621268
C	-4.135299	-2.768651	1.307004
H	-4.707321	-2.202273	2.035122
C	5.557830	-1.487816	-2.212878
H	6.024355	-1.351774	-3.183312
C	1.498701	1.160858	4.235058
H	1.507022	2.046202	4.874153
H	2.500626	0.719630	4.248712
C	-4.147336	-0.049760	-2.357631
H	-5.050178	0.472993	-2.680133
H	-4.392727	-1.107997	-2.229629
C	5.646321	-2.288811	0.054726
H	6.185787	-2.771178	0.863562
C	6.257162	-2.110531	-1.183096
C	0.448410	0.128913	4.648520
H	0.763706	-0.503441	5.481326
H	-0.484398	0.627577	4.926095
C	-3.588087	0.524438	-1.064348
H	-3.738456	1.604917	-0.984638
H	-3.974953	0.053476	-0.161431
C	-2.974379	0.111972	-3.326441
H	-3.030562	-0.554357	-4.189726
H	-2.915698	1.141372	-3.694130
H	3.862485	-1.996712	1.222385
H	7.272767	-2.456832	-1.345047
H	3.713373	-0.551643	-2.806013
H	-5.759164	-4.093428	0.806878
H	-2.071389	-4.758390	-1.274675
H	-2.383607	-1.602603	1.633195
H	-2.449932	6.629840	-0.781936
H	-1.416066	2.538574	-1.554833
H	1.413175	5.145427	0.333274
C	-2.359182	0.907286	1.921638
N	-2.139213	2.050331	1.800553

5) References

[1] E. R. King, T. A. Betley, *Inorg. Chem.* **2009**, *48*, 2361-2363.

[2] M. Westerhausen, W. Schwarz, *Z. Anorg. Allg. Chem*. **1992**, *609*, 39-44.

[3] M. T. Reetz, I. Chatziiosifidis, *Synthesis* **1982**, *4*, 330.

[4] Rigaku Oxford Diffraction, **2018**, CrysAlisPro Software system, version 1.171.39.46, Rigaku Corporation, Oxford, UK.

[5] O. V. Dolomanov, L. J. Bourhis, R.J. Gildea, J. A. K. Howard and H. Puschmann, *J. Appl. Cryst.,* **2009**, *42*, 339–341.

[6] G. M. Sheldrick, *Acta Cryst. A,* **2015**, *71*, 3–8.

[7] G. M. Sheldrick, *Acta Cryst.* **2008**, *A64*, 112-122.

[8] B. Rees, L. Jenner, M. Yusupov, *Acta Cryst.* **2005**, *D61*, 1299-1301.

[9] R. Neufeld, D. Stalke, *Chem. Sci.* **2015**, *6*, 3354-3364.

[10] S. Bachmann, R. Neufeld, M. Dzemski, D. Stalke, C*hem. Eur. J.* **2016**, *22*, 8462-8465.

[11] D. L. Pavia, G. M. Lampman, G. S. Kriz, J. R. Vyvyan, in *Introduction to Spectroscopy*. Stamford, CT: Cengage Learning, **2015**, Fifth Edition.

[12] J. P. Merrick, D. Moran, L. Radom, *J. Phys. Chem. A* **2007**, *111*, 11683-11700.

[13] M. J. Frisch, G. W. Trucks, H. B. Schlegel, G. E. Scuseria, M. A. Robb, J. R. Cheeseman, G. Scalmani, V. Barone, G. A. Petersson, H. Nakatsuji, X. Li, M. Caricato, A. V. Marenich, J. Bloino, B. G. Janesko, R. Gomperts, B. Mennucci, H. P. Hratchian, J. V. Ortiz, A. F. Izmaylov, J. L. Sonnenberg, D. Williams-Young, F. Ding, F. Lipparini, F. Egidi, J. Goings, B. Peng, A. Petrone, T. Henderson, D. Ranasinghe, V. G. Zakrzewski, J. Gao, N. Rega, G. Zheng, W. Liang, M. Hada, M. Ehara, K. Toyota, R. Fukuda, J. Hasegawa, M. Ishida, T. Nakajima, Y. Honda, O. Kitao, H. Nakai, T. Vreven, K. Throssell, J. A. Montgomery, J. E. Peralta, F. Ogliaro, M. J. Bearpark, J. J. Heyd, E. N. Brothers, K. N. Kudin, V. N. Staroverov, T. A. Keith, R. Kobayashi, J. Normand, K. Raghavachari, A. P. Rendell, J. C. Burant, S. S. Iyengar, J. Tomasi, M. Cossi, J. M. Millam, M. Klene, C. Adamo, R. Cammi, J. W. Ochterski, R. L. Martin, K. Morokuma, O. Farkas, J. B. Foresman, D. J. Fox, Gaussian 16 Rev. A.03, Wallingford CT, **2016**.

[14] A. D. Becke, *Phys. Rev. A* **1988**, *38*, 3098–3100.

[15] J. P. Perdew, *Electronic Structure of Solids*, Akademie Verlang, Berlin, **1991**.

[16] W. J. Hehre, L. Radom, P. v. R. Schleyer, J. A. Pople, Ab Initio Molecular Orbital Theory by W. J. Hehre, L. Radom, P. v. R. Schleyer, and J. A. Pople, John Wiley, New York, John Wiley, New York, **1986**.

[17] T. Clark, J. Chandrasekhar, G. W. Spitznagel, P. v. R. Schleyer, *J. Comp. Chem*. **1983**, *4*, 294–301.

[18] S. Grimme, S. Ehrlich, L. Goerigk*, J. Comp. Chem*. **2011**, *32*, 1456–1465.

[19] A. E. Reed, R. B. Weinstock, F. Weinhold, *J. Chem. Phys.* **1985**, *83*, 735–746.

[20] N. van Eikema Hommes, *Molecule*, Erlangen, **2016**.

[21] R. F. W. Bader, *Chem. Rev*. **1991**, *91*, 893–928.

[22] T. A. Keith, AIMAll (Version 17.01.25), TK Gristmill Software, Overland Park KS USA, **2017**.
